# Rapid flow-based synthesis of post-translationally modified peptides and proteins: a case study on MYC's transactivation domain†

**DOI:** 10.1039/d4sc00481g

**Published:** 2024-05-07

**Authors:** Elyse T. Williams, Kevin Schiefelbein, Matthias Schuster, Ikhlas M. M. Ahmed, Marije De Vries, Rebecca Beveridge, Oliver Zerbe, Nina Hartrampf

**Affiliations:** a Department of Chemistry, University of Zurich Winterthurerstrasse 190 8057 Zurich Switzerland nina.hartrampf@chem.uzh.ch; b Department for Pure and Applied Chemistry, University of Strathclyde 295 Cathedral St Glasgow G1 1XL UK

## Abstract

Protein–protein interactions of c-Myc (MYC) are often regulated by post-translational modifications (PTMs), such as phosphorylation, and crosstalk thereof. Studying these interactions requires proteins with unique PTM patterns, which are challenging to obtain by recombinant methods. Standard peptide synthesis and native chemical ligation can produce such modified proteins, but are time-consuming and therefore typically limited to the study of individual PTMs. Herein, we report the development of flow-based methods for the rapid synthesis of phosphorylated MYC sequences (up to 84 AA), and demonstrate the versatility of this approach for the incorporation of other PTMs (*N*^ε^-methylation, sulfation, acetylation, glycosylation) and combinations thereof. Peptides containing up to seven PTMs and phosphorylation at up to five sites were successfully prepared and isolated in high yield and purity. We further produced ten PTM-decorated analogues of the MYC Transactivation Domain (TAD) to screen for binding to the tumor suppressor protein, Bin1, using heteronuclear NMR and native mass spectrometry. We determined the effects of phosphorylation and glycosylation on the strength of the MYC:Bin1 interaction, and reveal an influence of MYC sequence length on binding. Our platform for the rapid synthesis of MYC sequences up to 84 AA with distinct PTM patterns thus enables the systematic study of PTM function at a molecular level, and offers a convenient way for expedited screening of constructs.

## Abstract 2

Automated flow peptide synthesis (AFPS) gives access to peptides and proteins with post-translational modifications (PTMs) and enables investigation of PTM-mediated regulation, and was used to study the PTM-dependence of the MYC:Bin1 interaction.

## Graphical abstract

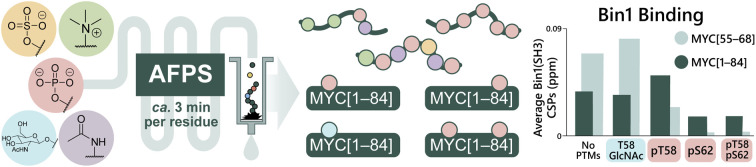

## Introduction

**Fig. 1:**
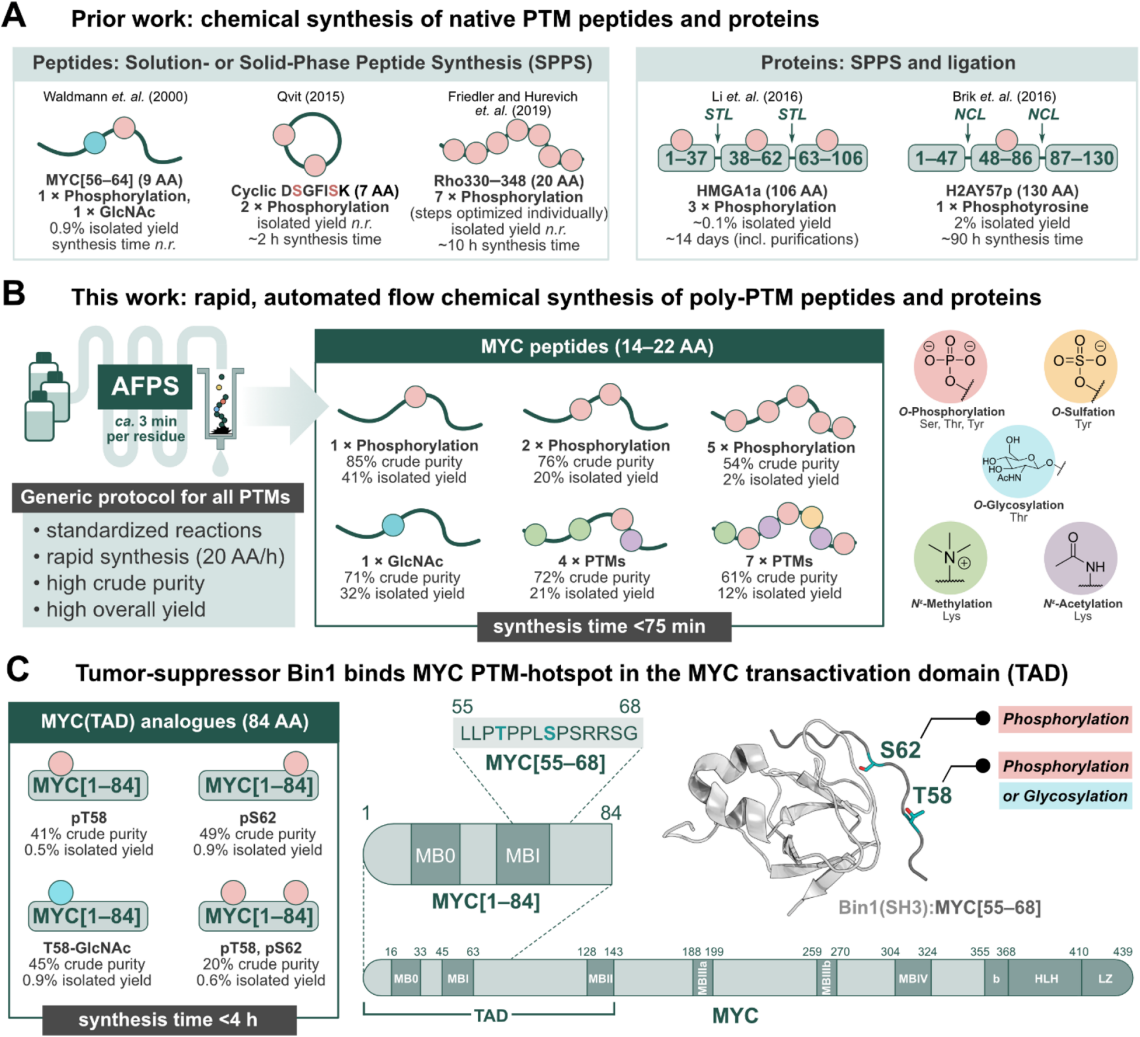
Peptides and proteins containing PTMs have remained a synthetic challenge for several decades. Automated fast-flow peptide/protein synthesis (AFPS) provides an avenue for rapid production of PTM-containing sequences, such as the MYC N-terminus. (A) Prior literature reports for the synthesis of peptides and proteins containing post-translational modifications (PTMs).^19–21^ STL = serine/threonine ligation, NCL = native chemical ligation. (B) This work, utilizing AFPS^27^ for the production of polyphosphorylated and poly-PTM containing peptides. (C) The tumor-suppressing protein, Bridging Integrator 1 (Bin1), binds to the MYC N-terminal transactivation domain (TAD) at a PTM-hotspot (PDB: 1MV0).^31,32^ The MYC residue S62 undergoes phosphorylation, and residue T58 can undergo phosphorylation or glycosylation (GlcNAc).^33–37^ Using our optimized AFPS methods for PTM incorporation, we prepared a series of MYC[1–84] protein fragments containing phosphorylation at S62 and/or T58, and GlcNAcylation at T58. These analogues were then applied in the study of PTM-mediated regulation of the MYC:Bin1 interaction. Isolated yield = overall yield of pure peptides or proteins based on resin loading. Synthesis time excludes resin cleavage and purification steps.

Post-translational modifications (PTMs) of proteins play an important role in regulating biological processes, and can influence protein–protein interactions (PPIs), signaling, conformational preferences, or phase separation.^1–5^ These modifications may operate on their own or in concert with others, known as PTM crosstalk.^6–10^ Furthermore, their installation and removal can be dynamic, and many different patterns may (co)exist for a single protein.^2,10^ The complex nature of PTM-mediated protein regulation is therefore difficult to investigate, and their study requires the production of proteins with specific PTM patterns.^11–13^ Recombinant expression in conjunction with enzymatic modification (*e.g.* phosphorylation by kinases) can be used to obtain such proteins, but the precise control of location and number of PTMs is challenging.^14,15^ Chemical peptide synthesis, on the other hand, allows for the incorporation of non-canonical amino acids (*e.g.* PTM-amino acids) in a site-specific manner.^11–18^ PTMs can be installed on a synthetic peptide sequence through either a building block method or by late-stage modification of the full-length peptide or protein.^11–18^ The former, and more popular method has been exemplified in the successful production of PTM-peptides such as glycophosphonapeptide MYC[56–64],^19^ cyclic diphosphorylated DSGFISK peptide,^20^ and heptaphosphorylated Rho330–348 (ref. 21) (Fig. 1A), in which the PTM-amino acids were incorporated as building blocks during solution- or solid-phase peptide synthesis in batch (batch-SPPS). As batch-SPPS is typically limited to peptides of <50 amino acids (AA), native chemical ligation (NCL) or expressed protein ligation (EPL) are required to obtain longer sequences,^16,22–24^ as demonstrated for triphosphorylated HMGA1a^25^ and phosphotyrosine-containing H2AY57p^26^ (Fig. 1A). However, the overall process of batch-SPPS and ligation is very time-consuming, laborious, and relatively low-yielding, impeding the production of large numbers of PTM-peptides and proteins.^11–18^ In particular, researchers have long called for a general method for the synthesis of polyphosphorylated peptides with high yield and purity.^17^

Recently, automated fast-flow peptide synthesis (AFPS) has proved successful for the rapid, linear synthesis of proteins exceeding 200 AA at a rate of approx. 2–3 min per residue.^27–29^ While flow-based protein synthesis has been successfully used to install single PTMs (phosphorylation, acetylation) into a protein,^30^ the routine incorporation of several PTM amino acid building blocks into a single sequence has not yet been reported. Phosphorylation and polyphosphorylation, in particular, have posed a significant challenge in chemical peptide and protein synthesis due to difficult couplings (bulky side-chain protecting groups) and the occurrence of side-reactions (β-elimination of the phosphate) during SPPS, leading to large amounts of undesired side-products and low yields.^14,17^ With AFPS, conditions for activation, coupling, and deprotection steps of each amino acid can be rapidly screened and evaluated by in-line UV-Vis analysis, which can then be corroborated with LCMS analysis of peptide products to identify optimum conditions.^27^ AFPS therefore has the capacity to be a well-suited method for the chemical synthesis of polyphosphorylated peptides and proteins, and can potentially be extended to many other PTMs.

The intrinsically disordered transcription factor c-Myc (MYC) is tightly regulated through PTMs, particularly phosphorylation.^38–42^ MYC is involved in the regulation of cell growth and proliferation in humans and animals, and is the most amplified gene in cancer.^43,44^ PTMs are reported to play a major role in MYC activation and degradation, and understanding the regulation of MYC-PPIs through PTMs could therefore lead to new MYC-targeting strategies.^43^ However, only a few PTMs on MYC have been studied thus far, mainly due to the lack of suitable tools for their systematic investigation. Two important, well-investigated PTM sites for regulating MYC degradation and activation are S62 and T58, found in MYC homology box I (MBI).^33–35,39–43,45^ Phosphorylation at S62 is known to stabilize and drive MYC transcriptional activation,^33,39^ while phosphorylation at T58 initiates the degradation pathway of MYC.^34^ T58 can also undergo glycosylation (GlcNAc) by the *O*-GlcNAc transferase (OGT), stabilizing MYC.^35–37^ Thereby, T58 might serve as a phosphorylation/glycosylation switch,^36^ however, the impact of T58 glycosylation is not well understood. MBI (residues 45–65) is a binding hub for many MYC-PPIs, such as the interaction with the tumor suppressor protein, Bin1 (Bridging integrator-1, also known as amphiphysin II) at MYC residues 61–63.^46,47^ In healthy cells, the Src-homology 3 (SH3) domain of Bin1 binds to MYC's N-terminal TAD and facilitates its degradation, thereby inhibiting cell proliferation.^31,32,46,47^ Prior research suggests that phosphorylation at MYC-S62 blocks the interaction with Bin1 (SH3), but phosphorylation at T58 is well tolerated.^31,32^ To the best of our knowledge, the effect of MYC T58-GlcNAcylation on the interaction with Bin1 is not yet described. Overall, phosphorylation at MYC T58/S62 has been subject to many studies, although the biological function of several neighboring phosphorylation sites remain unclear.^40,48^

Short fragments of proteins such as MYC may not fully represent the interactions of the full-length protein, therefore longer fragments containing PTMs should also be investigated. Binding proteins may interact with multiple sites dispersed across the MYC sequence, such as the proposed secondary Bin1 binding site at MYC residues 42–45.^31^ Furthermore, MYC is proposed to interact with itself, either intra- or intermolecularly.^49^ Short peptide fragments may lack this ability, and thereby exhibit different binding behaviors compared to the native protein. To provide a deeper understanding of MYC regulation through PTMs, methods to rapidly synthesize (poly)phosphorylated and other PTM-containing MYC peptides—applicable to the production of longer (*e.g.* >80 AA) fragments—are therefore required.

Herein, we set out to develop general synthesis protocols for AFPS that would allow for the rapid incorporation of multiple phosphorylated residues as well as four additional biologically relevant PTMs (methylation, acetylation, sulfation, and glycosylation) into synthetic peptides and proteins (Fig. 1B). A series of short (14–22 AA) and long (84 AA) MYC fragments with multiple PTMs were synthesized in high yield and purity. To demonstrate the utility of our approach for studying PTMs, we investigated MYC's binding interactions with Bin1 (Fig. 1C)^31,32,46,47^ using two biophysical techniques; heteronuclear NMR and native mass spectrometry (nMS). The combination of nMS and NMR has recently been shown to be highly effective in examining the effect of PTMs and small molecules on PPIs.^50^ In nMS, non-covalent interactions are maintained within protein complexes,^51^ providing qualitative information on the extent of binding between Bin1 and various MYC peptides. Through this, we observed distinct effects on the MYC:Bin1 interaction depending on MYC's PTM-state and sequence-length. Importantly, this report marks the first biophysical investigation of the MYC phosphorylation/glycosylation switch site at T58.

## Results

### Flow-based peptide synthesis provides access to polyphosphorylated MYC peptides

**Fig. 2:**
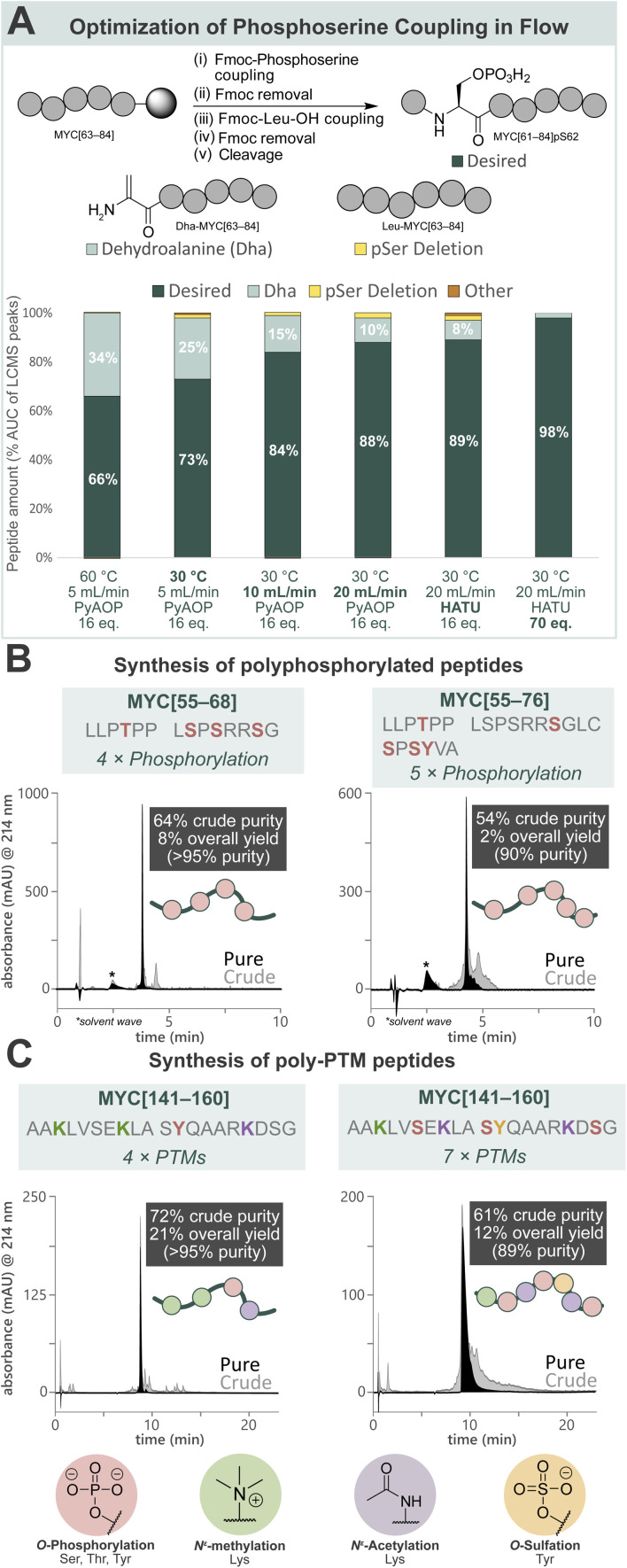
Development of a general method for the incorporation of phosphorylated amino acids and other common PTMs by AFPS provides access to poly-PTM peptides. (A) Optimization of AFPS conditions for the incorporation of phosphoserine (as Fmoc-Ser(PO(OBzl)OH)–OH) into MYC[61–84]pS62. Parameters investigated: pre-activation temperature, overall flow rate, coupling agent (PyAOP or HATU) and equivalents of the phosphoserine building block. Further elongation of the Dha peptide (Dha-MYC[63–84]) was not observed. Piperidinyl adducts of Dha were also not observed. (B) Polyphosphorylated MYC peptides (MYC[55–68]pT58, pS62, pS64, pS67 and MYC[55–76]pT58, pS67, pS71, pS73, pY74) prepared using the optimized AFPS methods. (C) Poly-PTM MYC peptides (MYC[141–160] Me_3_K143, Me_3_K148, pY152, AcK157 and MYC[141–160]Me_3_K143, pS146, AcK148, pS151, sY152, AcK157, pS159) prepared using the optimized AFPS methods. Me_3_K = *N*^ε^-trimethyllysine, AcK = acetyllysine, pY = phosphotyrosine, sY = sulfatyrosine. *Solvent wave.^55^

Towards a general platform for the synthesis of a wide range of PTM-containing peptides and proteins, a method for the incorporation of challenging phosphorylated amino acids was developed. Using Fmoc-Ser(PO(OBzl)OH)–OH (Fmoc-pSer(Bzl)-OH, 0.20 M), coupling agent (HATU or PyAOP, 0.19 M), and DIPEA (0.27 M) in DMF, the reaction parameters (pre-activation temperature, flow rate, coupling agent, and equivalents) for phosphoserine incorporation into a model peptide (MYC[61–84]pS62) by AFPS were optimized (Fig. 2A, see ESI† Section 4.2). Initially, pre-activation of Fmoc-pSer(Bzl)-OH with PyAOP at 60 °C, with a flow rate of 5.0 mL min^−1^, showed significant 2,3-dehydroalanine (Dha) formation (34%) *via* β-elimination. Notably, Dha formation was not found to be influenced by the Fmoc-removal step under flow conditions, suggesting that β-elimination in flow occurs primarily during the activation and coupling of the phosphorylated amino acid.^17,52^ We therefore reduced the pre-activation temperature to 30 °C, which decreased Dha formation to 25%. We next shortened the pre-activation time from ∼6.5 s to ∼3.2 s by increasing the flow rate to 10 mL min^−1^ (see ESI Table S1†), and Dha formation was significantly decreased to 15%. Further shortening of the pre-activation time to ∼1.6 s (increasing flow rate to 20 mL min^−1^), again decreased Dha formation to 10%, however deletion of the pSer residue increased to 2%. As the flow rate is increased to achieve shorter pre-activation times, the resin residence time is decreased (*i.e.* from 18 s [10 mL min^−1^] to 9 s [20 mL min^−1^]), preventing complete coupling to the resin. To mitigate this, more equivalents of amino acid and coupling agents are required to capture remaining active sites. Therefore, HATU was investigated as a cheaper alternative to PyAOP, and was found to give comparable results (88% *vs.* 89% desired product). Finally, increasing the equivalents of Fmoc-pSer(Bzl)-OH and HATU successfully gave the desired product with high purity (98%) and low Dha formation (2%). The rapid β-elimination reaction to afford Dha from phosphorylated amino acids during SPPS has been a persistent challenge in phosphopeptide synthesis using the building block approach.^17^ AFPS enables rapid screening and fine-tuning of reaction parameters, thereby facilitating phosphopeptide synthesis using these building blocks.

With the successful synthesis of mono-phosphorylated MYC[61–84]pS62 in hand, the optimized conditions were then applied in the synthesis of tetra-phosphorylated MYC[55–68] and penta-phosphorylated MYC[55–76]. These fragments of MYC represent the phosphorylation hotspot that overlaps with MYC Box I (MBI), a conserved sequence in the regulatory transactivation domain (TAD) of MYC. The tetra-phosphorylated MYC[55–68] peptide was successfully prepared with high crude purity (64%) and excellent overall yield (8%, >95% purity) (Fig. 2B, left). To our delight, the penta-phosphorylated MYC[55–76] was also readily prepared using AFPS with high crude purity (54%) and sufficient overall yield (2%, 90% purity) (Fig. 2B, right). Each of these heavily phosphorylated MYC peptides was obtained within 75 min of synthesis time (excluding resin cleavage and purification steps). These optimized conditions for phosphopeptide synthesis by AFPS were next evaluated in the incorporation of a variety of other PTM-containing residues.

### MYC fragments with various PTM patterns including phosphorylation, sulfation, acetylation, and methylation can be rapidly synthesized

To broaden the scope of AFPS-mediated synthesis of modified peptides to include other PTMs, the incorporation of methylated, sulfated, acetylated amino acids, and combinations thereof using the optimized methods were investigated, using MYC[141–160] as a model peptide. To note, the PTM sites and type do not necessarily represent biologically relevant modifications. Firstly, MYC[141–160] containing modification at three sites (acetylation at K148 and K157, sulfation at Y152) was prepared by AFPS using Fmoc-Lys(Ac)-OH and Fmoc-Tyr(SO_3_nP)-OH (nP = neopentyl) building blocks. After resin cleavage, the neopentyl protecting group was removed by incubation in water overnight,^53^ and HPLC purification afforded the desired peptide in good overall yield (20%, >95% purity) (see ESI† Section 4.3.3). Next, the tetra-modified peptide MYC[141–160] containing Lys(*N*^ε^-Me_3_) at positions K143 and K148, Lys(*N*^ε^-Ac) at K157, and phosphotyrosine (pY) at position Y152, was also afforded in good overall yield (21%, >95% purity) (Fig. 2C, left). Finally, a highly modified peptide containing three phosphoserine residues (at positions S146, S151, and S159), Lys(*N*^ε^-Me_3_) at K143, Lys(*N*^ε^-Ac) at K148 and K157, and sulfatyrosine (sY) at Y152 was synthesized by AFPS. After resin cleavage, removal of the neopentyl protecting group of sulfatyrosine gave the heavily modified MYC[141–160] with excellent crude purity (61%), which was then isolated by HPLC in good overall yield (12%, 89% purity) (Fig. 2C, right). Each of the phosphorylated MYC[141–160] analogues were prepared within 1 h of synthesis time, excluding resin cleavage and purification steps.

### MYC[55–68] interacts with tumor suppressor Bridging Integrator-1 (Bin1) in a PTM-dependent manner

**Fig. 3:**
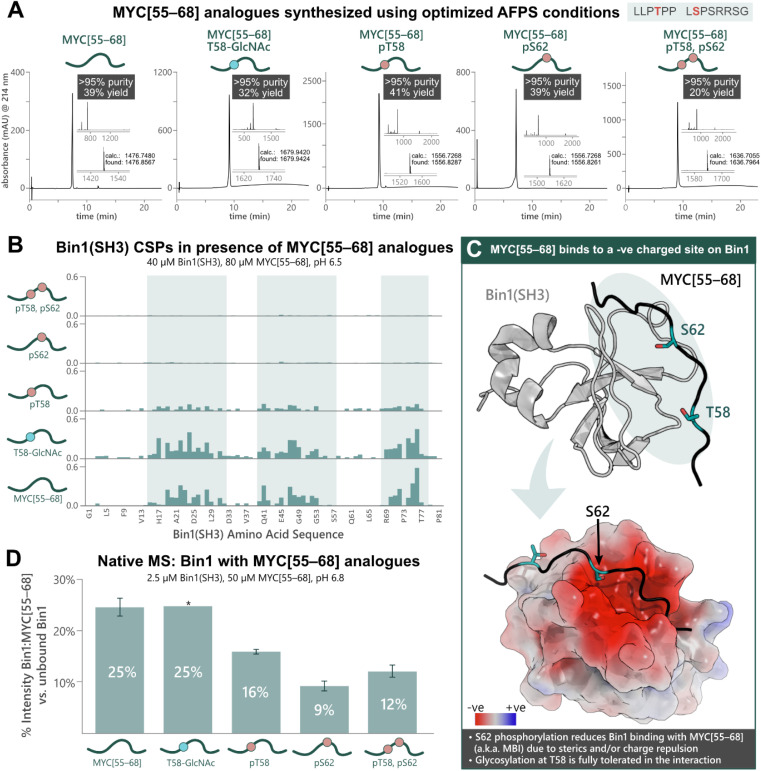
Chemical synthesis of PTM-containing MYC[55–68] derivatives enables biophysical analysis of the MYC:Bin1 interaction and the PTM-dependence of Bin1 binding to MYC[55–68]. (A) UHPLC profiles, purities, and overall yields (based on resin loading) of purified MYC[55–68] analogues prepared using the optimized AFPS methods. (B) Chemical shift perturbations (CSPs, ppm) of ^15^N-Bin1(SH3) backbone NH peaks in the presence of each MYC[55–68] analogue, measured by [^15^N,^1^H]-HSQC. Blue highlighting indicates Bin1 regions with greatest CSPs. (C) Unmodified MYC[55–68] binds at the negatively charged binding pocket of Bin1(SH3) (PDB: 1MV0);^32^ Top: 3D structure of Bin1(SH3) (grey) bound to MYC[55–68] (black). The blue oval indicates regions where most Bin1 CSPs were observed. Bottom: surface model of the Bin1 binding cleft with MYC[55–68] (black). The surface potential is shown for Bin1 (red and blue indicate negatively and positively charged surfaces, respectively). S62 of MYC[55–68] points directly into the negatively charged binding cleft, whereas T58 points slightly away from the binding interface.^32^ (D) nMS of Bin1(SH3) with each MYC[55–68] analogue, measured in triplicate*, wherein “% Intensity” denotes the percent of MYC:Bin1 complex present compared to the amount of free (unbound) Bin1(SH3), as measured by nMS peak intensity. Error bars represent the standard deviation of triplicate measurements. *nMS results of Bin1(SH3) with MYC[55–68]T58-GlcNAc are from a single measurement.

To apply our approach to the synthesis of PTM-peptides and their use in biophysical studies, we opted to investigate the PTM-dependence of MYC's interaction with Bin1. Five analogues of MYC[55–68] with phosphorylation at T58 and/or S62, or glycosylation at T58 were prepared using the previously optimized AFPS protocols (see ESI† Section 4.4). For the glycosylated peptide, Fmoc-Thr(β-d-GlcNAc(Ac)_3_)-OH (2.0 eq.) was coupled manually at position 58, and deprotection of the GlcNAc moiety was carried out on-resin using hydrazine in MeOH. The peptides MYC[55–68], MYC[55–68]pT58, MYC[55–68]pS62, MYC[55–68]pT58,pS62, and MYC[55–68]T58-GlcNAc were successfully obtained in high overall yield (20–41%, >95% purity) at multi-milligram scale (7–14 mg) (Fig. 3A). Each of the phosphorylated MYC[55–68] analogues were prepared within 1 h of synthesis time (excluding resin cleavage and purification steps). All peptides contained ^13^C and ^15^N at natural abundance. The strength of the interaction between the MYC[55–68] analogues with ^15^N-isotopically labeled Bin1 was qualitatively determined by [^15^N,^1^H]-heteronuclear NMR using chemical shift perturbations (CSPs).^54^ To this end, each MYC[55–68] analogue (80 μM) was incubated with ^15^N-labelled Bin1(SH3) (40 μM) in phosphate buffer (20 mM) at pH 6.5 (NMR buffer), and CSPs were measured by [^15^N,^1^H]-HSQC at 25 °C (see ESI† Section 7), carefully excluding artifacts from changes in pH upon addition of the synthetic MYC[55–68] analogues. All spectra of ^15^N-Bin1(SH3) in the presence of MYC[55–68] analogues displayed changes in the fast exchange regime on the NMR timescale. As expected, the unmodified MYC[55–68] analogue gave significant Bin1(SH3) CSPs for the amino acids in the known binding pocket of Bin1 (Fig. 3B).^32^ The glycosylated analogue, MYC[55–68]T58-GlcNAc resulted in slightly greater CSPs for Bin1(SH3) than unmodified MYC[55–68], indicating that T58-glycosylation of MYC is fully tolerated. Conversely, yet in agreement with the literature,^32^ minimal Bin1(SH3) CSPs were observed in the presence of MYC[55–68]pS62, due to charge repulsion and steric hindrance between the S62-phosphate and the negatively charged Bin1 binding pocket (Fig. 3C). Interestingly, the T58-monophosphorylated analogue also showed a decrease in Bin1 CSPs compared to unmodified MYC[55–68], although T58 points away from the binding cleft of Bin1 and is not in close proximity to negatively charged residues (Fig. 3C).^32^ To further support our observations, we next applied nMS to determine the effects of phosphorylation and glycosylation on the Bin1:MYC[55–68] interaction. Each MYC[55–68] peptide (50 μM) was incubated with Bin1(SH3) (2.5 μM) in ammonium acetate at pH 6.8, then measured *via* nanoelectrospray ionization (nESI) in positive mode (see ESI† Section 6). The intensities of the Bin1:MYC complexes relative to unbound Bin1 are shown as a percentage (Fig. 3D). In agreement with the literature^32^ and our NMR results, the unmodified MYC[55–68] gave the greatest relative signal intensity (25%) corresponding to binding, and S62-phosphorylated analogues showed the lowest intensity (9% for MYC[55–68]pS62, 12% for MYC[55–68]pT58,pS62). As with the NMR results, the pT58 monophosphorylated analogue showed a decrease in Bin1:MYC signal compared to Bin1 with the unmodified peptide (Fig. 3D).

### MYC's interactions with Bin1 not only depend on PTMs, but also on the length of MYC fragments

**Fig. 4:**
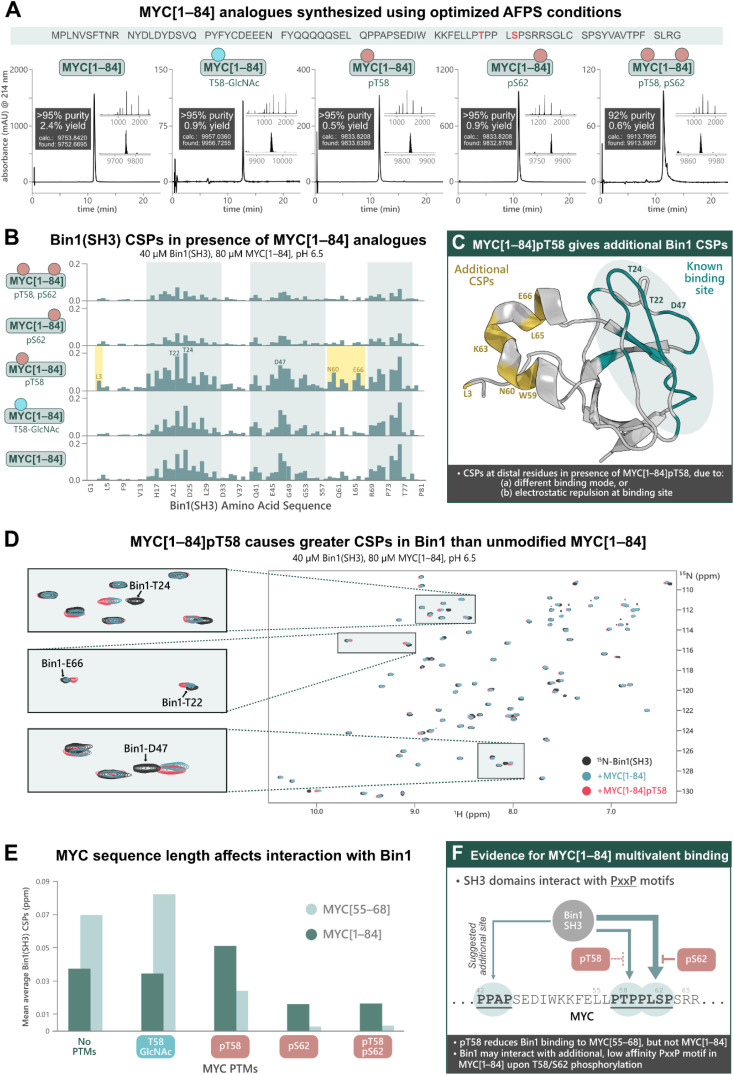
Chemical synthesis of PTM-containing MYC[1–84] derivatives enables biophysical analysis of their interactions with Bin1, which indicates an additional Bin1 binding site within MYC[1–84]. (A) UHPLC profiles, purities, and overall yield (from resin loading) of purified MYC[1–84] analogues prepared using the optimized AFPS methods. (B) CSPs (ppm) of ^15^N-Bin1(SH3) backbone NH peaks in the presence of each MYC[1–84] analogue, measured by [^15^N,^1^H]-HSQC, scaled to the highest peak. See ESI Fig. S54† for plot scaled to match Fig. 3B. (C) CSPs are observed at Bin1 residues (yellow) distal to the known MYC binding site (green, PDB: 1MV0)^32^ when MYC[1–84]pT58 is present. (D) [^15^N,^1^H]-HSQC of ^15^N-Bin1(SH3) in the apo state (black), ^15^N-Bin1(SH3) in the presence of MYC[1–84] (blue), and ^15^N-Bin1(SH3) in the presence of MYC[1–84]pT58 (red). MYC[1–84]pT58 induces greater Bin1 CSPs than the unmodified MYC[1–84] analogue, as shown for Bin1-T24 and Bin1-T22. Some residues, such as Bin1-D47, show CSPs in slightly different directions when in the presence of either MYC[1–84]pT58 or MYC[1–84]. (E) Mean average Bin1(SH3) CSPs in the presence of each MYC analogue investigated. (F) An additional Bin1 binding site on MYC (within residues 1–84) may exist. SH3 domains are known to interact with PxxP motifs, therefore the PPAP motif at residues 42–45 of the MYC N-terminus may interact with Bin1(SH3).^31^ Phosphorylation at S62 and/or T58 may prevent Bin1 binding to the primary site (residues 55–68) and promote Bin1 interaction at residues 42–45.

Previous research into the Bin1:MYC interaction suggests an additional Bin1 binding site in MYC (in addition to residues 55–68), although most biophysical studies into PTM-mediated regulation of IDPs have focused on short peptide fragments of MYC due to difficulties in obtaining longer sequences with PTMs.^31^ To uncover potential discrepancies in binding interactions of short (MYC[55–68]) compared to longer fragments, we synthesized MYC[1–84] and its PTM-containing analogues; MYC[1–84]pT58, MYC[1–84]pS62, MYC[1–84]pT58,pS62, MYC[1–84]T58-GlcNAc, by AFPS using our optimized protocols (see ESI† Section 4.5). Each of the MYC[1–84] analogues were obtained in under 4 h synthesis time (excluding resin cleavage and purification steps) with good crude purity (20–55%) and overall yield (0.5–2.4%, ∼170 steps, >92% purity) (Fig. 4A). The synthetic proteins were then applied in the following NMR experiments.

The MYC[1–84] analogues (80 μM) were each incubated with ^15^N-Bin1(SH3) (40 μM) in phosphate buffer (20 mM) at pH 6.5, then measured by [^15^N,^1^H]-HSQC at 25 °C (Fig. 4B, see ESI† Section 7), carefully excluding artifacts from changes in pH upon addition of the synthetic MYC[1–84] analogues. All spectra of ^15^N-Bin1(SH3) in the presence of MYC[1–84] analogues displayed changes in the fast exchange regime on the NMR timescale. As we observed with the short MYC[55–68] analogues, spectra from the unmodified MYC[1–84] and the T58-GlcNAc analogue showed comparable levels of Bin1 binding, indicated by similarly strong Bin1(SH3) CSPs. However, both MYC[1–84] and MYC[1–84]T58-GlcNAc gave smaller Bin1(SH3) CSPs than their corresponding MYC[55–68] counterparts, suggesting the longer, non-phosphorylated, fragments have less affinity for Bin1 compared to the corresponding short fragments. The reported *K*_D_ values, 33 μM for MYC[1–88],^31^ and 4.2 μM for MYC[55–68],^32^ support this observation. However, the same is not true for the phosphorylated MYC derivatives. All three phosphorylated MYC[1–84] analogues displayed larger Bin1 CSPs compared to the corresponding MYC[55–68] peptides (Fig. 4E). This suggests that Bin1(SH3) may have additional binding interactions with the longer MYC fragments, in agreement with the literature (Fig. 4F),^31^ or that auto-inhibitory effects are absent in the phosphorylated MYC[1–84] variants (as opposed to the non-phosphorylated forms, *vide infra*).

Across all MYC analogues screened, MYC[1–84]pT58 displayed the greatest CSPs (Fig. 4D and E). Furthermore, MYC[1–84]pT58 induced additional Bin1(SH3) CSPs at residues that were unaffected in the interaction with unmodified MYC[1–84] (*e.g.* N60, K63, L65, E66) (Fig. 4C and D). Close inspection of these Bin1 residues in the presence of the corresponding MYC[55–68] peptides (Fig. 3B) also shows some CSPs for Bin1-K63, L65, and E66 when MYC-T58 is phosphorylated, but not with unmodified MYC[55–68].

These results demonstrate the impact of MYC sequence length and PTMs on the binding interaction with Bin1 (Fig. 4E) and indicate the presence of additional Bin1 binding site(s) in MYC[1–84], previously hypothesized by Penn *et al.* (2012).^31^ With the short MYC[55–68] analogues, phosphorylation at T58 showed a reduction in Bin1 CSPs (indicating reduced level of binding), whereas the longer sequence (MYC[1–84]pT58) resulted in increased Bin1 CSPs compared to the unmodified MYC[1–84]. Additionally, S62 phosphorylation was reasonably tolerated in the interaction of Bin1 with MYC[1–84], but not in the interaction with MYC[55–68]pS62. Given that the Bin1 binding pocket is negatively charged, it is possible that phosphorylation at S62 or T58 still reduces Bin1 interaction in this region (residues 55–68) on MYC[1–84], but may promote Bin1's interaction at a distal site, for example with the PxxP motif at residues 42–45, which has been previously suggested in the literature (Fig. 4F).^31^ Nonetheless, our observations support the possibility of an additional Bin1 binding site within MYC that is present in residues 1–84, but not in residues 55–68.

## Discussion

Through rapid optimization of reaction conditions, an AFPS protocol for the incorporation of various PTM-amino acid building blocks into peptides and proteins was developed. Using this protocol, peptides containing clusters of tightly packed PTMs were successfully prepared, including phosphorylation on neighboring residues—a long-standing synthetic challenge.^17^ To summarize, five biologically relevant PTMs (phosphorylation, methylation, acetylation, sulfation, and glycosylation) were incorporated into peptide sequences (14–22 AA, ten examples), affording the target compounds in high yield (up to 41%) with excellent purity (>95%). Sequences containing two, three, four, five or seven PTMs were also successfully produced, including a pentaphosphorylated 22-mer peptide (2% isolated yield over 45 steps, >95% purity). Our methodology was then applied in the synthesis of five MYC protein fragments (84 AA) containing phosphorylation at T58 and S62, or glycosylation (*O*-GlcNAc) at T58, each of which were afforded in good yield (0.5–2.4%, ∼170 steps) with high purity (>92%). In the past, batch-SPPS and native chemical ligation (NCL) have proven very successful in the synthesis of peptides and proteins containing PTMs,^13–16^ yet these methods can be time-consuming, laborious, and often require optimization of several individual steps. Our AFPS protocol addresses these long-standing challenges, as it is applicable to all sequences and building blocks tested without the need to tailor it for each sequence. However, new phosphorylated amino acid building blocks or on-resin phosphorylation strategies may be required to minimize reagent use. An evaluation of the time and resources required for AFPS compared to batch-SPPS can be found in ESI† Section 9. All peptides and protein fragments were synthesized by AFPS within a few hours of synthesis time (∼20 AA per hour), thereby opening the possibility of studying PTM crosstalk on MYC and other proteins in the future on a broader scale.

Short (14 AA) and long (84 AA) unlabeled MYC fragments showed different binding behavior to the tumor suppressor protein Bin1, as investigated through NMR and nMS experiments. In agreement with the literature,^32^ MYC[55–68] fragments showed a switch-like behavior, with phosphorylation at S62 significantly reducing the interaction with Bin1. Phosphorylation at T58 of MYC[55–68] also decreased Bin1 binding (to a lesser extent than S62 phosphorylation), although glycosylation at the same site was completely tolerated. As S62 is more deeply buried in the binding interface compared to T58 (Fig. 3C), and is directly facing Bin1 Glu-25, phosphorylation of S62 may be disadvantageous for both steric and electrostatic reasons. While experiments with longer fragments followed the same trend, the binding interactions were more nuanced: MYC[1–84]pT58 resulted in the greatest Bin1 CSPs across all experiments with MYC[1–84] analogues, and the long MYC fragments containing phosphoserine (MYC[1–84]pS62 and MYC[1–84]pT58,pS62) showed increased Bin1 CSPs compared to their short peptide counterparts (MYC[55–68]pS62 and MYC[55–68]pT58,pS62). These findings may be explained by either, (a) an alternative binding mode of Bin1 with the phosphorylated MYC[1–84] analogues, or (b) an additional Bin1 binding site within MYC[1–84], that is absent in MYC[55–68]. The latter has been reported previously by Penn *et. al.* (2012), who suggested the motif at MYC residues 42–45 (PPAP, Fig. 4F) as another binding site for Bin1 and that the MYC:Bin1 complex exists in a dynamic and transient state.^31^ We therefore also speculate that the second PxxP motif around P42 of MYC[1–84] binds to the same Bin1 pocket, albeit with much weaker affinity.^31^ In this case, the additional CSPs in Bin1 around Asn-60 and Glu-66 observed with MYC[1–84]pT58 may therefore stem from an interaction of the MYC[1–84]pT58 phosphate group with Lys-63, -64, or -67 of Bin1, or from allosteric changes triggered by binding.

We also observed that all three phosphorylated MYC[1–84] constructs displayed increased Bin1(SH3) CSPs compared to their MYC[55–68] counterparts, while the unmodified MYC[1–84] and MYC[1–84]T58-GlcNAc gave smaller CSPs than MYC[55–68] and MYC[55–68]T58-GlcNAc, respectively (Fig. 4F). Notably, in MYC[1–84] nearly all positive charges are found in the C-terminal segment (residues 51–84) and all negative charges in the N-terminal segment (residues 12–48). This may result in electrostatic interactions^49^—either intra- or intermolecularly—and may therefore occlude the binding interface with Bin1. This autoinhibitory interaction would be absent in the shorter MYC[55–68] peptides, hence the greater affinity of MYC[55–68] for Bin1 compared to longer MYC fragments.^31^ Phosphorylation in the positively charged C-terminus of MYC[1–84] (*e.g.* on T58) may disrupt these MYC:MYC electrostatic interactions, exposing MBI and resulting in increased MYC:Bin1 binding, as observed with MYC[1–84]pT58. However, additional studies will be required in the future to support these statements. Using synthetic PTM-decorated MYC derivatives, these experiments demonstrate that, while short peptides are useful tools to study general PPI trends, longer MYC fragments or full-length MYC may be required to obtain a complete understanding of these PPIs.

## Conclusions

In conclusion, our AFPS methods for the production of PTM-peptides and proteins in high yield and purity enabled the study of the MYC:Bin1 complex and it's PTM-dependent behavior. The incorporation of multiple phosphorylated residues can be a particular challenge using traditional SPPS methods due to significant side-product formation. Through rapid optimization of AFPS methodology, side-reactions (*e.g.* β-elimination) were successfully mitigated, enabling the synthesis of peptides with phosphorylation at up to five residues in good overall yield (2–41%). Using these protocols, a series of phosphorylated and glycosylated analogues of MYC[55–68] and MYC[1–84] were prepared, and their interactions with Bin1(SH3) were analyzed using heteronuclear NMR and nMS. Overall, our results highlighted the influence of MYC fragment length on binding, exemplified by the contrasting effect of T58 phosphorylation on MYC[1–84] compared to MYC[55–68]. This work also supports previous reports^31^ regarding an additional, lower affinity, Bin1 binding site found within MYC[1–84] that is absent in MYC[55–68]. While new insights on PTM-modulation of MYC were gained, further efforts will be required to investigate the role of other neighboring phosphorylation sites and PTMs on MYC, as well as PTMs on Bin1. Additionally, MBI (MYC residues 45–68) is a hotspot for many other PPIs, and the PTM regulation of these PPIs is largely unknown. The MYC analogues generated in this study can therefore be applied to the biophysical analysis of other biologically relevant PPIs in future work. Many other intrinsically disordered proteins also carry PTM clusters that regulate PPIs, and thereby warrant in-depth investigations using synthetic PTM-containing analogues.^1,13^ In the future, our platform for the rapid synthesis of peptides and proteins with distinct PTM patterns will therefore enable the systematic study of these PTM functions and interactions at a molecular level.

## Data availability

Data for this paper, including raw NMR files, LCMS, and UHPLC data are available at Zenodo at https://doi.org/10.5281/zenodo.11173990

## Author contributions

The project was conceptualized by E. T. W. and N. H.; E. T. W. optimized the synthesis conditions for phosphorylated building blocks; experimental data on PTM-modified short MYC peptides was collected by E. T. W. and K. S.; experimental data on long MYC fragments was collected by E. T. W.; protein expression and protein NMR were performed by E. T. W. and M. S., the corresponding NMR data analysis and interpretation were carried out by E. T. W., M. S., O. Z and N. H.; native MS experiments and data interpretation were performed by I. M. M. A., M. D. V. and R. B.; the manuscript was written by E. T. W. and N. H. with input from all co-authors.

## Conflicts of interest

There are no conflicts to declare.

## Supplementary Material

SC-015-D4SC00481G-s001
