# Supplementary material for: Rapid flow-based synthesis of post-translationally modified peptides and proteins: a case study on MYC's transactivation domain
Source: Chem Sci. 2024 May 7;15(23):8756–65. doi: 10.1039/d4sc00481g (PMC11168107; doi:10.1039/d4sc00481g)
Supplement: SC-015-D4SC00481G-s001 [file SC-015-D4SC00481G-s001.pdf]

Electronic Supplementary Material (ESI) for Chemical Science.
This journal is © The Royal Society of Chemistry 2024

Supplementary Material

Rapid Flow-Based Synthesis of Post-Translationally Modified Peptides and Proteins: A Case Study on MYC

Authors: Elyse T. Williams,[a] Kevin Schiefelbein,[a] Matthias Schuster,[a] Ikhlas M. M. Ahmed,[b] Marije De Vries,[b] Rebecca Beveridge,[b] Oliver Zerbe,[a] Nina Hartrampf*[a]

*Correspondence to: nina.hartrampf@chem.uzh.ch

1 Table of Contents

1 Table of Contents 2

2 Materials 3

2.1 Reagents and solvents 3

3 General procedures 3

3.1 Automated flow-based peptide synthesis 3
3.2 Peptide cleavage and deprotection 4
3.3 Analytical Ultra-High Performance Liquid Chromatography (UHPLC) 4
3.4 Liquid Chromatography with High-Resolution Mass Spectrometry (LC-HRMS) 4
3.5 Semi-Preparative Reverse-Phase High Performance Liquid Chromatography (RP-HPLC) 5

4 Synthesis of PTM-modified peptides 6

4.1 Chemical Synthesis of unmodified test peptides 6
4.1.1 MYC[55–68] 6
4.1.2 MYC[55–68](E54W) 7
4.1.3 MYC[141–160] 9
4.1.4 MYC[55–76] 10
4.2 Optimization of ncAA coupling protocols 12
4.2.1 Optimized protocol for ncAA incorporation 13
4.3 Synthesis of PTM-modified test peptides 14
4.3.1 MYC[55–68](pT58, pS62, pS64, pS67) (four phosphorylations) 14
4.3.2 MYC[55–76](pT58, pS67, pS71, pS73, pY74) (five phosphorylations) 15
4.3.3 MYC[141–160](AcK148, sY152, AcK157) 16
4.3.4 MYC[141–160](Me$_3$K143, Me$_3$K148, pY152, AcK157) 17
4.3.5 MYC[141–160](Me$_3$K143, pS146, AcK148, pS151, sY152, AcK157, pS159 19
4.4 Synthesis of MYC[55–68] analogues for Bin1 binding 20
4.4.1 MYC[55–68](pT58) 20
4.4.2 MYC[55–68](pS62) 22
4.4.3 MYC[55–68](pT58, pS62) 23
4.4.4 MYC[55–68](GlcNAc-T58) 25
4.5 Synthesis of PTM-modified MYC[1–84] 26
4.5.1 MYC[1–84] 26
4.5.2 MYC[1–84](pS62) 28
4.5.3 MYC[1–84](pT58) 29
4.5.4 MYC[1–84](GlcNAc-T58) 31
4.5.5 MYC[1–84](pT58, pS62) 32

5 Bin1(SH3) protein expression and purification 34

6 Native Mass Spectrometry (nMS) 35

6.1 nMS: Bin1(SH3) and MYC[55–68] analogues 35

7 Protein NMR 36

7.1 Peptide and protein concentration determination for NMR experiments 36
7.2 NMR measurements 36

8 CSPs of Bin1(SH3) with MYC[1–84] analogues (scaled) 44

9 Comparison of AFPS to Batch-SPPS 44

10 References 45

2 Materials

2.1 Reagents and solvents

Fmoc- and side chain-protected L-amino acids (Fmoc-Ala-OH, Fmoc-Arg(Pbf)-OH, Fmoc-Asn(Trt)-OH, Fmoc-Asp(O*t*Bu)-OH, Fmoc-Cys(Trt)-OH, Fmoc-Gln(Trt)-OH, Fmoc-Glu(O*t*Bu)-OH, Fmoc-Gly-OH, Fmoc-His(Trt)-OH, Fmoc-Ile-OH, Fmoc-Leu-OH, Fmoc-Lys(Boc)-OH, Fmoc-Met-OH, Fmoc-Phe-OH, Fmoc-Pro-OH, Fmoc-Ser(*t*Bu)-OH, Fmoc-Thr(*t*Bu)-OH, Fmoc-Trp(Boc)-OH, Fmoc-Tyr(*t*Bu)-OH, Fmoc-Val-OH) were purchased from the Novabiochem-line from Sigma-Aldrich Canada Ltd.; *O*-(7-azabenzotriazol-1-yl)-*N,N,N',N'*-tetramethyluronium hexafluorophosphate (HATU), (7-azabenzotriazol-1-yloxy)tripyrrolidinophosphonium hexafluorophosphate (PyAOP), Fmoc-L-Ser(PO(OBzl)OH)-OH, Fmoc-L-Thr(PO(OBzl)OH)-OH, and Fmoc-L-Lys(Ac)-OH were purchased from Advanced ChemTech CreoSalus; (2*S*)-2-((((9*H*-Fluoren-9-yl)methoxy)carbonyl)amino)-3-(4-(((benzyloxy)(hydroxy)phosphoryl)oxy)phenyl)propanoic acid (Fmoc-L-Tyr(PO(OBzl)OH)-OH) was purchased from Fluorochem Ltd.; Fmoc-L-Lys(Me_3)-OH·Cl and Fmoc-L-Tyr(SO_3nP)-OH were purchased from Iris Biotech; Fmoc-L-Thr(GlcNAc(Ac_3)-ß-D)-OH was purchased from Bachem; *N*,*N*-diisopropylethylamine (*i*Pr_2NEt, DIPEA, 99.5%) was purchased from Sigma-Aldrich; trifluoroacetic acid (TFA, for HPLC, ≥99.0%), triisopropylsilane (TIPS, 98%) and 3,6-dioxa-1,8-octane-dithiol (DODT, 95%) were purchased from Sigma-Aldrich. *N,N*-Dimethylformamide (DMF) was purchased from the Supelco-line from Sigma-Aldrich Canada Ltd.; dichloromethane (DCM, ≥99.8%) was purchased from Fisher Scientific Ltd.; diethyl ether was purchased from Honeywell Riedel-de Haën; acetonitrile (MeCN; for HPLC, gradient grade, ≥99.9%) was purchased from Sigma-Aldrich. NovaPEG Rink Amide resin (0.41 or 0.20 mmol/g loading) was purchased from the Novabiochem-line from Sigma-Aldrich Canada Ltd.

3 General procedures

3.1 Automated flow-based peptide synthesis

Peptides were synthesized on an automated-flow system built in the Hartrampf lab, which is similar to the published automated fast-flow peptide synthesis (AFPS) system.[25] Capitalized letters refer to L-amino acids, lowercase letters refer to D-amino acids. Unless otherwise noted, the following settings were used for peptide synthesis: flow rate = 20 mL/min for coupling and deprotection steps, temperature = 90 °C (loop) and 85–90 °C (reactor). The standard synthetic cycle involves a first step of prewashing the resin at 90 °C for 60 s at 40 mL/min. During the coupling step, three HPLC pumps are used: a 50 mL/min pump head pumps the activating agent, a second 50 mL/min pump head pumps the amino acid, and a 5.0 mL/min pump head pumps DIPEA (*neat*). The 50 mL/min pump head pumps delivered 0.398679 mL of liquid per pump stroke, the 5.0 mL/min pump head pumps 0.039239 mL of liquid per pump stroke.

All peptides were prepared by AFPS on NovaPEG Rink Amide resin (0.41 or 0.20 mmol/g) and standard Fmoc/*t*Bu protected amino acids (0.40 M in DMF, 0.20 M final concentration) were coupled using HATU (0.38 M in DMF, 0.19 M final concentration) or PyAOP (0.38 M in DMF, 0.19 M final concentration) with DIPEA (delivered *neat*, approx. 0.27 M final concentration) at a

total flow rate of 20 mL/min. Amino acids A, D, E, F, G, I, K, L, M, P, S, W, and Y were coupled using HATU. Amino acids C, H, N, Q, R, T, and V were coupled using PyAOP. For amino acids D, E, F, G, I, K, L, M, P, S, W, and Y, a total volume of 6.4 mL of the "coupling solution" (i.e. amino acid (0.20 M), HATU or PyAOP (0.19 M), and DIPEA (0.27 M) in DMF) was applied for each coupling. For amino acids A, C, H, N, Q, R, S, T, and V, a total of 10.4 mL of "coupling solution" was applied for each coupling. All amino acids except C and H were preheated at 90 °C during the activation step with HATU or PyAOP, whereas C was preheated at 60 °C with PyAOP, and H was preactivated with PyAOP at room temperature. Removal of the N^{α}-Fmoc group was achieved using 20% piperidine with 1% formic acid in DMF at a flow rate of 20 mL/min and a total volume of 6.4 mL at 90 °C. Non-canonical amino acids (ncAAs) were incorporated using methods described in **Section 4.2.1**. Between each coupling and deprotection step, the resin was washed with DMF (32 mL) at 90 °C with a flow rate of 40 mL/min. After completion of the peptide sequence, the resins were manually washed with DCM (3 × 5 mL) and dried under reduced pressure.

3.2 Peptide cleavage and deprotection

All peptides were cleaved using a solution of TFA/TIPS/DODT/H_2O (94:1:2.5:2.5, *v*/*v*/*v*/*v*, 1.0–5.0 mL) for 2 h at room temperature with gentle mixing, unless otherwise stated. TFA was then removed by evaporation under a light stream of N_2, and the peptides were precipitated and isolated by centrifugation from ice-cold diethyl ether (14–45 mL), twice. The resulting peptide pellets were then briefly dried under a light stream of N_2, then dissolved in an aqueous solution containing 50% MeCN and lyophilized. Crude peptides were then analyzed by LC-HRMS and UHPLC. Pure peptide samples were obtained using RP-HPLC and were analyzed for purity by LC-HRMS and UHPLC.

3.3 Analytical Ultra-High Performance Liquid Chromatography (UHPLC)

For determination of purity by UHPLC, the filtered peptide solution was diluted in 10–50% acetonitrile (MeCN) in water with 0.1% TFA (500 µL) (except for phosphorylated peptides, which were dissolved in either: 10–50% acetonitrile (MeCN) in water without TFA, or in PBS buffer to a final concentration of approximately 1.0 mg/mL. The samples were analyzed on Agilent 1290 Infinity II Series using Agilent OpenLab CDS and ChemStation software.

For standard analysis of all peptide samples, analytical UHPLC spectra were recorded on an analytical Agilent Zorbax 300SB-C18 Narrow-Bore Rapid Resolution HD column (2.1 mm × 150 mm, 5.0 µm particle size) at a flow rate of 0.8 mL/min with UV detection at 214 nm. A binary solvent system was used, wherein Solvent A was 5% MeCN, 95% water with 0.1% TFA, and Solvent B was 95% MeCN containing 5% water and 0.1% TFA. After an isocratic period at 0% Solvent A for 3 min, a linear gradient of 0–100% Solvent B (corresponding to 5–95% MeCN) over 20 min was used (*ca.* 4.5% MeCN/min). The total method time was 23.1 min. Then, the column was re-equilibrated using a post-run method at 0% Solvent B for 2 min. Purities of the crude and purified peptides were determined by ChemStation integration of all UHPLC signals at 214 nm within Rt 3–18 min.

3.4 Liquid Chromatography with High-Resolution Electrospray Ionization Mass Spectrometry (LC-HRMS)

For determination of peptide masses and purity by LC-HRMS, the filtered peptide solution was diluted in 10–50% acetonitrile (MeCN) in water with 0.1% TFA (60–500 µL) (except for

phosphorylated and glycosylated peptides, which were dissolved in 10–50% MeCN in water, without TFA, or in PBS buffer to a final concentration of approximately 0.1 mg/mL). The samples were analyzed on an Acquity UPLC (Waters, Milford, USA) connected to an Acquity eλ diode array detector and a Synapt G2HR-ESI-QTOF-MS (Waters, Milford, USA).

For standard analysis of all peptide samples, LC-HRMS spectra were recorded on an Acquity BEH C8 HPLC column (2.1 × 100 mm, 1.7 μm particle size, Waters) kept at 30 °C at a flow rate of 0.4 mL/min with UV detection at 190–300 nm. A binary solvent system was used, wherein Solvent A was water containing 0.02% formic acid and 0.04% TFA, and Solvent B was MeCN containing 0.04% formic acid and 0.02% TFA. For samples containing PBS buffer or GnHCl, the first 3 min of the LC-HRMS method was diverted to the waste after passing through the column, to prevent salt contamination of the MS. All peptide samples were measured using **LC-HRMS Gradient A**, unless otherwise specified.

> **LC-HRMS Gradient A**: Isocratic at 10% Solvent B for 3 min, then linear gradient of 10–70% Solvent B over 9 min, followed by isocratic at 70% for 1 min.
>
> **LC-HRMS Gradient B**: Isocratic at 3% Solvent B for 3 min, then linear gradient of 3–95% Solvent B over 9 min, followed by isocratic at 95% Solvent B for 1 min.

UV spectra recorded at 1.2 nm resolution and 20 points s^{-1}; ESI: positive ionization mode (unless stated otherwise), capillary voltage 3.0 kV, sampling cone 40V, extraction cone 4V, N_2 cone gas 4 L h^{-1}, N_2 desolvation gas 800 L min^{-1}, source temperature 120 °C; mass analyzer in resolution mode: mass range 150–3000 *m*/*z* with a scan rate of 1 Hz; mass calibration to <2 ppm within 50–2500 *m*/*z* with a 5.0 mM aq. soln. of HCO_2Na, lock masses: *m*/*z* 195.0882 (caffein, 0.7 ng mL^{-1}) and 556.2771 (Leucine-enkephalin, 2 ng mL^{-1}).

All mass spectra show deconvoluted masses from the raw *m*/*z* values, calculated using Mestrelab Research S.L.© MestReNova v. 14.1 Mnova MS Suite. Purity based on LC-HRMS was calculated by calculating the Area Under the Curve (AUC) of desired product peak as a percentage of the AUC of all peaks (within 2–9 min) of the absorbance chromatogram (λ = 214 nm).

3.5 Semi-Preparative Reverse-Phase High Performance Liquid Chromatography (RP-HPLC)

Semi-preparative RP-HPLC was performed on a Shimadzu prominence HPLC system (Shimadzu Corp., Japan) with a CBM-40 system controller module, an FRC-10A fraction collector, two LC-20AR pumps, and an SPD-40 UV/VIS detector, using an Agilent Zorbax 300SB-C18 Semi-Preparative column (9.4 × 250 mm, 5 μm particle size) at a flow rate of 3.5 mL/min. A binary solvent system was used, wherein Solvent A was H_2O containing 0.1% TFA, and Solvent B was MeCN containing 0.1% TFA. Purifications were executed using the gradients specified in each procedure.

4 Synthesis of PTM-modified peptides

4.1 Chemical Synthesis of unmodified test peptides

4.1.1 MYC[55–68]

```
55      61
LLPTPP  LSPSRRSG-NH2  (14 AA)
```

The peptide MYC[55–68] was prepared via AFPS using Novabiochem® NovaPEG Rink Amide resin (0.41 mmol/g, 0.11 g, 45 µmol) using methods outlined in **General Procedure 3.1**. Total synthesis time to afford resin-bound MYC[55–68] (0.17 g) was approximately 0.5 h. Cleavage of the peptidyl-resin (78 mg) following **General Procedure 3.2** afforded the crude peptide (approx. 88% crude purity by UHPLC, monoisotopic mass calc. 1475.8572, found 1475.8539), **Figure S1**, which was then purified by semi-prep RP-HPLC following **General Procedure 3.5** with a gradient of 5–60%B over 55 min (*ca.* 1%B/min). Fractions were analyzed by LC-HRMS and UHPLC, combined, and lyophilized to afford MYC[55–68] (11 mg, >95% purity by UHPLC, 39% overall yield, monoisotopic mass calc. 1475.8572, found 1475.8538), **Figure S2**.

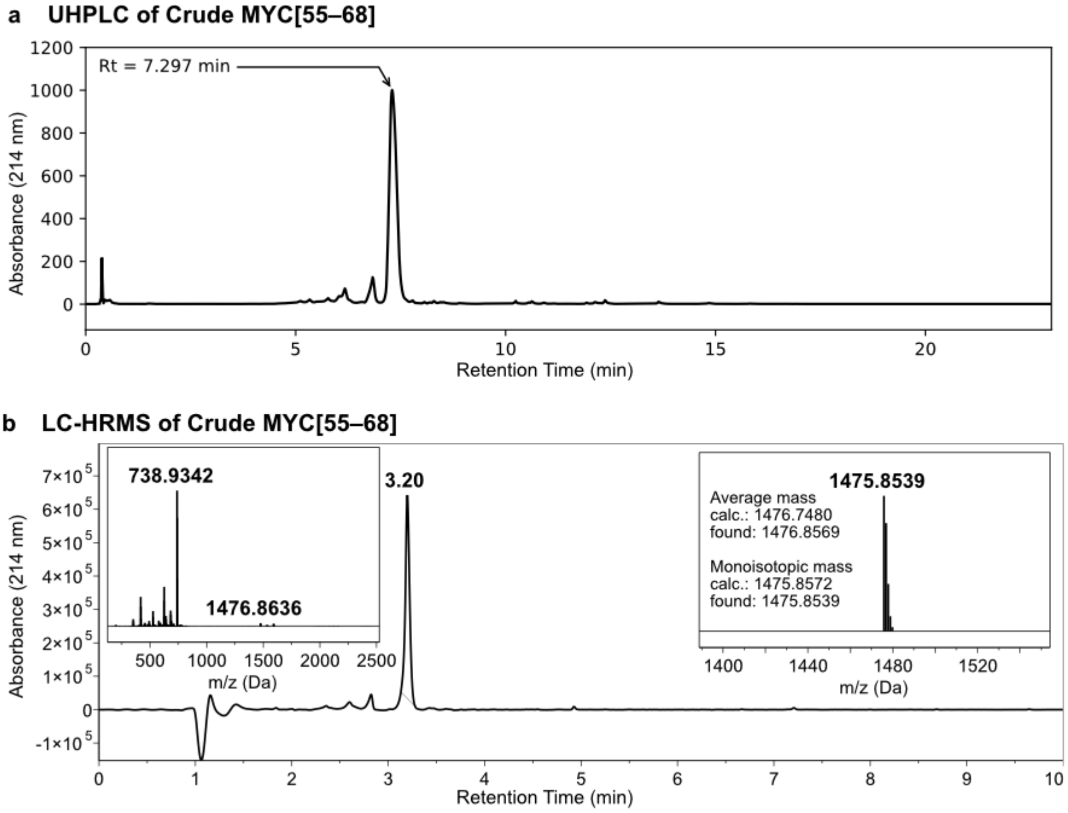

Figure S1. (a) UHPLC profile of crude MYC[55–68]; Rt = 7.297 min, 88% purity. Measured according to **General Procedure 3.3**. (b) LC-HRMS profile of crude MYC[55–68]; Rt = 3.20 min, measured according to **General Procedure 3.4**. Insert (left) = ESI-TOF spectrum. Insert (right) = deconvoluted mass spectra. Monoisotopic mass (ESI+) calc. for $C_{65}H_{113}N_{21}O_{18}$ 1475.8572, found 1475.8539.

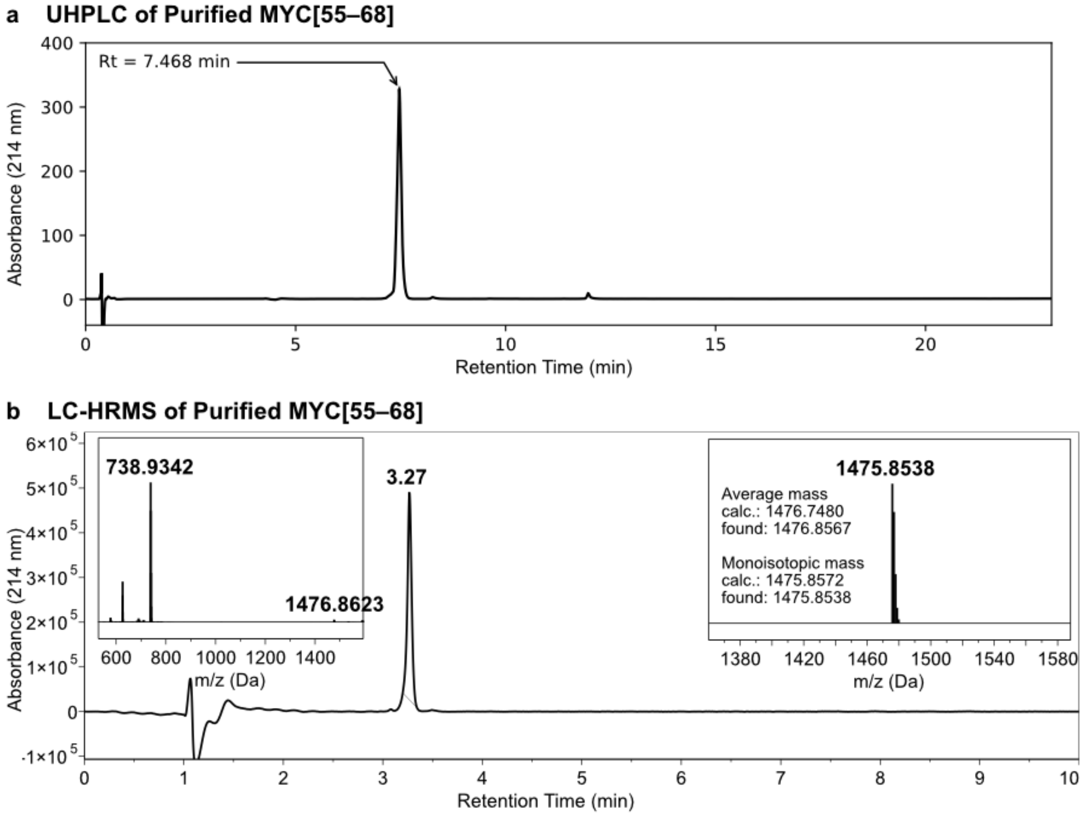

Figure S2. (a) UHPLC profile of purified MYC[55–68]; Rt = 7.468 min, >95% purity. Measured according to **General Procedure 3.3**. (b) LC-HRMS profile of purified MYC[55–68]; Rt = 3.27 min, measured according to **General Procedure 3.4**. Insert (left) = ESI-TOF spectrum. Insert (right) = deconvoluted mass spectra. Monoisotopic mass (ESI+) calc. for $C_{65}H_{113}N_{21}O_{18}$ 1475.8572, found 1475.8538.

4.1.2 MYC[55–68](E54W)

```
54       61
WLLPTPP  LSPSRRSG-NH2  (15 AA)
```

The peptide MYC[55–68](E54W) was prepared via AFPS using Novabiochem® NovaPEG Rink Amide resin (0.20 mmol/g, 0.15 g, 30 μmol) using methods outlined in **General Procedure 3.1**. Total synthesis time to afford resin-bound MYC[55–68](E54W) was approximately 0.5 h. Cleavage of the peptidyl-resin following **General Procedure 3.2** afforded the crude peptide (approx. 75% crude purity by UHPLC, monoisotopic mass calc. 1661.9366, found 1661.9414), **Figure S3**, which was then purified by semi-prep RP-HPLC following **General Procedure 3.5** with a gradient of 10–60%B over 50 min (*ca.* 1%B/min). Fractions were analyzed by LC-HRMS and UHPLC, combined, and lyophilized to afford MYC[55–68](E54W) (8.6 mg, >95% purity by UHPLC, 17% overall yield, monoisotopic mass calc. 1661.9366, found 1661.9372), **Figure S4**.

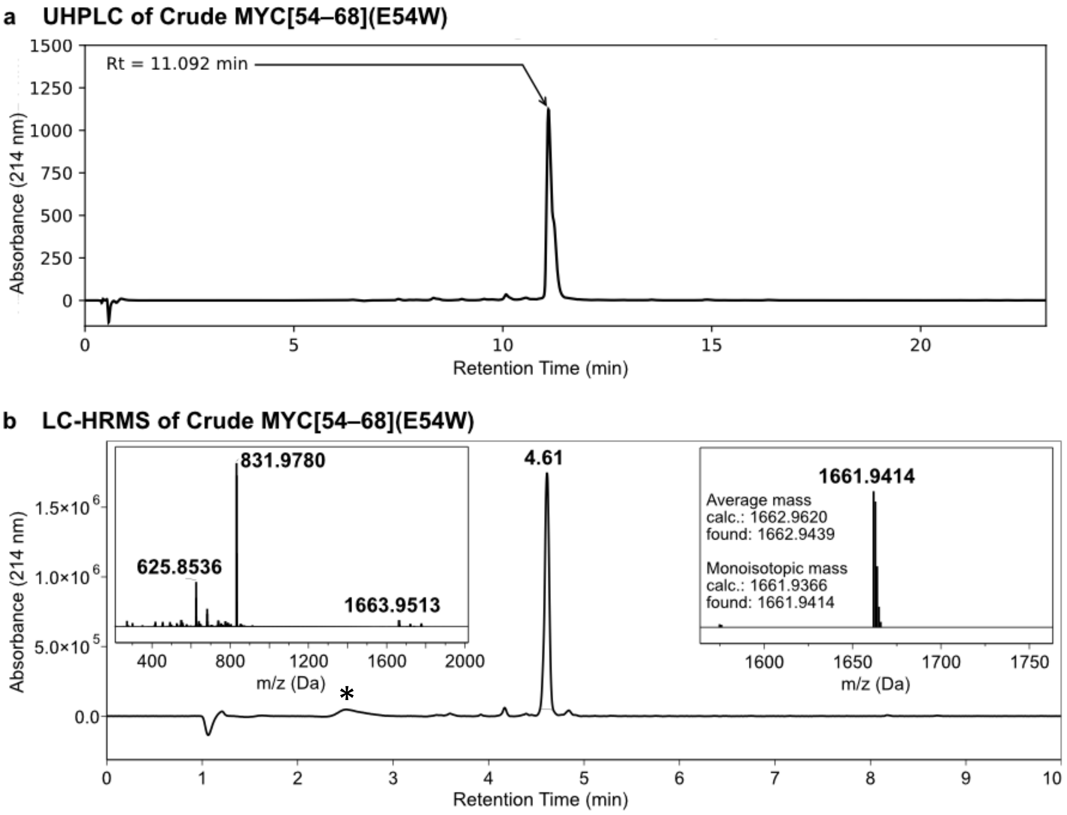

Figure S3. (a) UHPLC profile of crude MYC[55–68](E54W); Rt = 11.092 min, 75% purity. Measured according to **General Procedure 3.3**. (b) LC-HRMS profile of crude MYC[55–68](E54W); Rt = 4.61 min, measured according to **General Procedure 3.4**. *Solvent wave.[51] Insert (left) = ESI-TOF spectrum. Insert (right) = deconvoluted mass spectra. Monoisotopic mass (ESI+) calc. for $C_{76}H_{123}N_{23}O_{19}$ 1661.9366, found 1661.9414.

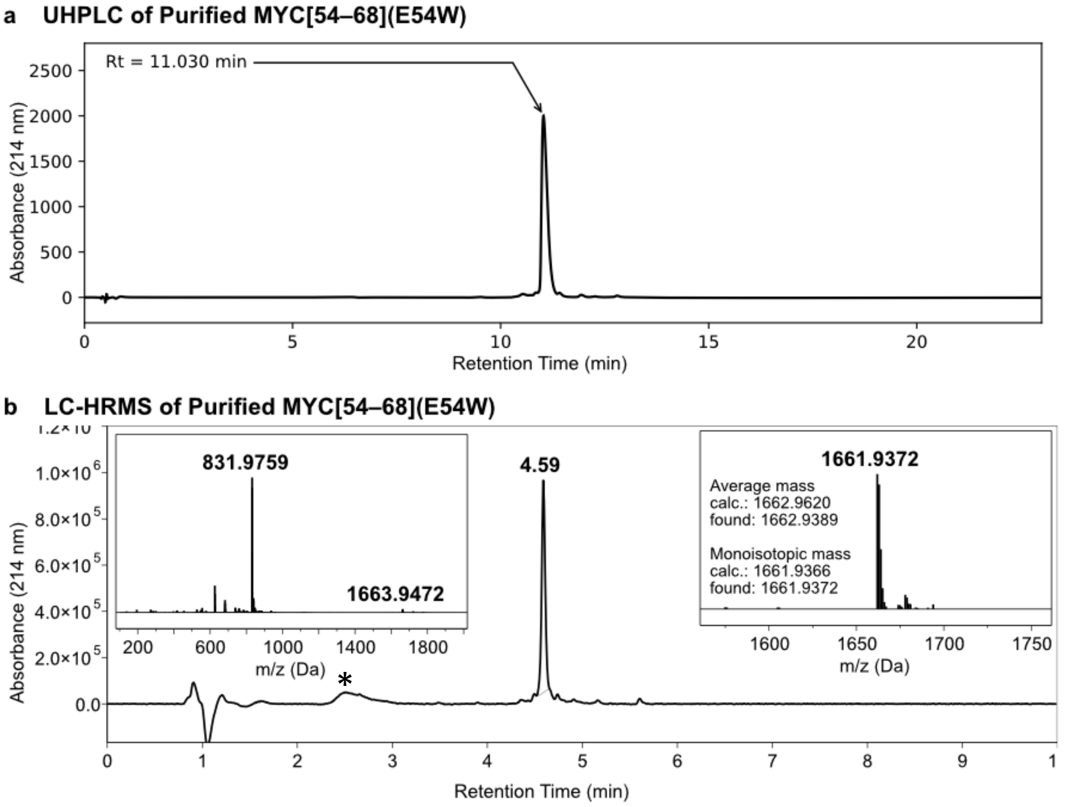

Figure S4. (a) UHPLC profile of purified MYC[55–68](E54W); Rt = 11.030 min, >95% purity. Measured according to **General Procedure 3.3**. (b) LC-HRMS profile of purified MYC[55–68](E54W); Rt = 4.59 min, measured according to **General Procedure 3.4**. *Solvent wave.[51] Insert (left) = ESI-TOF spectrum. Insert (right) = deconvoluted mass spectra. Monoisotopic mass (ESI+) calc. for $C_{76}H_{123}N_{23}O_{19}$ 1661.9366, found 1661.9372.

4.1.3 MYC[141–160]

```
141          151
AAKLVSEKLA   SYQAARKDSG-NH2 (20 AA)
```

The peptide MYC[141–160] was prepared via AFPS using Novabiochem® NovaPEG Rink Amide resin (0.20 mmol/g, 0.16 g, 32 μmol) using methods outlined in **General Procedure 3.1**. Total synthesis time to afford resin-bound MYC[141–160] was approximately 1 h. Cleavage of the peptidyl-resin following **General Procedure 3.2** afforded the crude peptide (79% crude purity by UHPLC, monoisotopic mass calc. 2091.1437, found 2091.1432), **Figure S5**, which was then purified by semi-prep RP-HPLC following **General Procedure 3.5** with a gradient of 10–60%B over 50 min (*ca.* 1%B/min). Fractions were analyzed by LC-HRMS and UHPLC, combined, and lyophilized to afford MYC[141–160] (10 mg, >95% purity by UHPLC, 15% overall yield, monoisotopic mass calc. 2091.1437, found 2091.1466), **Figure S6**.

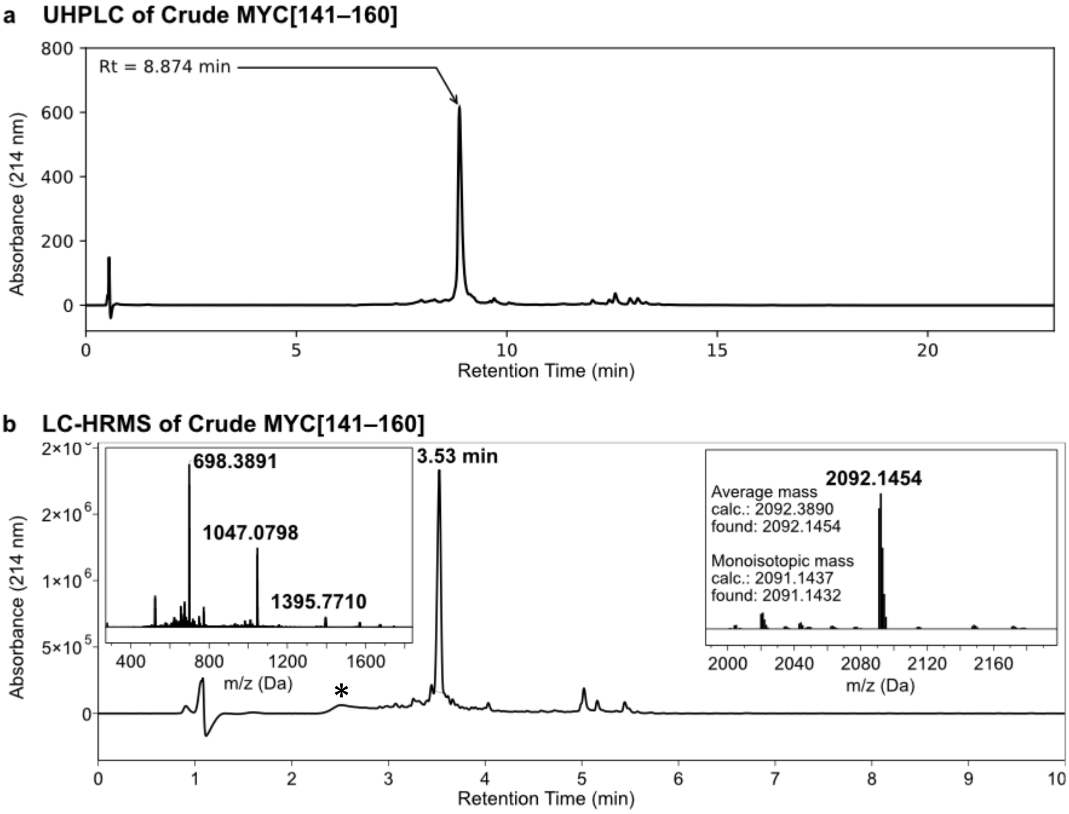

Figure S5. (a) UHPLC profile of crude MYC[141–160]; Rt = 8.874 min, 79% purity. Measured according to **General Procedure 3.3**. (b) LC-HRMS profile of crude MYC[141–160]; Rt = 3.53 min, measured according to **General Procedure 3.4**. *Solvent wave.[51] Insert (left) = ESI-TOF spectrum. Insert (right) = deconvoluted mass spectra. Monoisotopic mass (ESI+) calc. for $C_{90}H_{154}N_{28}O_{29}$ 2091.1437, found 2091.1432.

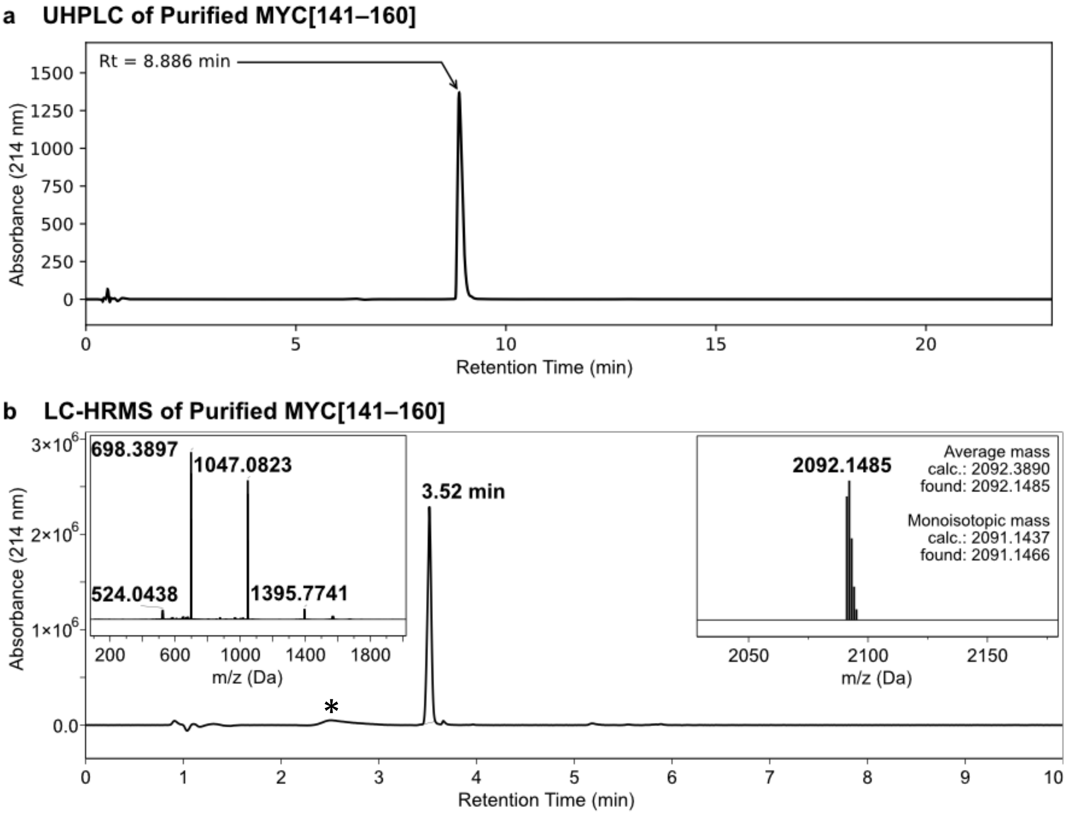

Figure S6. (a) UHPLC profile of purified MYC[141–160]; Rt = 8.886 min, >95% purity. Measured according to **General Procedure 3.3**. (b) LC-HRMS profile of purified MYC[141–160]; Rt = 3.52 min, measured according to **General Procedure 3.4** using **LC-HRMS Gradient B**. *Solvent wave.[51] Insert (left) = ESI-TOF spectrum. Insert (right) = deconvoluted mass spectra. Monoisotopic mass (ESI+) calc. for $C_{90}H_{154}N_{28}O_{29}$ 2091.1437, found 2091.1466.

4.1.4 MYC[55–76]

```
55        61            71
LLPTPP    LSPSRRSGLC    SPSYVA-NH2    (22 AA)
```

The peptide MYC[55–76] was prepared via AFPS using Novabiochem® NovaPEG Rink Amide resin (0.20 mmol/g, 0.15 g, 30 µmol) using methods outlined in **General Procedure 3.1**. Total synthesis time to afford resin-bound MYC[55–76] (0.21 g) was approximately 0.75 h. Cleavage of the peptidyl-resin (0.10 g) following **General Procedure 3.2** afforded the crude peptide (approx. 77% crude purity by UHPLC, monoisotopic mass calc. 2296.2362, found 2296.2390), **Figure S7**, which was then purified by semi-prep RP-HPLC following **General Procedure 3.5** with a gradient of 5–60%B over 55 min (*ca.* 1%B/min). Fractions were analyzed by LC-HRMS and UHPLC, combined, and lyophilized to afford MYC[55–76] (5.1 mg, >95% purity by UHPLC, 15% overall yield, monoisotopic mass calc. 2296.2362, found 2296.2370), **Figure S8**.

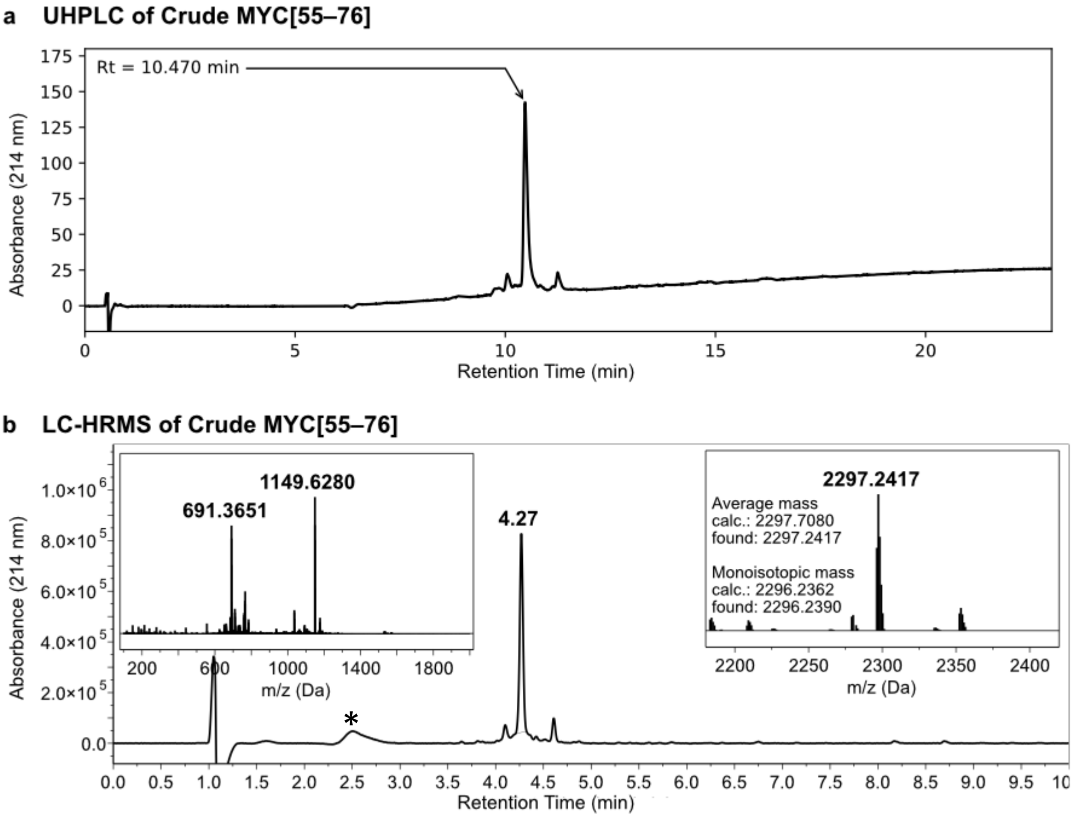

Figure S7. (a) UHPLC profile of crude MYC[55–76]; Rt = 10.470 min, 77% purity. Measured according to **General Procedure 3.3**. (b) LC-HRMS profile of crude MYC[55–76]; Rt = 4.27 min, measured according to **General Procedure 3.4**. *Solvent wave.[51] Insert (left) = ESI-TOF spectrum. Insert (right) = deconvoluted mass spectra. Monoisotopic mass (ESI+) calc. for $C_{102}H_{169}N_{29}O_{29}S$ 2296.2362, found 2296.2390.

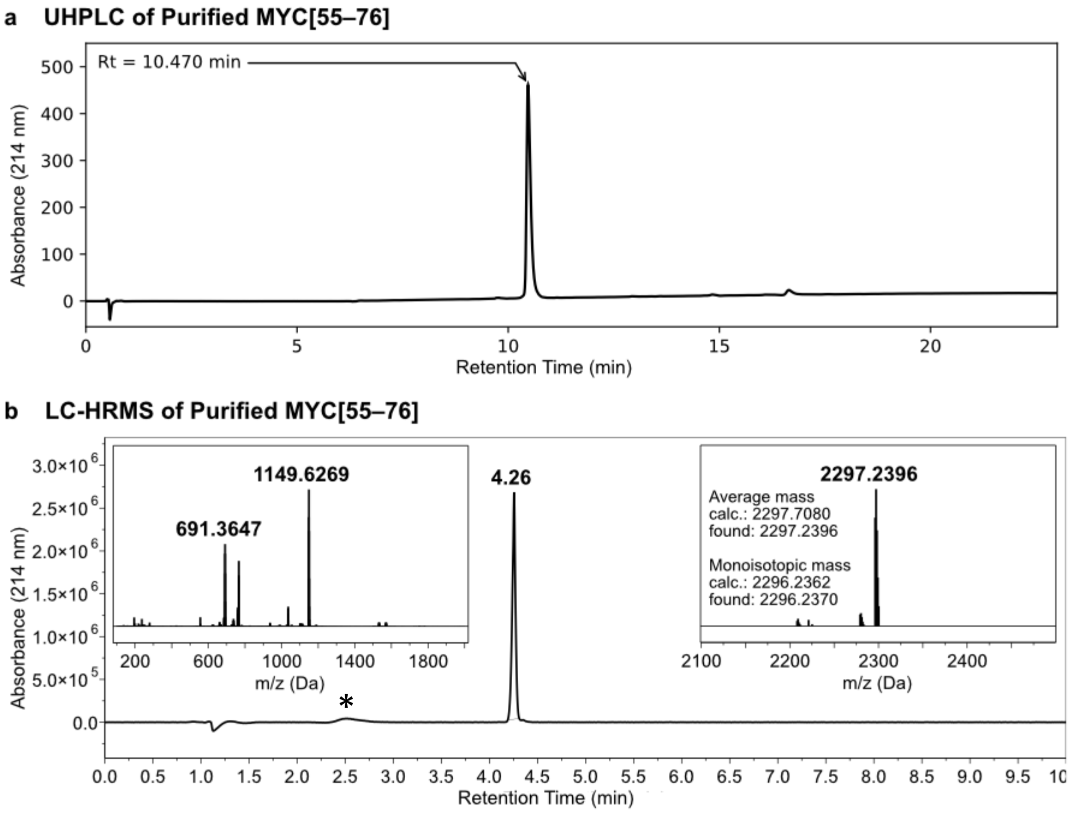

Figure S8. (a) UHPLC profile of purified MYC[55–76]; Rt = 10.470 min, >95% purity. Measured according to **General Procedure 3.3**. (b) LC-HRMS profile of purified MYC[55–76]; Rt = 4.26 min, measured according to **General Procedure 3.4**. *Solvent wave.[51] Insert (left) = ESI-TOF spectrum. Insert (right) = deconvoluted mass spectra. Monoisotopic mass (ESI+) calc. for $C_{102}H_{169}N_{29}O_{29}S$ 2296.2362, found 2296.2370.

4.2 Optimization of ncAA coupling protocols

MYC[63–84] was prepared by AFPS using Novabiochem® NovaPEG Rink Amide resin (0.20 mmol/g, 0.14 g, 28 μmol) using methods outlined in **General Procedure 3.1**. The resin was then split (approx. 5.0 μmol for each experiment) and used to investigate the coupling efficiency of Fmoc-Ser(PO(OBzl)OH)-OH (0.40 M in DMF) under different AFPS conditions (**Table S1**) (**Figure S9**). N^{α}-Fmoc deprotection was then carried out. Thereafter, Fmoc-L-Leu-OH was coupled and deprotected using AFPS methods outlined in **General Procedure 3.1**. The resulting peptidyl-resins were cleaved following **General Procedure 3.2** and analyzed by LC-HRMS following **General Procedure 3.4** (see **Figure S10** for LC-HRMS of **Table S1**, **Entries A** and **H**).

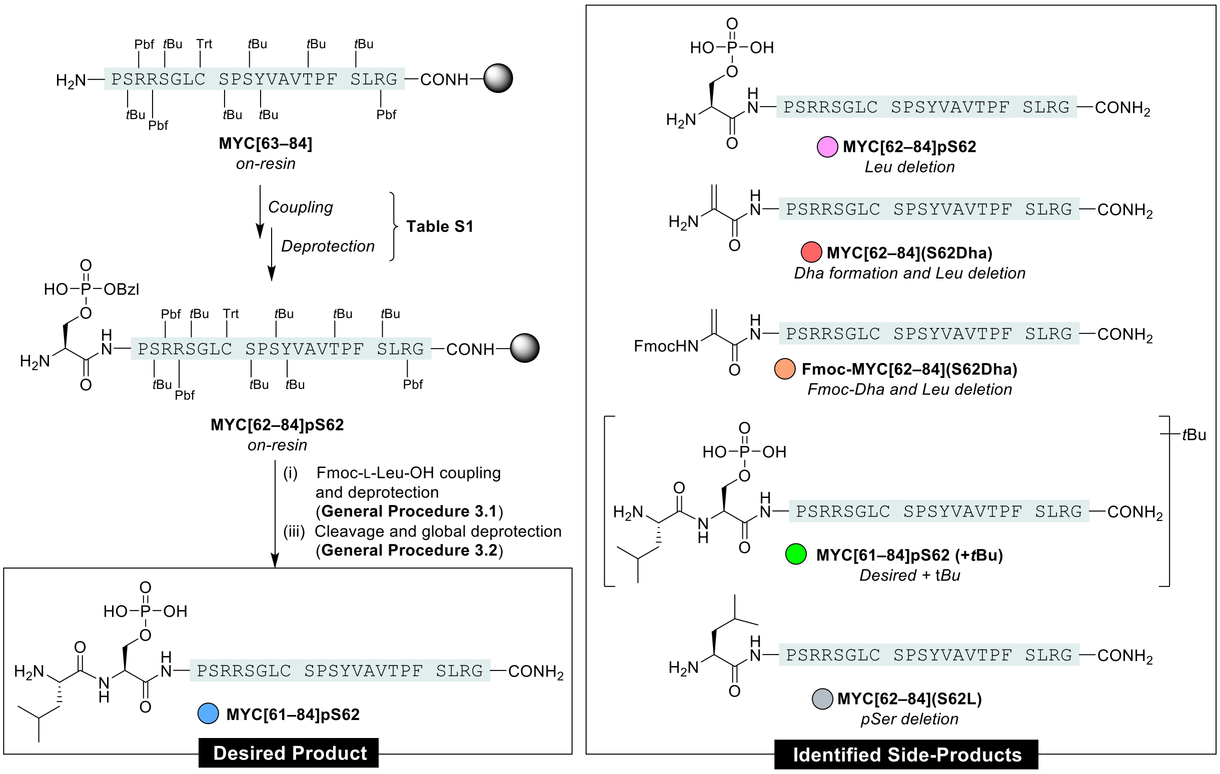

Figure S9. Scheme of MYC[61–84]pS62 synthesis and optimization of phosphoserine incorporation, including identified side-products. Colored circles match those shown in **Figure S10**.

Table S1. Investigated coupling and deprotection conditions for the incorporation of Fmoc-Ser(PO(OBzl)OH)-OH to afford MYC[61–84](pS62).

Entry	pSer Coupling Agent	Volume of ncAA+ (mL)	Overall Flow Rate (mL/min)	Pre-activation Temp. (°C)	Pre-activation Time (sec)	Resin Residence Time (sec)	Volume of Deprotection Solution# (mL)	Relative Ratio of LC-HRMS Peaks&			
								Desired %	Dha, Leu Deletion %	pSer Deletion %	Other %
**A**	PyAOP	1.2	5	60	6.45	36	2.4	66%	34%	*n.d.*	-
**B**	PyAOP	1.2	5	30	6.45	36	2.4	73%	25%	1%	1%
**C**	HATU	1.2	5	30	6.45	36	2.4	71%	28%	1%	-
**D**	PyAOP	1.2	10	30	3.23	18	2.4	84%	15%	1%	-
**E**	HATU	1.2	10	30	3.23	18	2.4	88%	10%	2%	-
**F**	PyAOP	1.2	20	30	1.61	9	2.4	88%	10%	2%	-
**G**	HATU	1.2	20	30	1.61	9	2.4	89%	8%	2%	1%
**H**	HATU	5.2	20/40*	30	1.61	9	6.4	98%	2%	*n.d.*	-

For **Entries A–G**, removal of the N^{α}-Fmoc group of the phosphorylated residue was achieved using 20% piperidine with 1% formic acid in DMF (2.4 mL). For **Entries B–H**, N^{α}-Fmoc deprotections were carried out with preheating at 30 °C. For **Entry A**, N^{α}-Fmoc deprotection was carried out with preheating at 60 °C. Overall Flow Rate indicates the flow rate used for coupling and deprotection of Fmoc-Ser(PO(OBzl)OH)-OH, and DMF washing steps. *For **Entry H**, couplings and deprotections were carried out at a flow rate of 20 mL/min, while washing steps were carried out at 40 mL/min. +ncAA = Fmoc-Ser(PO(OBzl)OH)-OH (0.40 M in DMF).

[#]Deprotection Solution = 20% piperidine and 1% formic acid in DMF. [&]Note: products related to the -*t*Bu adduct were not included in relative ratio of LC-HRMS peaks, as this adduct occurs during the TFA cleavage step and is not dependent on ncAA coupling conditions. *N.d.* = not detected.

The major products found in this experiment were the desired peptide MYC[61–84](pS62) (blue), Dha-containing peptide (red), MYC[61–84](pS62) with -*t*Bu adduct (green), MYC[62–84](pS62) (pink), and pSer deletion peptide. Coupling of Fmoc-L-Leu-OH onto the Dha-containing peptide was not observed, indicating low reactivity of the Dha-amine. The method of Entry **H** was found to give optimum results (**Figure S10b**).

a LC-HRMS of Crude MYC[61–84](pS62) (from Table S1, Entry A)

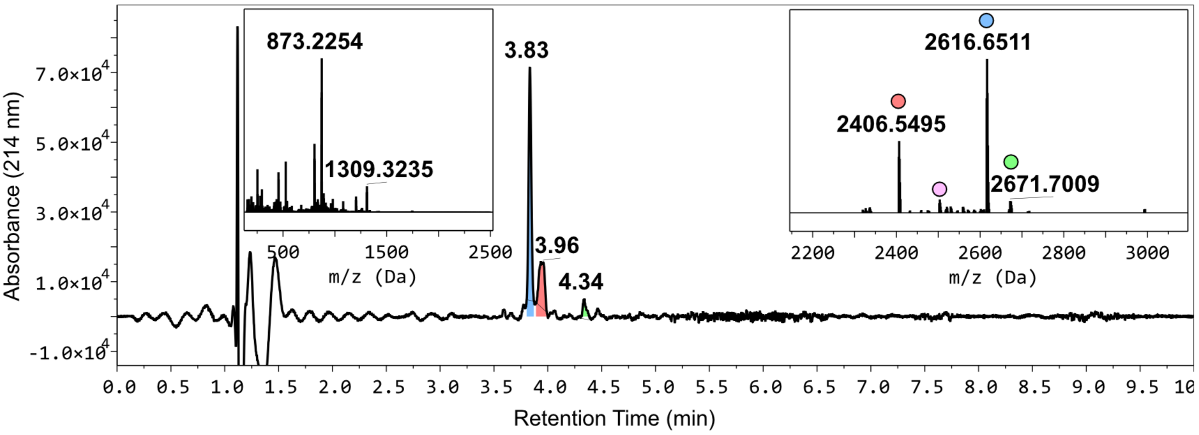

b LC-HRMS of Crude MYC[61–84](pS62) (from Table S1, Entry H)

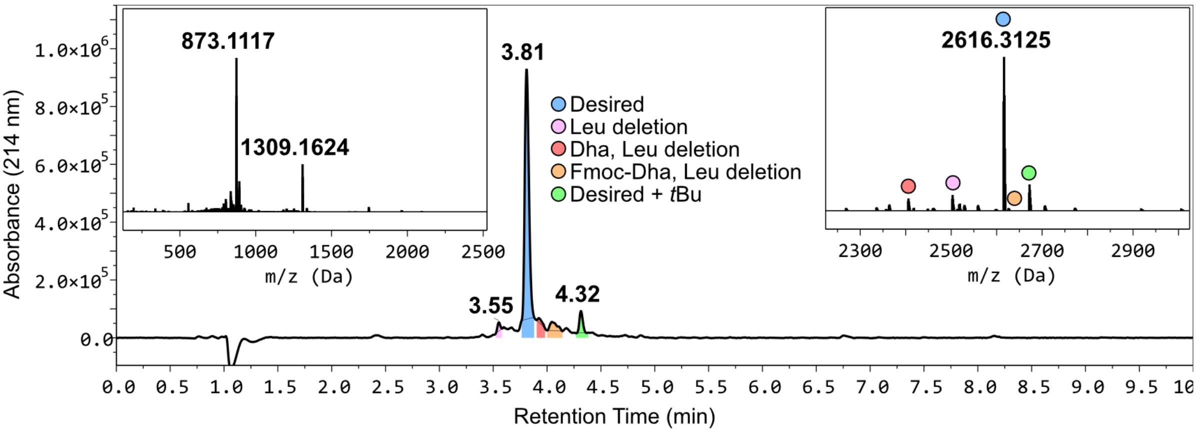

Figure S10. A) LC-HRMS profile of crude MYC[61–84](pS62) from **Table S1**, **Entry A**; Rt (desired) = 3.83 min, measured according to **General Procedure 3.4**. Insert (left) = ESI-TOF spectrum. Insert (right) = deconvoluted mass spectra. Monoisotopic mass (ESI+) calc. for $C_{111}H_{183}N_{34}O_{35}PS$ 2615.3043, found 2615.6485. B) LC-HRMS profile of crude MYC[61–84](pS62) from **Table S1**, **Entry H**; Rt (desired) = 3.81 min, measured according to **General Procedure 3.4**. Insert (left) = ESI-TOF spectrum. Insert (right) = deconvoluted mass spectra. Monoisotopic mass (ESI+) calc. for $C_{111}H_{183}N_{34}O_{35}PS$ 2615.3043, found 2615.3073.

4.2.1 Optimized protocol for ncAA incorporation

Non-canonical (phosphorylated, acetylated, sulfated, methylated) residues for all PTM-containing peptides were incorporated using AFPS as follows: Fmoc-protected ncAA (0.40 M in DMF) was coupled to the peptidyl-resin using HATU (0.38 M in DMF) with DIPEA (neat, 3.0 mL/min) at a total flow rate of 20 mL/min with preactivation at 30 °C. A total volume of 10.4 mL of the "coupling solution" (final composition: ncAA (0.20 M), HATU (0.19 M), and DIPEA in DMF) was applied for each coupling. Removal of the N^{α}-Fmoc group was achieved using 20% piperidine with 1% formic acid in DMF (preheating at 30 °C) at a flow rate of 20 mL/min and a total volume of 6.4 mL. Between each coupling and deprotection step, the resin was washed with DMF (32 mL) with a flow rate of 40 mL/min. Throughout the synthesis, the resin bed was kept at 85 °C.

4.3 Synthesis of PTM-modified test peptides

4.3.1 MYC[55–68](pT58, pS62, pS64, pS67) (four phosphorylations)

```
  55              61
   LLPpTPP   LpSPpSRRpSG-NH2   (14 AA)
```

The peptide MYC[55–68](pT58, pS62, pS64, pS67) was prepared via AFPS using Novabiochem® NovaPEG Rink Amide resin (0.20 mmol/g, 0.10 g, 20 µmol) using methods outlined in **General Procedure 3.1**. Total synthesis time to afford resin-bound MYC[55–68](pT58, pS62, pS64, pS67) was approximately 0.5 h. Cleavage of the peptidyl-resin following **General Procedure 3.2** afforded the crude peptide (approx. 64% crude purity by LC-HRMS, monoisotopic mass calc. 1795.7226, found 1795.7245), **Figure S11**, which was then purified by semi-prep RP-HPLC following **General Procedure 3.5** with a gradient of 10–60%B over 50 min (*ca.* 1%B/min). Fractions were analyzed by LC-HRMS and UHPLC, combined, and lyophilized to afford MYC[55–68](pT58, pS62, pS64, pS67) (5.6 mg, >95% purity by UHPLC, 8% overall yield, monoisotopic mass calc. 1795.7226, found 1795.7259), **Figure S12**.

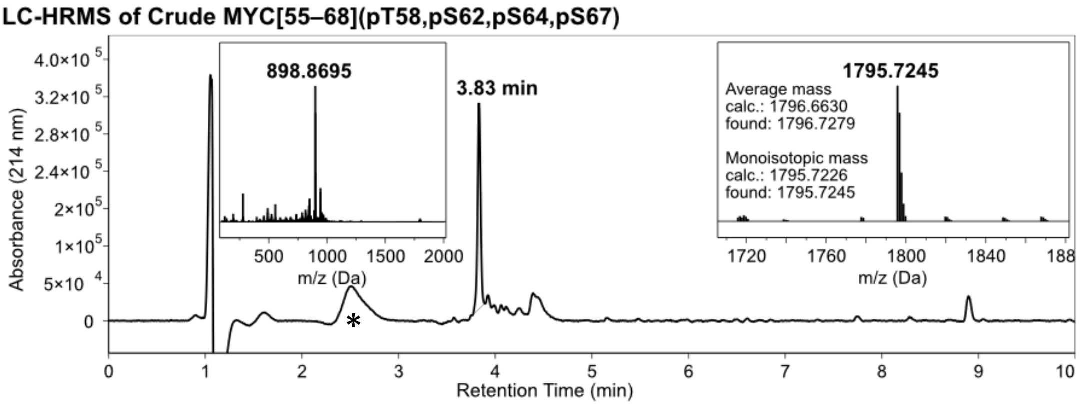

Figure S11. LC-HRMS profile of crude MYC[55–68](pT58,pS62,pS64,pS67); Rt = 3.83 min, measured according to **General Procedure 3.4**. Insert (left) = ESI-TOF spectrum. Insert (right) = deconvoluted mass spectra. Monoisotopic mass (ESI+) calc. for $C_{65}H_{117}N_{21}O_{30}P_4$ 1795.7226, found 1795.7245. *Solvent wave.[51] The UHPLC profile of crude MYC55-68(pT58, pS62, pS64, pS67) was unable to be obtained, likely due to adsorption on metal surfaces of the UHPLC instrument, which is known for (poly)phosphate-containing compounds.[53]

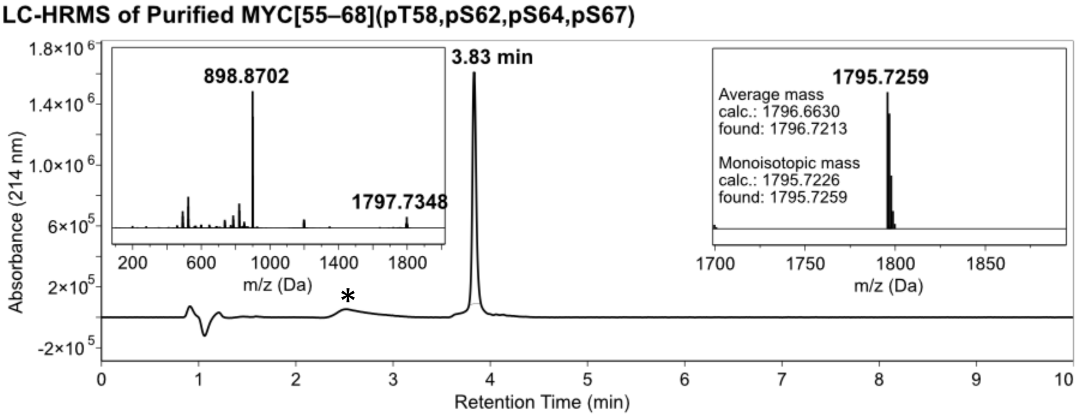

Figure S12. LC-HRMS profile of purified MYC[55–68](pT58,pS62,pS64,pS67); Rt = 3.83 min, measured according to **General Procedure 3.4**. Insert (left) = ESI-TOF spectrum. Insert (right) = deconvoluted mass spectra. Monoisotopic mass (ESI+) calc. for $C_{65}H_{117}N_{21}O_{30}P_4$ 1795.7226, found 1795.7259. *Solvent wave.[51] The UHPLC profile of purified MYC55-68(pT58, pS62, pS64, pS67) was unable to be obtained, likely due to adsorption on metal surfaces of the UHPLC instrument, which is known for (poly)phosphate-containing compounds.[53]

4.3.2 MYC[55–76](pT58, pS67, pS71, pS73, pY74) (five phosphorylations)

```
55             61              71
LLPpTPP  LSPSRRpSGLC  pSPpSpYVA-NH2    (22 AA)
```

The peptide MYC[55–76](pT58, pS67, pS71, pS73, pY74) was prepared via AFPS using Novabiochem® NovaPEG Rink Amide resin (0.20 mmol/g, 0.15 g, 30 μmol) using methods outlined in **General Procedure 3.1**. Total synthesis time to afford resin-bound MYC[55–76](pT58, pS67, pS71, pS73, pY74) (0.22 g) was approximately 0.75 h. Cleavage was performed following **General Procedure 3.2** with adaptation in cleavage solution: TFA/TIPS/EDT/H_2O (92.5:2.5:2.5:2.5, *v*/*v*/*v*/*v*, 2.5 mL). Cleavage of the peptidyl-resin (77 mg) afforded the crude peptide (approx. 54% crude purity by LC-HRMS, monoisotopic mass calc. 2696.0678, found 2696.0708), **Figure S13**, which was then purified by semi-prep RP-HPLC following **General Procedure 3.5** with a gradient of 5–60%B over 55 min (*ca.* 1%B/min). Fractions were analyzed by LC-HRMS and UHPLC, combined, and lyophilized to afford MYC[55–76](pT58, pS67, pS71, pS73, pY74) (0.5 mg, 95% purity by LC-HRMS, 2% overall yield, monoisotopic mass calc. 2696.0678, found 2696.0699), **Figure S14**.

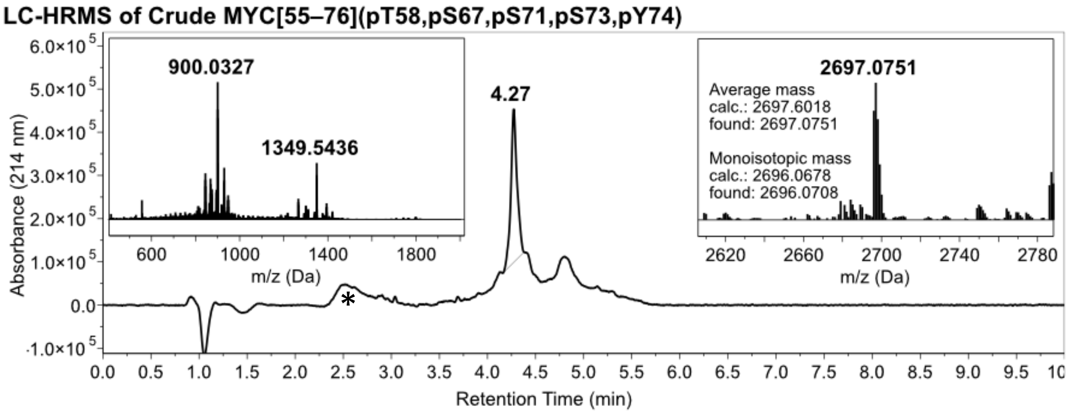

Figure S13. LC-HRMS profile of crude MYC[55–76](pT58,pS67,pS71,pS73,pY74); Rt = 4.27 min, 54% purity, measured according to **General Procedure 3.4**. Insert (left) = ESI-TOF spectrum. Insert (right) = deconvoluted mass spectra. Monoisotopic mass (ESI+) calc. for $C_{102}H_{174}N_{29}O_{44}P_5S$ 2696.0678, found 2696.0708. *Solvent wave.[51] The UHPLC profile of crude MYC55-76(pT58, pS67, pS71, pS73, pY74) was unable to be obtained, likely due to adsorption on metal surfaces of the UHPLC instrument, which is known for (poly)phosphate-containing compounds.[53]

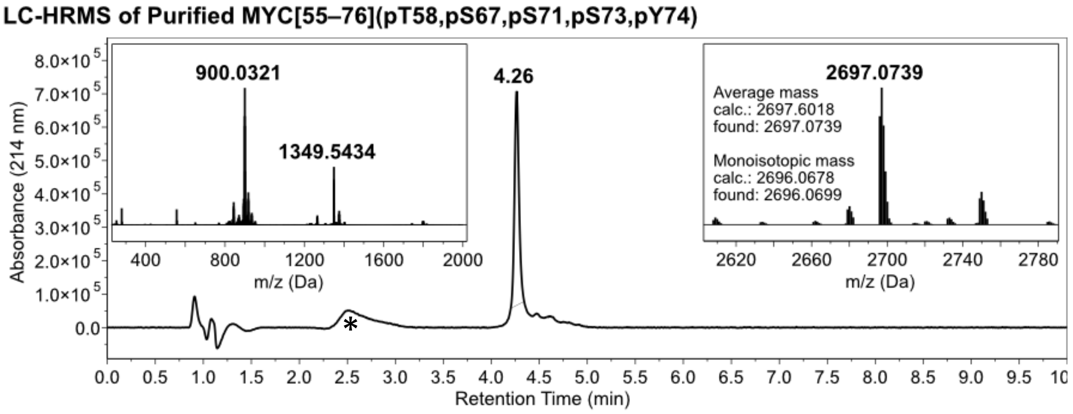

Figure S14. LC-HRMS profile of purified MYC[55–76](pT58,pS67,pS71,pS73,pY74); Rt = 4.26 min, 95% purity, measured according to **General Procedure 3.4**. Insert (left) = ESI-TOF spectrum. Insert (right) = deconvoluted mass spectra. Monoisotopic mass (ESI+) calc. for $C_{102}H_{174}N_{29}O_{44}P_5S$ 2696.0678, found 2696.0699. *Solvent wave.[51] The UHPLC profile of MYC55-76(pT58, pS67, pS71, pS73, pY74) was unable to be obtained, likely due to adsorption on metal surfaces of the UHPLC instrument, which is known for (poly)phosphate-containing compounds.[53]

4.3.3 MYC[141–160](AcK148, sY152, AcK157)

```
141          151
AA-AcK-LVSEKLA SsYQAAR-AcK-DSG-NH2 (20 AA)
```

The peptide MYC[141–160](AcK148, sY152, AcK157) was prepared via AFPS using Novabiochem® NovaPEG Rink Amide resin (0.20 mmol/g, 0.15 g, 30 µmol) using methods outlined in **General Procedure 3.1**. Total synthesis time to afford resin-bound MYC[141–160](AcK148, sY152, AcK157) (0.23 g) was approximately 1.25 h. Cleavage of the peptidyl-resin (0.12 g) following **General Procedure 3.2** afforded the crude nP-protected peptide. The crude peptide was dissolved in water (5.0 mL) overnight to deprotect the sulfate group yielding in crude unprotected peptide (approx. 73% crude purity by UHPLC, monoisotopic mass calc. 2255.1216, found 2255.1231), **Figure S15**, which was then purified by semi-prep RP-HPLC following **General Procedure 3.5** with a gradient of 5–60%B over 55 min (*ca.* 1%B/min). Fractions were analyzed by LC-HRMS and UHPLC, combined, and lyophilized to afford MYC[141–160](AcK148, sY152, AcK157) (7.1 mg, 90% purity by UHPLC, 20% overall yield, monoisotopic mass calc. 2255.1216, found 2255.2218), **Figure S16**.

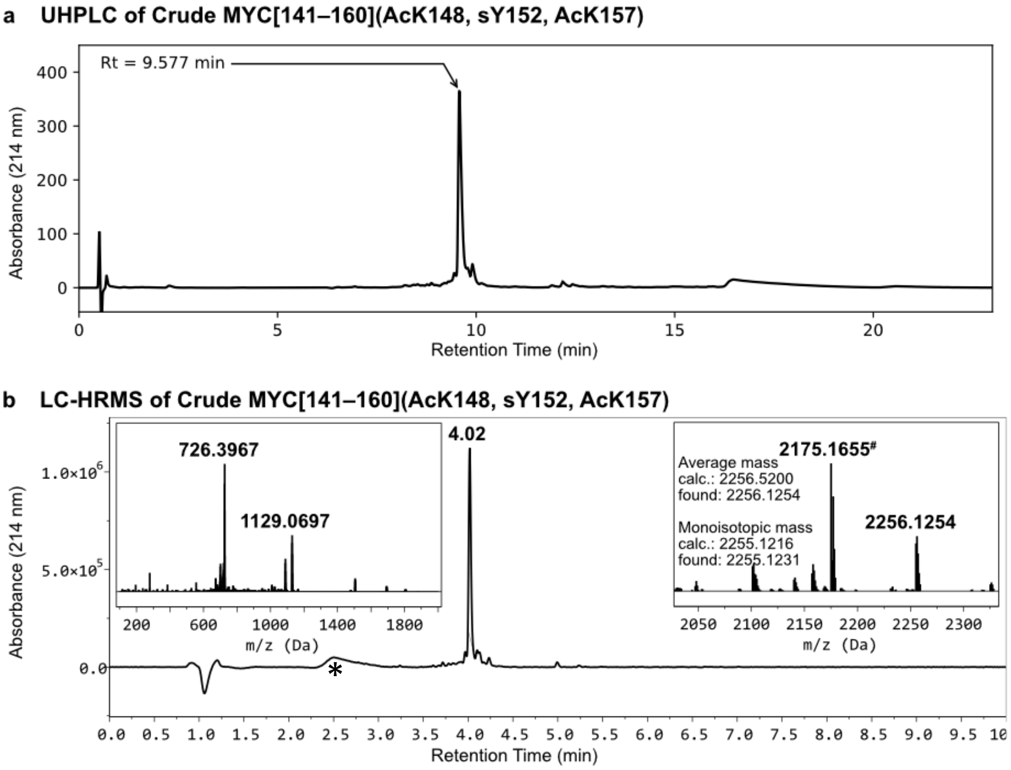

Figure S15. (a) UHPLC profile of crude MYC[141–160](AcK148,sY152,AcK157); Rt = 9.577 min, 73% purity. Measured according to **General Procedure 3.3**. (b) LC-HRMS profile of crude MYC[141–160](AcK148,sY152,AcK157); Rt = 4.02 min, measured in ESI positive ionization mode according to **General Procedure 3.4** using **LC-HRMS Gradient B**. *Solvent wave.[51] Insert (left) = ESI-TOF spectrum. Insert (right) = deconvoluted mass spectra. Monoisotopic mass (ESI+) calc. for $C_{94}H_{158}N_{28}O_{34}S$ 2255.1216, found 2255.1231. #Loss of sulfate in ESI positive ionization mode.

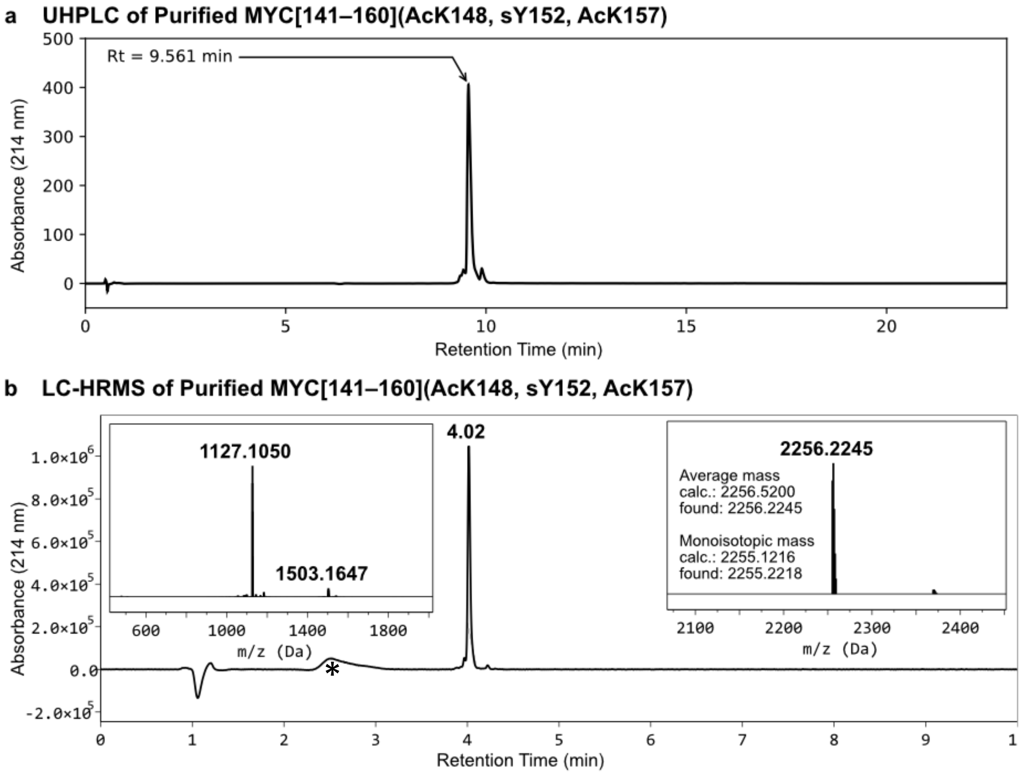

Figure S16. (a) UHPLC profile of purified MYC[141–160](AcK148,sY152,AcK157); Rt = 9.561 min, 90% purity. Measured according to **General Procedure 3.3**. (b) LC-HRMS profile of purified MYC[141–160](AcK148,sY152,AcK157); Rt = 4.02 min, measured in ESI positive ionization mode according to **General Procedure 3.4** using **LC-HRMS Gradient B**. *Solvent wave.[51] Insert (left) = ESI-TOF spectrum. Insert (right) = deconvoluted mass spectra. Monoisotopic mass (ESI-) calc. for $C_{94}H_{158}N_{28}O_{34}S$ 2255.1216, found 2255.1231.

4.3.4 MYC[141–160](Me$_3$K143, Me$_3$K148, pY152, AcK157)

```
141                           151
AA-Me3K-LVSE-Me3K-LA          SpYQAAR-AcK-DSG-NH2 (20 AA)
```

The peptide MYC[141–160](Me$_3$K143, Me$_3$K148, pY152, AcK157) was prepared via AFPS using Novabiochem® NovaPEG Rink Amide resin (0.20 mmol/g, 0.15 g, 30 µmol) using methods outlined in **General Procedure 3.1**. Total synthesis time to afford resin-bound MYC[141–160](Me$_3$K143, Me$_3$K148, pY152, AcK157) (0.19 g) was approximately 1.25 h. Cleavage of the peptidyl-resin (0.10 g) following **General Procedure 3.2** afforded the crude peptide (approx. 72% crude purity by UHPLC, monoisotopic mass calc. 2297.2144 [phosphate deprotonated], found 2297.2144), **Figure S17**, which was then purified by semi-prep RP-HPLC following **General Procedure 3.5** with a gradient of 5–60%B over 55 min (*ca.* 1%B/min). Fractions were analyzed by LC-HRMS and UHPLC, combined, and lyophilized to afford MYC[141–160](Me$_3$K143, Me$_3$K148, pY152, AcK157) (7.6 mg, >95% purity by UHPLC, 21% overall yield, monoisotopic mass calc. 2297.2144 [phosphate deprotonated], found 2297.2138), **Figure S18**.

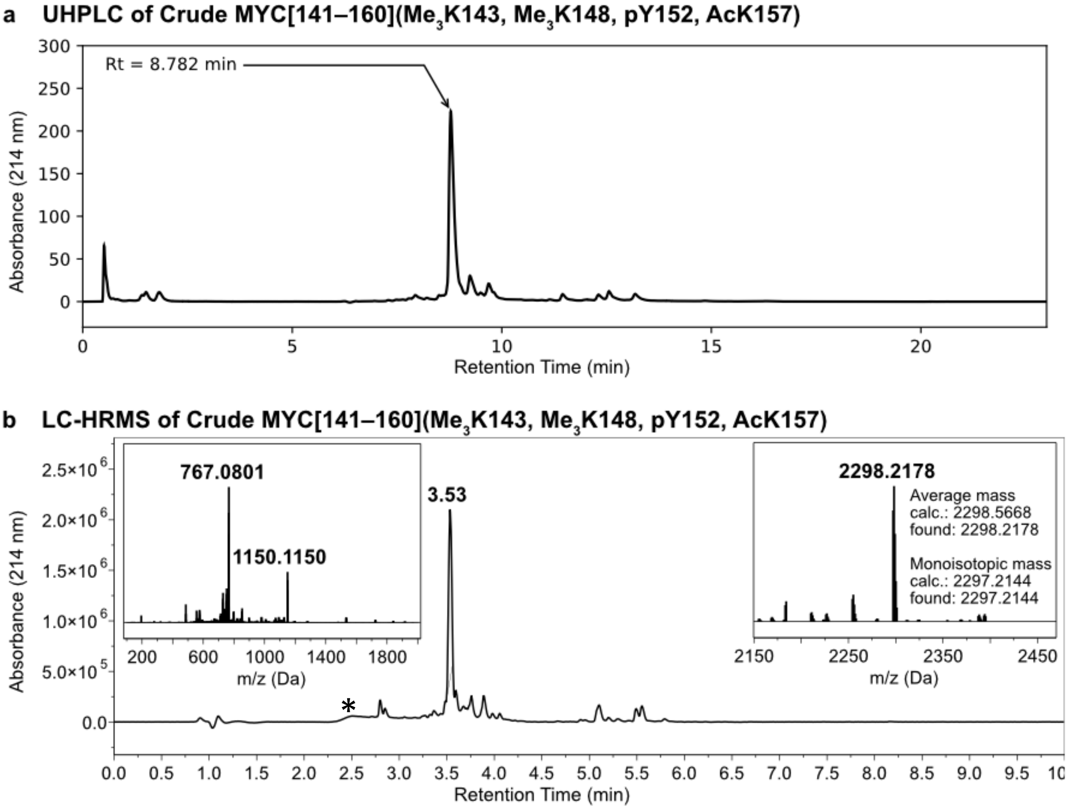

Figure S17. (a) UHPLC profile of crude MYC[141–160](Me_3K143,Me_3K148,pY152,AcK157); Rt = 8.782 min, 72% purity. Measured according to **General Procedure 3.3**. (b) LC-HRMS profile of crude MYC[141–160](Me_3K143,Me_3K148,pY152,AcK157); Rt = 3.53 min, measured according to **General Procedure 3.4** using **LC-HRMS Gradient B**. *Solvent wave.[51] Insert (left) = ESI-TOF spectrum. Insert (right) = deconvoluted mass spectra. Monoisotopic mass (ESI+) calc. for $C_{98}H_{169}N_{28}O_{33}P$ 2297.2144 (phosphate deprotonated), found 2297.2144.

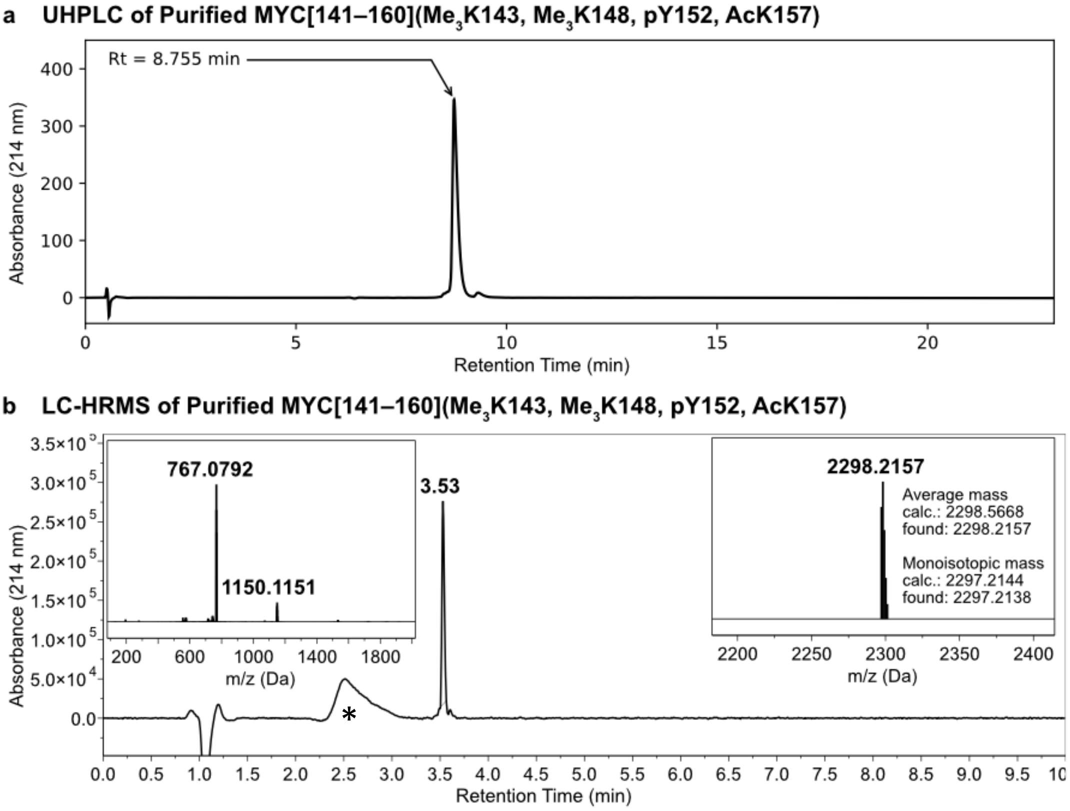

Figure S18. (a) UHPLC profile of purified MYC[141–160](Me_3K143,Me_3K148,pY152,AcK157); Rt = 8.755 min, >95% purity. Measured according to **General Procedure 3.3**. (b) LC-HRMS profile of purified MYC[141–160](Me_3K143,Me_3K148,pY152,AcK157); Rt = 3.53 min, measured according to **General Procedure 3.4** using **LC-HRMS Gradient B**. *Solvent wave.[51] Insert (left) = ESI-TOF spectrum. Insert (right) = deconvoluted mass spectra. Monoisotopic mass (ESI+) calc. for $C_{98}H_{169}N_{28}O_{33}P$ 2297.2144 (phosphate deprotonated), found 2297.2138.

4.3.5 MYC[141–160](Me₃K143, pS146, AcK148, pS151, sY152, AcK157, pS159

```
141                           151
AA-Me3K-LVpSE-AcK-LA          pSsYQAAR-AcK-DpSG-NH2 (20 AA)
```

The peptide MYC[141–160](Me$_3$K143, pS146, AcK148, pS151, sY152, AcK157, pS159) was prepared via AFPS using Novabiochem® NovaPEG Rink Amide resin (0.20 mmol/g, 0.15 g, 30 µmol) using methods outlined in **General Procedure 3.1**. Total synthesis time to afford resin-bound MYC[141–160](Me$_3$K143, pS146, AcK148, pS151, sY152, AcK157, pS159) (0.20 g) was approximately 1.25 h. Cleavage of the peptidyl-resin (0.10 g) following **General Procedure 3.2** afforded the crude nP-protected peptide. The crude peptide was dissolved in phosphate buffer (5.0 mL, 20 mM, pH 7.0) overnight to deprotect the sulfate group yielding in crude unprotected peptide (approx. 69% crude purity by UHPLC, monoisotopic mass calc. 2537.0675, found 2537.0729), **Figure S19**, which was then purified by semi-prep RP-HPLC following **General Procedure 3.5** with a gradient of 5–60%B over 55 min (*ca.* 1%B/min). Fractions were analyzed by LC-HRMS and UHPLC, combined, and lyophilized to afford MYC[141–160](Me$_3$K143, pS146, AcK148, pS151, sY152, AcK157, pS159) (4.3 mg, 95% purity by UHPLC, 12% overall yield, monoisotopic mass calc. 2537.0675, found 2537.1977), **Figure S20**.

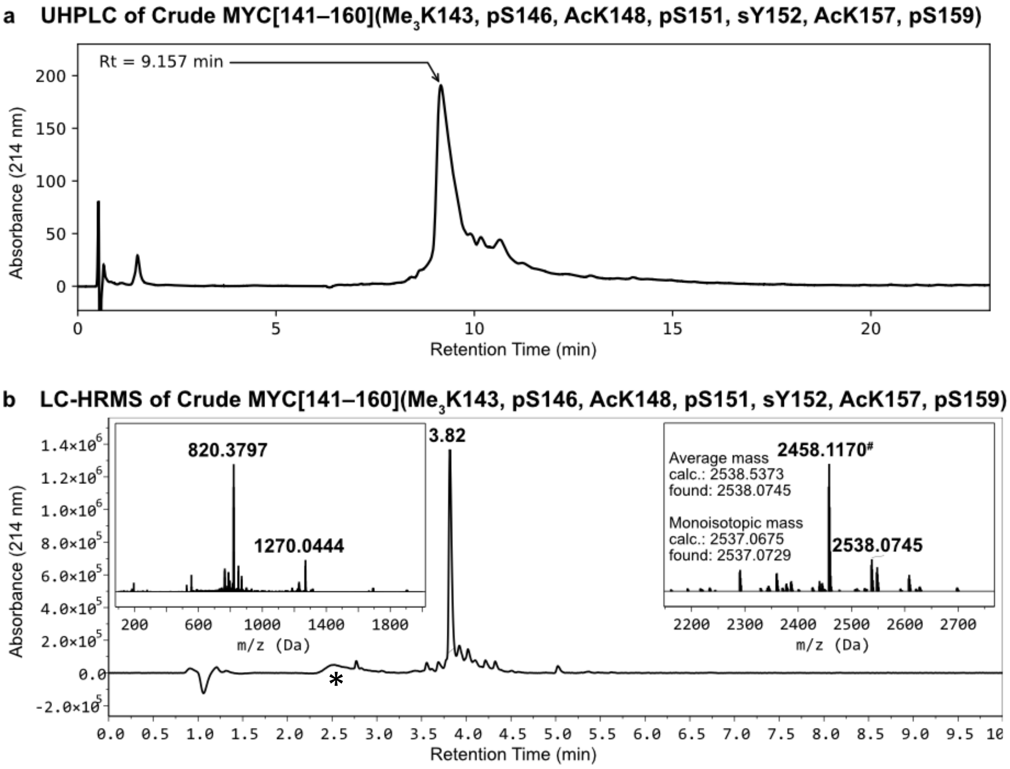

Figure S19. (a) UHPLC profile of crude MYC[141–160](Me$_3$K143,pS146,AcK148,pS151,sY152,AcK157,pS159); Rt = 9.157 min, 69% purity. Measured according to **General Procedure 3.3**. Peak broadening observed due to adsorption and aggregation on the column. (b) LC-HRMS profile of crude MYC[141–160](Me$_3$K143,pS146,AcK148,pS151,sY152,AcK157,pS159); Rt = 3.82 min, measured in ESI positive ionization mode according to **General Procedure 3.4** using **LC-HRMS Gradient B**. *Solvent wave.[51] Insert (left) = ESI-TOF spectrum. Insert (right) = deconvoluted mass spectra. Monoisotopic mass (ESI+) calc. for $C_{97}H_{167}N_{28}O_{43}P_3S$ 2537.0675, found 2537.0729. #Loss of sulfate in ESI positive ionization mode.

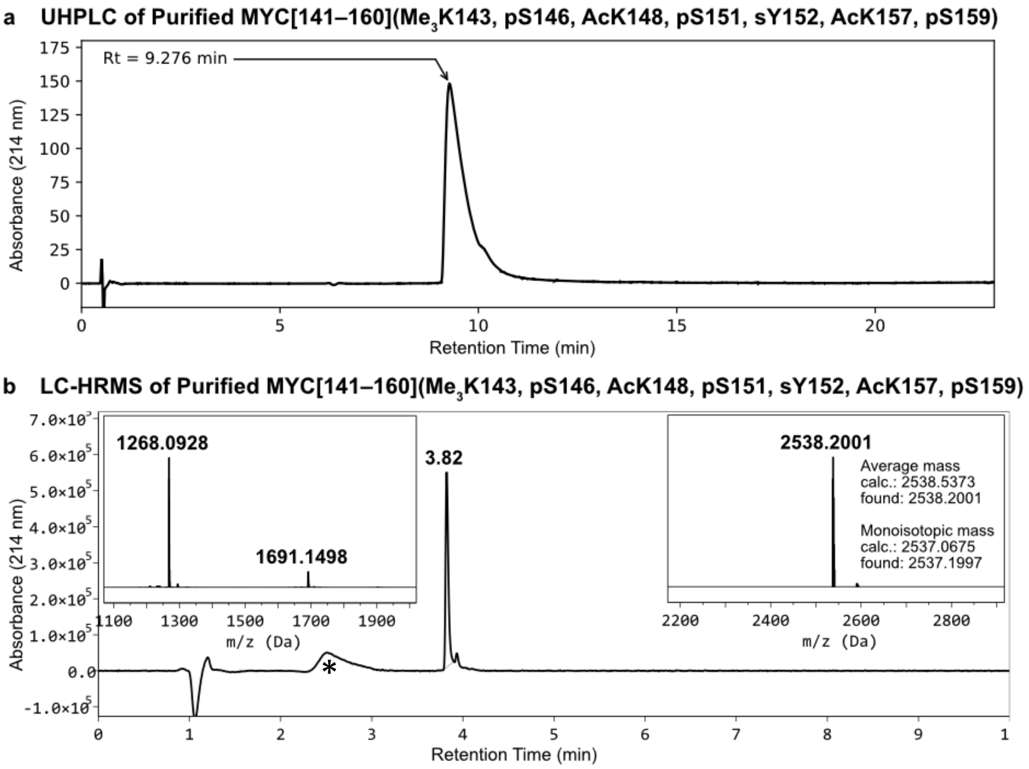

Figure S20. (a) UHPLC profile of purified MYC[141–160](Me_3K143,pS146,AcK148,pS151,sY152,AcK157,pS159); Rt = 9.276 min, 95% purity. Measured according to **General Procedure 3.3**. Peak broadening observed due to adsorption and aggregation on the column. (b) LC-HRMS profile of purified MYC[141–160](Me_3K143,pS146,AcK148,pS151,sY152,AcK157,pS159); Rt = 3.82 min, measured in ESI negative ionization mode according to **General Procedure 3.4** using **LC-HRMS Gradient B**. *Solvent wave.[51] Insert (left) = ESI-TOF spectrum. Insert (right) = deconvoluted mass spectra. Monoisotopic mass (ESI-) calc. for $C_{97}H_{167}N_{28}O_{43}P_3S$ 2537.0675, found 2537.1977.

4.4 Synthesis of MYC[55–68] analogues for Bin1 binding

4.4.1 MYC[55–68](pT58)

```
55      61
LLPpTPP LSPSRRSG-NH2  (14 AA)
```

The peptide MYC[55–68](pT58) was prepared via AFPS using Novabiochem® NovaPEG Rink Amide resin (0.41 mmol/g, 0.11 g, 45 µmol) using methods outlined in **General Procedure 3.1**. Total synthesis time to afford resin-bound MYC[55–68](pT58) (0.19 g) was approximately 0.5 h. Cleavage of the peptidyl-resin (93 mg) following **General Procedure 3.2** afforded the crude peptide (approx. 66% crude purity by UHPLC, monoisotopic mass calc. 1555.8236, found 1555.8264), **Figure S22**, which was then purified by semi-prep RP-HPLC following **General Procedure 3.5** with a gradient of 5–60%B over 55 min (*ca.* 1%B/min). Fractions were analyzed by LC-HRMS and UHPLC, combined, and lyophilized to afford MYC[55–68](pT58) (14 mg, >95% purity by UHPLC, 41% overall yield, monoisotopic mass calc. 1555.8236, found 1555.8258), **Figure S22**.

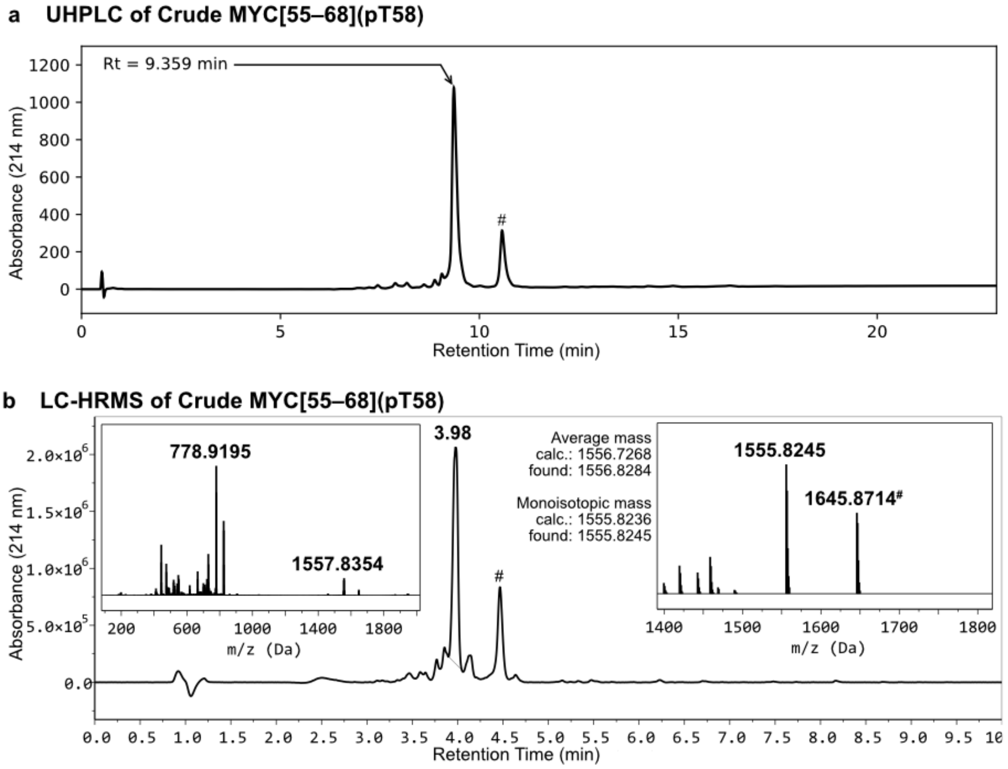

Figure S21. (a) UHPLC profile of crude MYC[55–68](pT58); Rt = 9.359 min, 66% purity. Measured according to **General Procedure 3.3**. (b) LC-HRMS profile of crude MYC[55–68](pT58); Rt = 3.98 min, measured according to **General Procedure 3.4**. Insert (left) = ESI-TOF spectrum. Insert (right) = deconvoluted mass spectra. Monoisotopic mass (ESI+) calc. for $C_{65}H_{114}N_{21}O_{21}P$ 1555.8236, found 1555.8232. #Benzyl adduct.

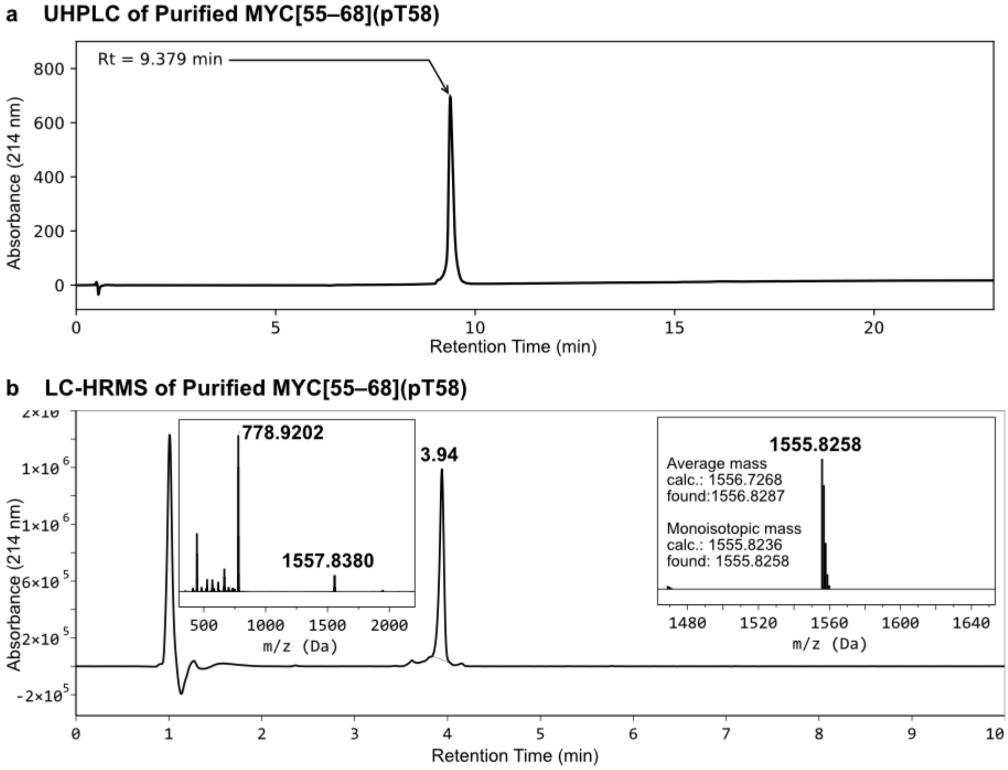

Figure S22. (a) UHPLC profile of purified MYC[55–68](pT58); Rt = 9.379 min, 95% purity. Measured according to **General Procedure 3.3**. (b) LC-HRMS profile of purified MYC[55–68](pT58); Rt = 3.94 min, measured according to **General Procedure 3.4**. Insert (left) = ESI-TOF spectrum. Insert (right) = deconvoluted mass spectra. Monoisotopic mass (ESI+) calc. for $C_{65}H_{114}N_{21}O_{21}P$ 1555.8236, found 1555.8258.

4.4.2 MYC[55–68](pS62)

```
55          61
LLPTPP LpSPSRRSG-NH2  (14 AA)
```

The peptide MYC[55–68](pS62) was prepared via AFPS using Novabiochem® NovaPEG Rink Amide resin (0.41 mmol/g, 0.12 g, 49 µmol) using methods outlined in **General Procedure 3.1**. Total synthesis time to afford resin-bound MYC[55–68](pS62) (0.18 g) was approximately 0.5 h. Cleavage of the peptidyl-resin (73 mg) following **General Procedure 3.2** afforded the crude peptide (approx. 85% crude purity by UHPLC, monoisotopic mass calc. 1555.8236, found 1555.8253), **Figure S23**. The crude peptide was then purified by semi-prep RP-HPLC following **General Procedure 3.5** with a gradient of 5–60%B over 55 min (*ca.* 1%B/min). Fractions were analyzed by LC-HRMS and UHPLC, combined, and lyophilized to afford MYC[55–68](pS62) (11 mg, 95% purity by UHPLC, 39% overall yield, monoisotopic mass calc. 1555.8236, found 1555.8231), **Figure S24**.

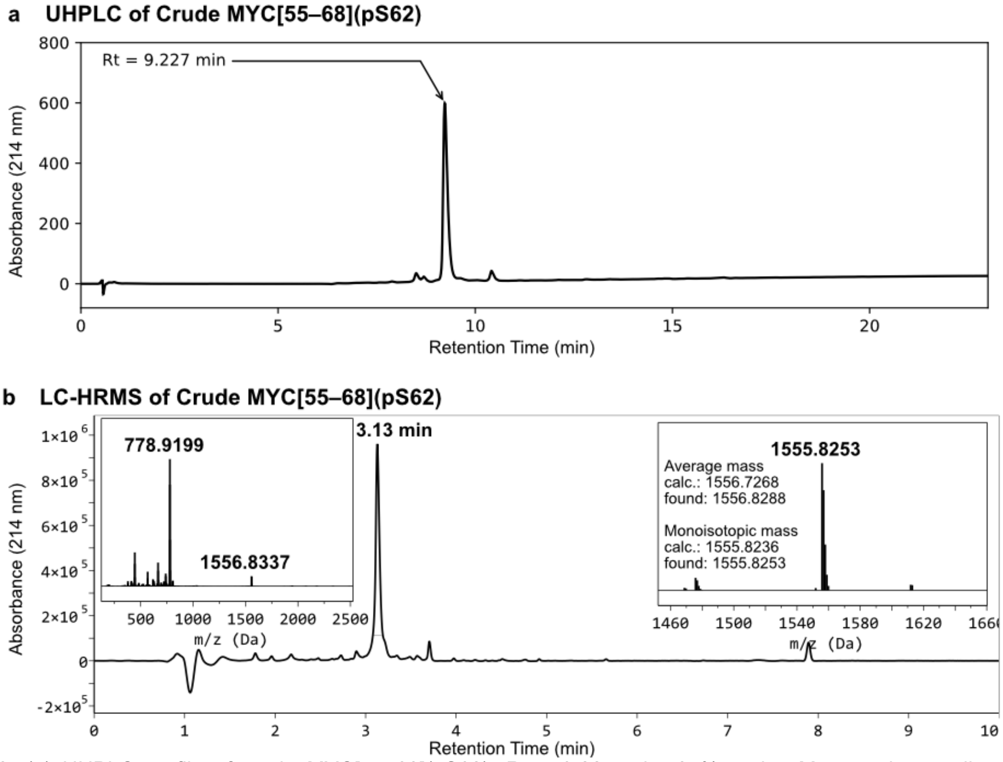

Figure S23. (a) UHPLC profile of crude MYC[55–68](pS62); Rt = 9.227 min, 85% purity. Measured according to **General Procedure 3.3**. (b) LC-HRMS profile of crude MYC[55–68](pS62); Rt = 3.13 min,# measured according to **General Procedure 3.4**. Insert (left) = ESI-TOF spectrum. Insert (right) = deconvoluted mass spectra. Monoisotopic mass (ESI+) calc. for $C_{65}H_{114}N_{21}O_{21}P$ 1555.8236, found 1555.8253. #Retention time shift compared to pure profile due to LC-HRMS pump error.

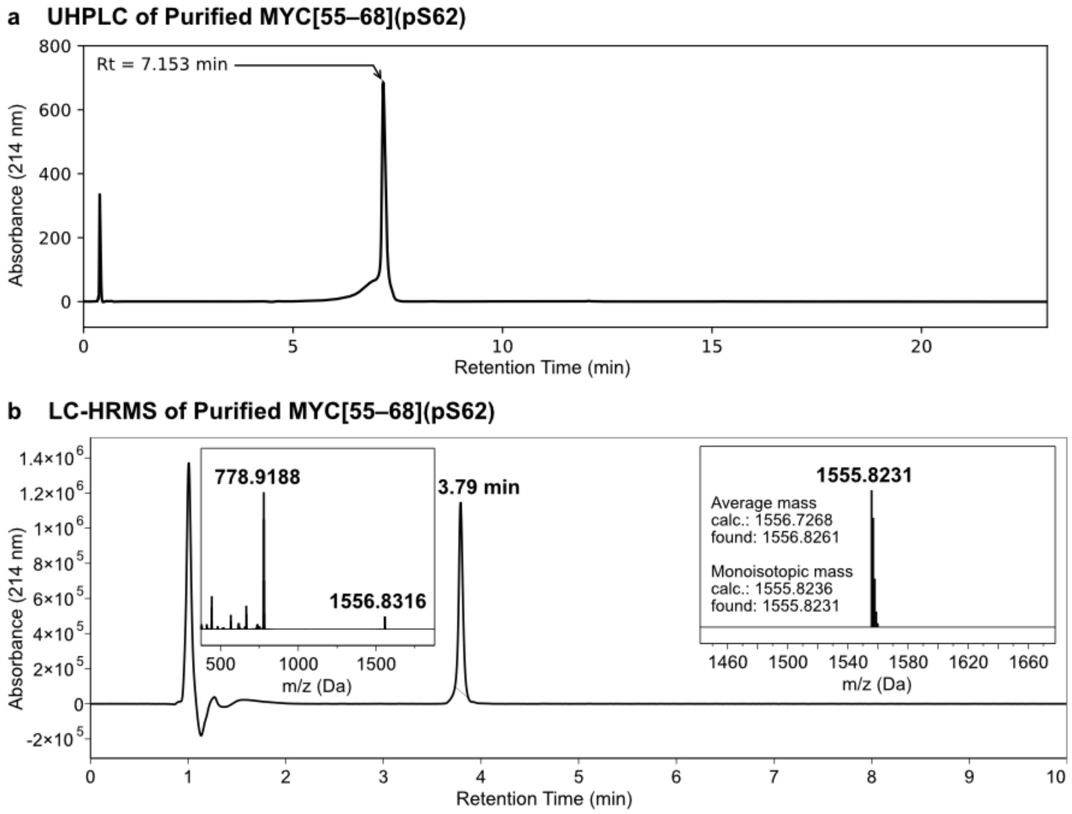

Figure S24. (a) UHPLC profile of purified MYC[55–68](pS62); Rt = 7.153 min, 95% purity. Measured according to **General Procedure 3.3**. (b) LC-HRMS profile of purified MYC[55–68](pS62); Rt = 3.79 min, measured according to **General Procedure 3.4**. Insert (left) = ESI-TOF spectrum. Insert (right) = deconvoluted mass spectra. Monoisotopic mass (ESI+) calc. for $C_{65}H_{114}N_{21}O_{21}P$ 1555.8236, found 1555.8231.

4.4.3 MYC[55–68](pT58, pS62)

```
55        61
LLPpTPP   LpSPSRRSG-NH2   (14 AA)
```

The peptide MYC[55–68](pT58, pS62) was prepared via AFPS using Novabiochem® NovaPEG Rink Amide resin (0.41 mmol/g, 0.11 g, 45 μmol) using methods outlined in **General Procedure 3.1**. Total synthesis time to afford resin-bound MYC[55–68](pT58, pS62) (0.18 g) was approximately 0.5 h. Cleavage of the peptidyl-resin (91 mg) following **General Procedure 3.2** afforded the crude peptide (approx. 76% crude purity by UHPLC, monoisotopic mass calc. 1635.7899, found 1635.7899), **Figure S25**, which was then purified by semi-prep RP-HPLC following **General Procedure 3.5** with a gradient of 5–60%B over 55 min (*ca.* 1%B/min). Fractions were analyzed by LC-HRMS and UHPLC, combined, and lyophilized to afford MYC[55–68](pT58, pS62) (7.0 mg, >95% purity by UHPLC, 20% overall yield, monoisotopic mass calc. 1635.7899, found 1635.7935), **Figure S26**.

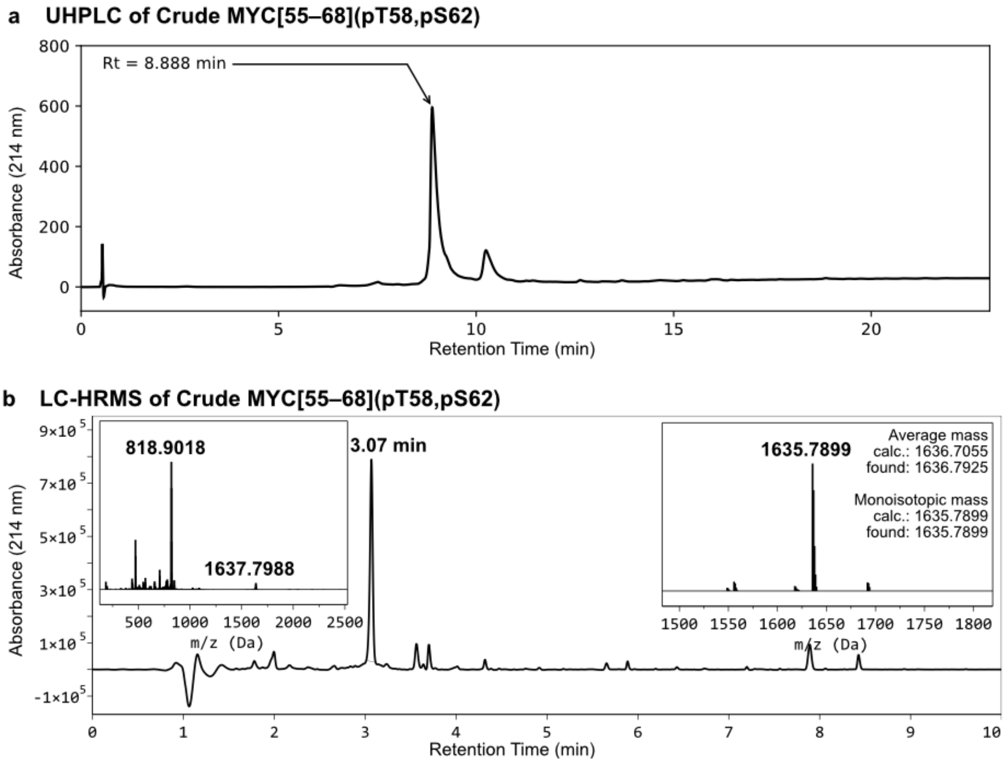

Figure S25. (a) UHPLC profile of crude MYC[55–68](pT58,pS62); Rt = 8.888 min, 76% purity. Measured according to **General Procedure 3.3**. (b) LC-HRMS profile of crude MYC[55–68](pT58,pS62); Rt = 3.07 min, measured according to **General Procedure 3.4**. Insert (left) = ESI-TOF spectrum. Insert (right) = deconvoluted mass spectra. Monoisotopic mass (ESI+) calc. for $C_{65}H_{115}N_{21}O_{24}P_2$ 1635.7899, found 1635.7899. #Retention time shift compared to pure profile due to LC-HRMS pump error.

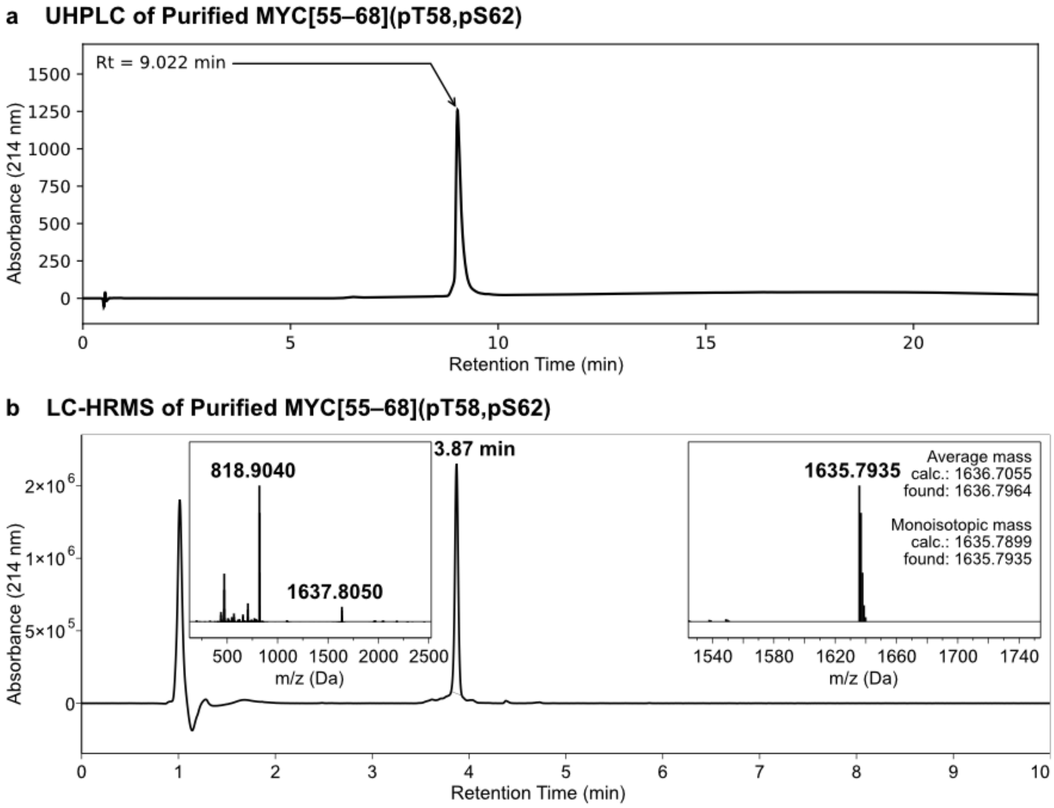

Figure S26. (a) UHPLC profile of purified MYC[55–68](pT58,pS62); Rt = 9.022 min, >95% purity. Measured according to **General Procedure 3.3**. (b) LC-HRMS profile of purified MYC[55–68](pT58,pS62); Rt = 3.87 min, measured according to **General Procedure 3.4**. Insert (left) = ESI-TOF spectrum. Insert (right) = deconvoluted mass spectra. Monoisotopic mass (ESI+) calc. for $C_{65}H_{115}N_{21}O_{24}P_2$ 1635.7899, found 1635.7935.

4.4.4 MYC[55–68](GlcNAc-T58)

```
55         61
LLPgTPP    LSPSRRSG-NH2   (14 AA)
```

The peptide MYC[59–68] was first prepared via AFPS using Novabiochem® NovaPEG Rink Amide resin (0.20 mmol/g, 0.16 g, 32 µmol) using methods outlined in **General Procedure 3.1** affording resin-bound MYC[59–68] (0.19 g). Half of the peptidyl-resin (95 mg, approx. 16 µmol), was then swelled in DMF for 10 min, then drained. Fmoc-L-Thr(ß-D-GlcNAc(Ac)$_3$)-OH (21.5 mg, 32 µmol) was pre-activated at room temperature for 1 min using HATU (84 µL, 0.38 M, 32 µmol) and DIPEA (11 µL, 64 µmol) in DMF (2 mL). This solution was then added to the peptidyl-resin and allowed to react at room temperature with agitation for 90 min. The resin was then drained and washed with DMF (3 × 3 mL). Removal of the N^{α}-Fmoc group and subsequent elongation of the peptide was carried out via AFPS using methods outlined in **General Procedure 3.1** to afford resin-bound MYC[55–68](GlcNAc(Ac)$_3$-T58). For deacetylation, the peptidyl-resin was treated with $H_2NNH_2 \cdot H_2O$ and MeOH (8:2, *v*/*v*, 2.5 mL) at room temperature with agitation for 30 min, then washed with MeOH:CH_2Cl_2 (1:1, 3 × 3 mL) and CH_2Cl_2 (6 × 3 mL). The deacetylation procedure was repeated, and the peptide dried under vacuum after the final washing step. Cleavage of the deacetylated peptidyl-resin following **General Procedure 3.2** afforded crude MYC[55–68](GlcNAc-T58) (approx. 71% crude purity by UHPLC, monoisotopic mass calc. 1678.9366, found 1678.9388), **Figure S27**, which was then purified by semi-prep RP-HPLC following **General Procedure 3.5** with a gradient of 5–60%B over 55 min (*ca.* 1%B/min). Fractions were analyzed by LC-HRMS and UHPLC, combined, and lyophilized to afford MYC[55–68](GlcNAc-T58) (8.6 mg, 95% purity by UHPLC, 32% overall yield, monoisotopic mass calc. 1678.9366, found 1678.9396), **Figure S28**.

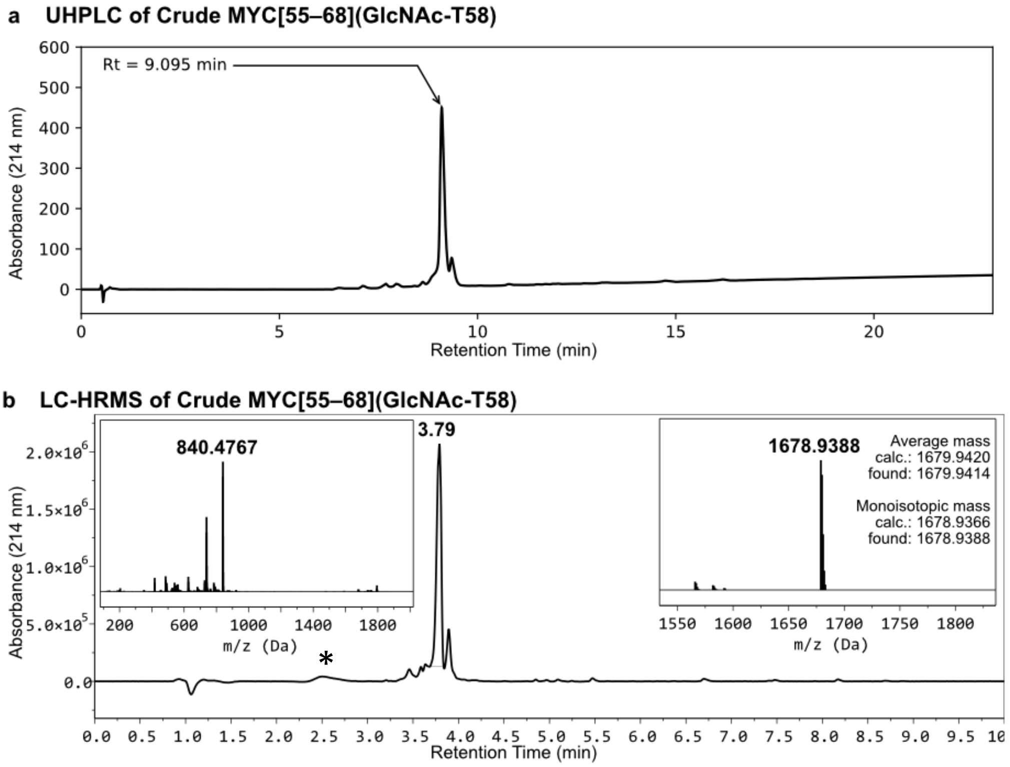

Figure S27. (a) UHPLC profile of crude MYC[55–68](GlcNAc-T58); Rt = 9.095 min, 71% purity. Measured according to **General Procedure 3.3**. (b) LC-HRMS profile of crude MYC[55–68](GlcNAc-T58); Rt = 3.79 min, measured according to **General Procedure 3.4**. Insert (left) = ESI-TOF spectrum. *Solvent wave.[51] Insert (right) = deconvoluted mass spectra. Monoisotopic mass (ESI+) calc. for $C_{73}H_{126}N_{22}O_{23}$ 1678.9366, found 1678.9388.

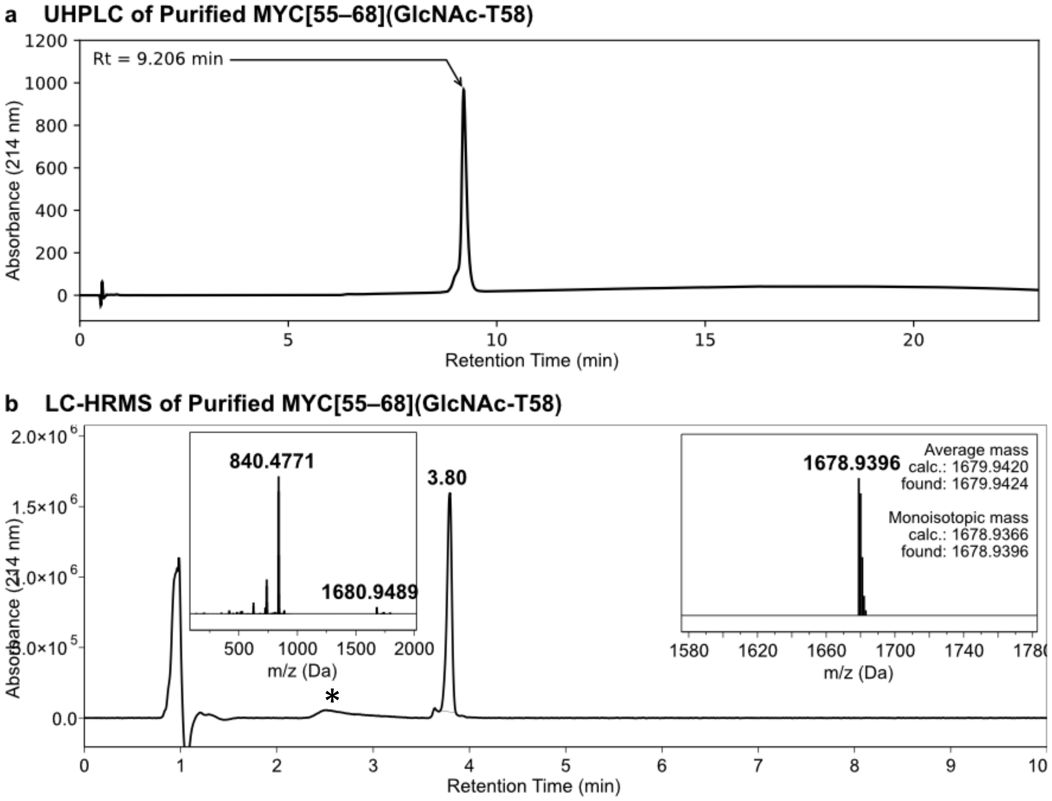

Figure S28. (a) UHPLC profile of purified MYC[55–68](GlcNAc-T58); Rt = 9.206 min, 95% purity. Measured according to **General Procedure 3.3**. (b) LC-HRMS profile of purified MYC[55–68](GlcNAc-T58); Rt = 3.80 min, measured according to **General Procedure 3.4**. *Solvent wave.[51] Insert (left) = ESI-TOF spectrum. Insert (right) = deconvoluted mass spectra. Monoisotopic mass (ESI+) calc. for $C_{73}H_{126}N_{22}O_{23}$ 1678.9366, found 1678.9396.

4.5 Synthesis of PTM-modified MYC[1–84]

4.5.1 MYC[1–84]

```
1          11         21         31         41         51
MPLNVSFTNR NYDLDYDSVQ PYFYCDEEEN FYQQQQQSEL QPPAPSEDIW KKFELLPTPP

61         71         81
LSPSRRSGLC SPSYVAVTPF SLRG-NH2   (84 AA)
```

The peptide MYC[1–84] was prepared via AFPS using Novabiochem® NovaPEG Rink Amide resin (0.20 mmol/g, 0.16 g, 31 μmol) using methods outlined in **General Procedure 3.1**. The peptidyl-resin was collected, washed with CH_2Cl_2 (3 × 3 mL) and dried under vacuum. Total synthesis time to afford resin-bound MYC[1–84] was approximately 3.5 h. Cleavage of the peptidyl-resin following **General Procedure 3.2** afforded the crude peptide (approx. 55% crude purity by UHPLC, monoisotopic mass calc. 9747.6443, found 9749.6805), **Figure S29**, which was then purified by semi-prep RP-HPLC following **General Procedure 3.5** with a two-step gradient of 20–30%B over 20 min (*ca.* 0.5%B/min), followed by 30–60%B over 120 min (*ca.* 0.25%B/min). Fractions were analyzed by LC-HRMS and UHPLC, combined, and lyophilized to afford MYC[1–84] (6.9 mg, >95% purity by UHPLC, 2.4% overall yield, monoisotopic mass calc. 9747.6443, found 9748.6463), **Figure S30**.

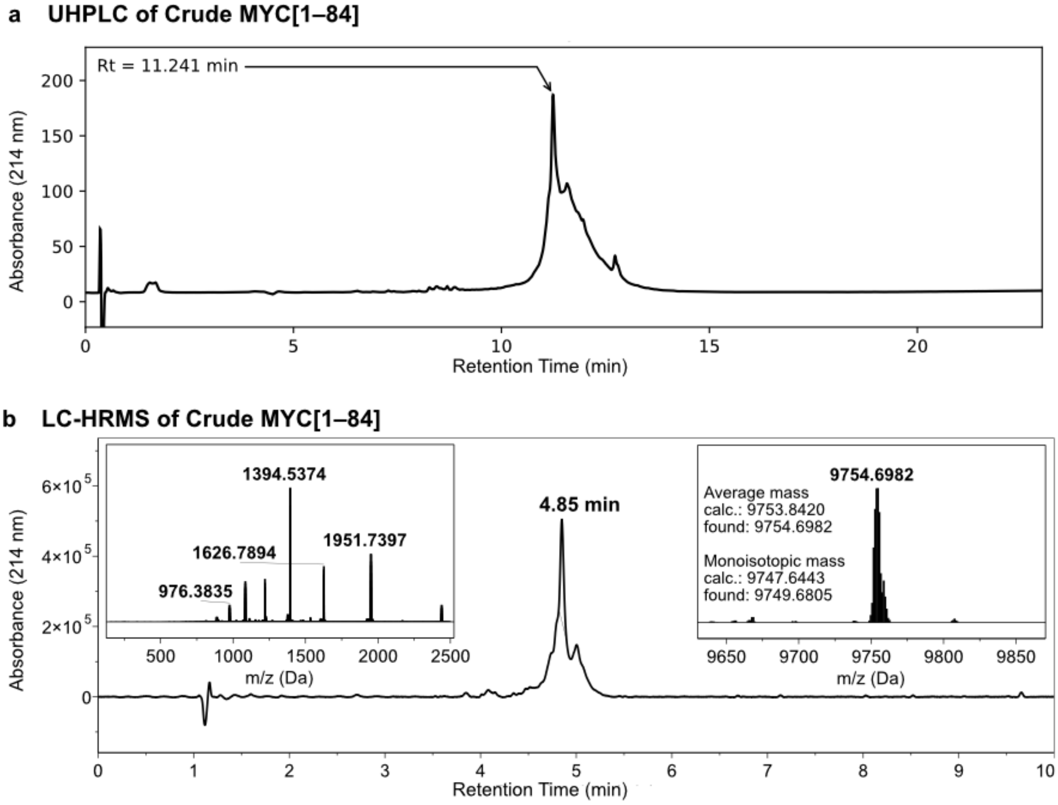

Figure S29. (a) UHPLC profile of crude MYC[1–84]; Rt = 11.241 min, 55% purity. Measured according to **General Procedure 3.3**. (b) LC-HRMS profile of crude MYC[1–84]; Rt = 4.85 min, measured according to **General Procedure 3.4**. Insert (left) = ESI-TOF spectrum. Insert (right) = deconvoluted mass spectra. Monoisotopic mass (ESI+) calc. for $C_{439}H_{649}N_{111}O_{136}S_3$ 9747.6443, found 9749.6805.

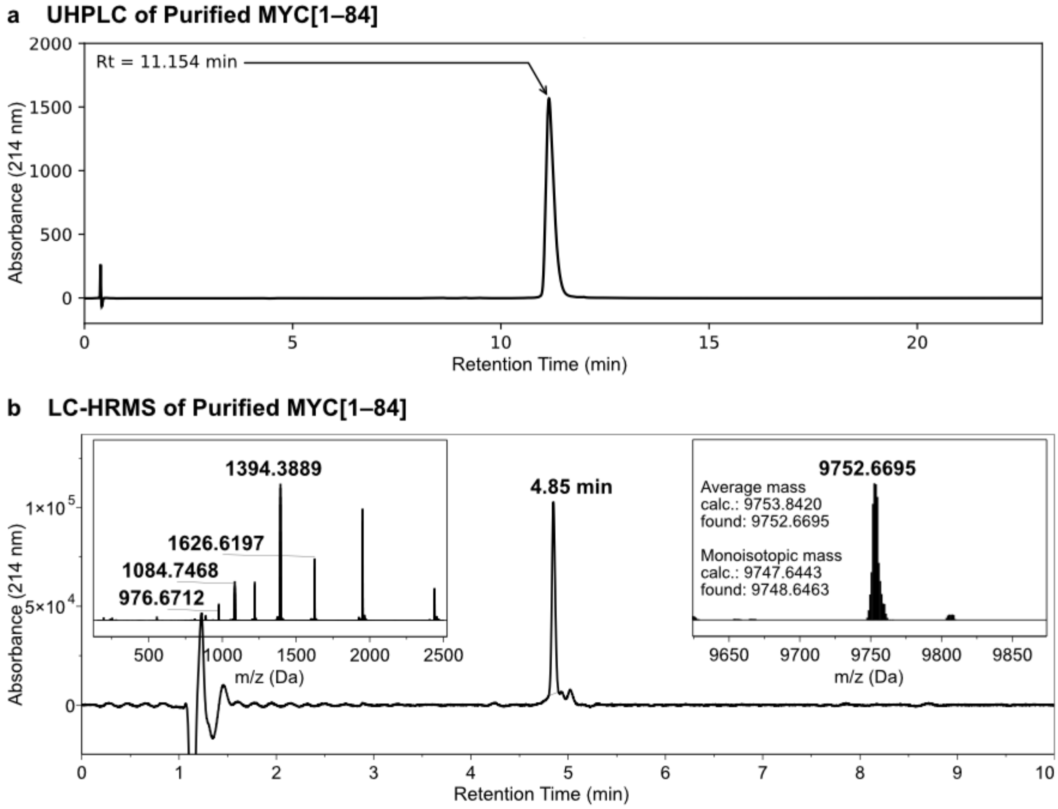

Figure S30. (a) UHPLC profile of purified MYC[1–84]; Rt = 11.154 min, >95% purity. Measured according to **General Procedure 3.3**. (b) LC-HRMS profile of purified MYC[1–84]; Rt = 4.85 min, measured according to **General Procedure 3.4**. *Solvent wave. Insert (left) = ESI-TOF spectrum. Insert (right) = deconvoluted mass spectra. Monoisotopic mass (ESI+) calc. for $C_{439}H_{649}N_{111}O_{136}S_3$ 9747.6443, found 9748.6463.

4.5.2 MYC[1–84](pS62)

```
1          11         21         31         41         51
MPLNVSFTNR NYDLDYDSVQ PYFYCDEEEN FYQQQQQSEL QPPAPSEDIW KKFELLPTPP

61         71         81
LpSPSRRSGLC SPSYVAVTPF SLRG-NH2   (84 AA)
```

The peptide MYC[1–84](pS62) was prepared via AFPS using Novabiochem® NovaPEG Rink Amide resin (0.20 mmol/g, 0.15 g, 31 μmol) using methods outlined in **General Procedure 3.1**. Total synthesis time to afford resin-bound MYC[1–84](pS62) was approximately 3.75 h. Cleavage of the peptidyl-resin following **General Procedure 3.2** afforded the crude peptide (approx. 49% crude purity by UHPLC, monoisotopic mass calc. 9827.6106, found 9827.6858), **Figure S31**, which was then purified by semi-prep RP-HPLC following **General Procedure 3.5** with a two-step gradient of 20–30%B over 20 min (*ca.* 0.5%B/min), followed by 30–60%B over 120 min (*ca.* 0.25%B/min). Fractions were analyzed by LC-HRMS and UHPLC, combined, and lyophilized to afford MYC[1–84](pS62) (2.8 mg, >95% purity by UHPLC, 0.9% overall yield, monoisotopic mass calc. 9827.6106, found 9828.8641), **Figure S32**.

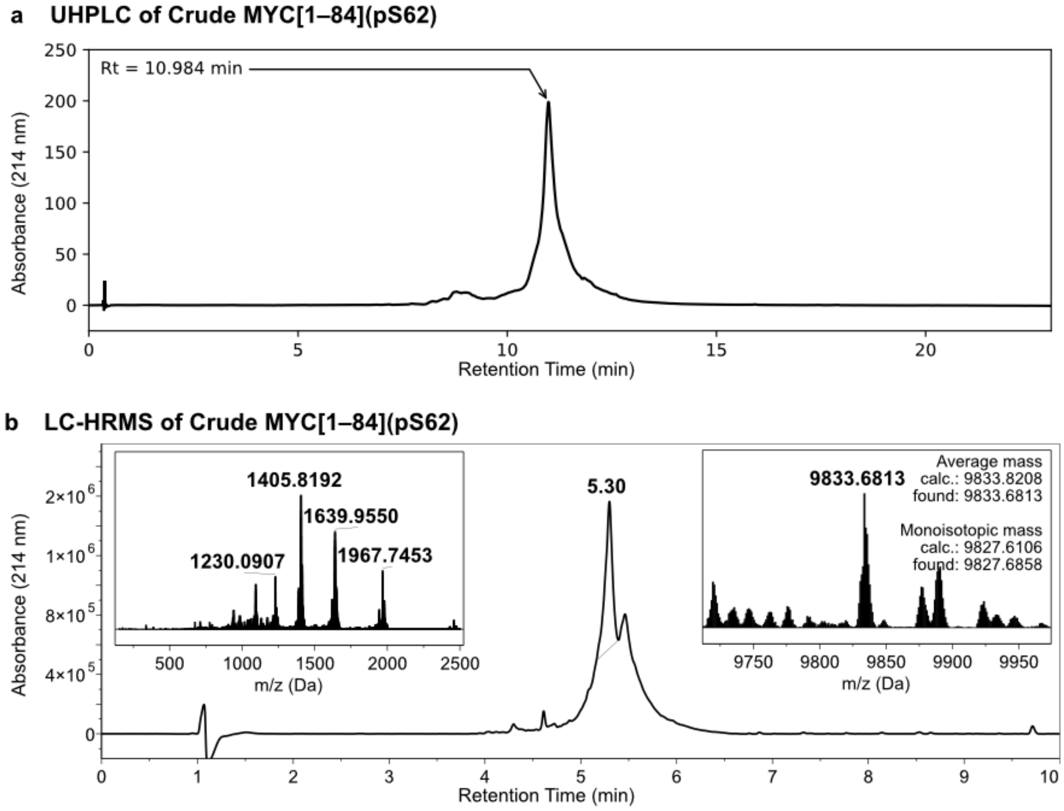

Figure S31. (a) UHPLC profile of crude MYC[1–84](pS62); Rt = 10.984 min, 49% purity. Measured according to **General Procedure 3.3**. (b) LC-HRMS profile of crude MYC[1–84](pS62); Rt = 5.30 min, measured according to **General Procedure 3.4**. Insert (left) = ESI-TOF spectrum. Insert (right) = deconvoluted mass spectra. Monoisotopic mass (ESI+) calc. for $C_{439}H_{650}N_{111}O_{139}PS_3$ 9827.6106, found 9827.6858.

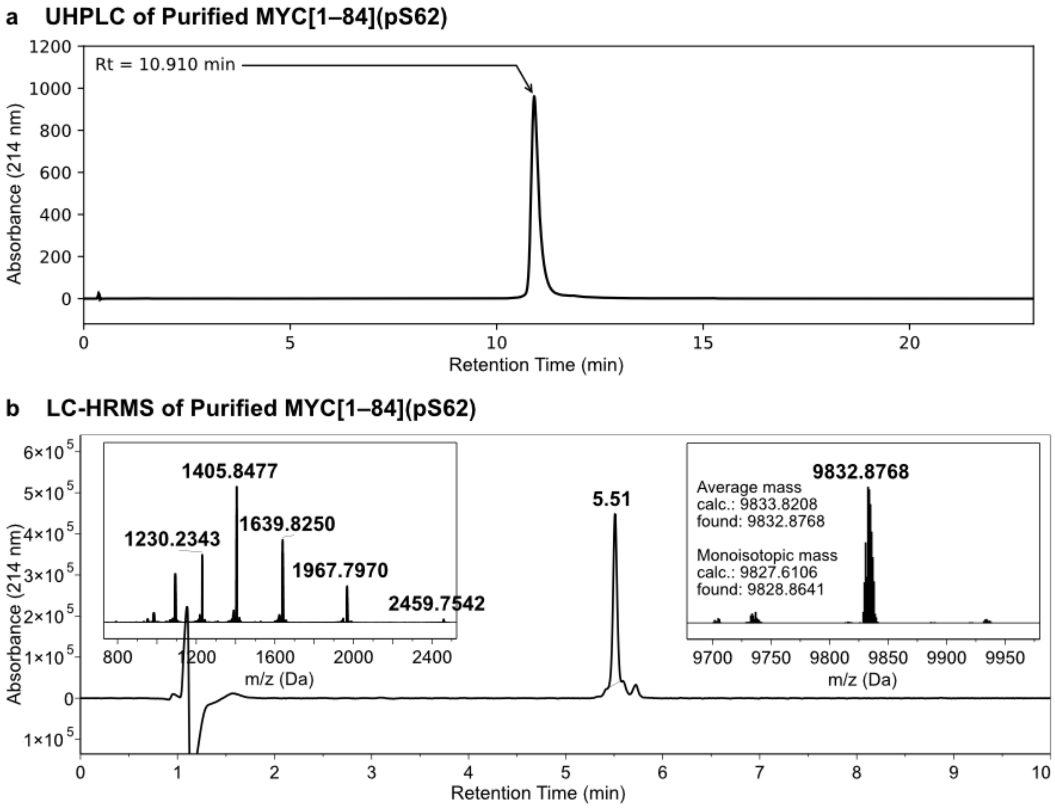

Figure S32. (a) UHPLC profile of purified MYC[1–84](pS62); Rt = 10.910 min, >95% purity. Measured according to **General Procedure 3.3**. (b) LC-HRMS profile of purified MYC[1–84](pS62); Rt = 5.51 min, measured according to **General Procedure 3.4**. Insert (left) = ESI-TOF spectrum. Insert (right) = deconvoluted mass spectra. Monoisotopic mass (ESI+) calc. for $C_{439}H_{650}N_{111}O_{139}PS_3$ 9827.6106, found 9828.8641.

4.5.3 MYC[1–84](pT58)

```
1          11         21         31         41         51
MPLNVSFTNR NYDLDYDSVQ PYFYCDEEEN FYQQQQQSEL QPPAPSEDIW KKFELLPpTPP

61         71         81
LSPSRRSGLC SPSYVAVTPF SLRG-NH2   (84 AA)
```

The peptide MYC[1–84](pT58) was prepared via AFPS using Novabiochem® NovaPEG Rink Amide resin (0.20 mmol/g, 0.15 g, 31 μmol) using methods outlined in **General Procedure 3.1**. Total synthesis time to afford resin-bound MYC[1–84](pT58) was approximately 3.75 h. Cleavage of the peptidyl-resin following **General Procedure 3.2** afforded the crude peptide (approx. 41% crude purity by UHPLC, monoisotopic mass calc. 9827.6106, found 9827.6301), **Figure S33**, which was then purified by semi-prep RP-HPLC following **General Procedure 3.5** with a two-step gradient of 20–30%B over 20 min (*ca.* 0.5%B/min), followed by 30–60%B over 120 min (*ca.* 0.25%B/min). Fractions were analyzed by LC-HRMS and UHPLC, combined, and lyophilized to afford MYC[1–84](pT58) (1.4 mg, >95% purity by UHPLC, 0.5% overall yield, monoisotopic mass calc. 9827.6106, found 9827.6389), **Figure S34**.

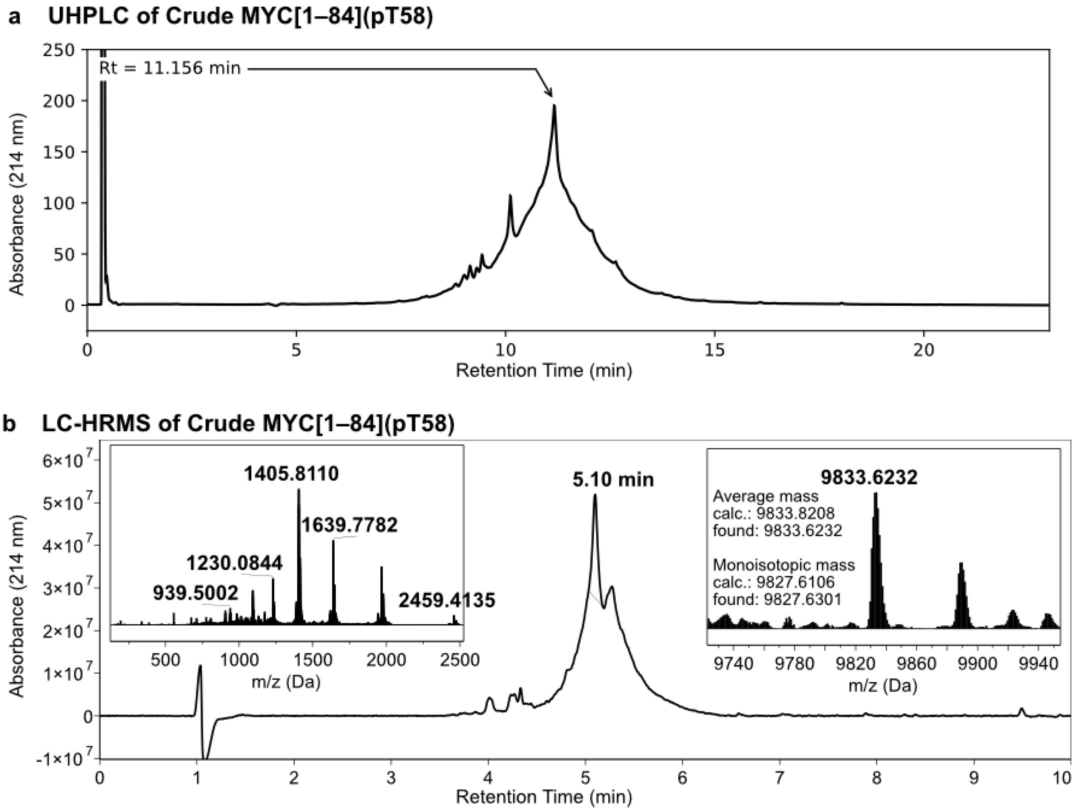

Figure S33. (a) UHPLC profile of crude MYC[1–84](pT58); Rt = 11.156 min, 41% purity. Measured according to **General Procedure 3.3**. (b) LC-HRMS profile of crude MYC[1–84](pT58); Rt = 5.10 min, measured according to **General Procedure 3.4**. Insert (left) = ESI-TOF spectrum. Insert (right) = deconvoluted mass spectra. Monoisotopic mass (ESI+) calc. for $C_{439}H_{650}N_{111}O_{139}PS_3$ 9827.6106, found 9827.6301.

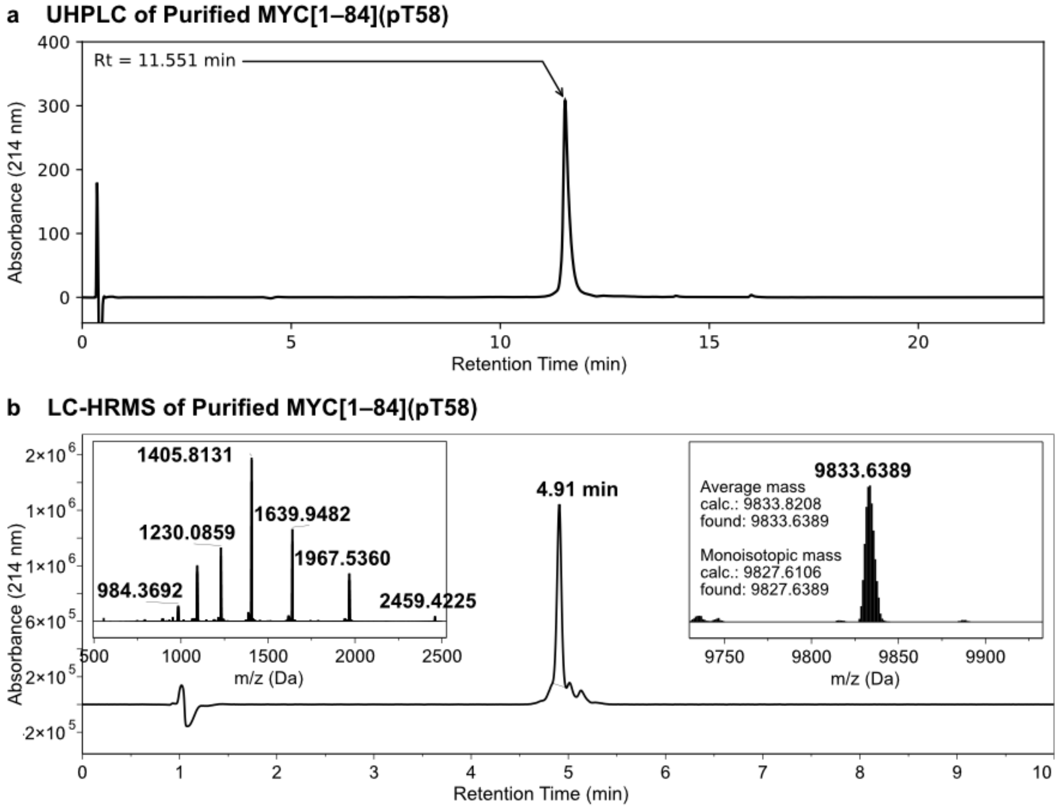

Figure S34. (a) UHPLC profile of purified MYC[1–84](pT58); Rt = 11.551 min, >95% purity. Measured according to **General Procedure 3.3**. (b) LC-HRMS profile of purified MYC[1–84](pT58); Rt = 4.91 min, measured according to **General Procedure 3.4**. Insert (left) = ESI-TOF spectrum. Insert (right) = deconvoluted mass spectra. Monoisotopic mass (ESI+) calc. for $C_{439}H_{650}N_{111}O_{139}PS_3$ 9827.6106, found 9827.6389.

4.5.4 MYC[1–84](GlcNAc-T58)

```
1          11         21         31         41         51
MPLNVSFTNR NYDLDYDSVQ PYFYCDEEEN FYQQQQQSEL QPPAPSEDIW KKFELLPgTPP

61         71         81
LSPSRRSGLC SPSYVAVTPF SLRG-NH2   (84 AA)
```

The peptide MYC[59–84] was first prepared via AFPS using Novabiochem® NovaPEG Rink Amide resin (0.20 mmol/g, 0.15 g, 31 μmol) using methods outlined in **General Procedure 3.1**. The peptidyl-resin was swelled in DMF for 10 min, then drained. Fmoc-L-Thr(ß-D-GlcNAc(Ac)$_3$)-OH (42 mg, 62 μmol) was pre-activated at room temperature for 1 min using HATU (0.15 mL, 0.38 M, 56 μmol) and *i*Pr_2NEt (53 μL, 0.31 mmol) in DMF (2 mL). This solution was then added to the peptidyl-resin and allowed to react at room temperature with agitation for 30 min. The resin was then drained and washed with DMF (3 × 3 mL). Removal of the N^{α}-Fmoc group and subsequent elongation of the peptide to afford MYC[1–84](GlcNAc(Ac)$_3$-T58) was carried out via AFPS using methods outlined in **General Procedure 3.1**. For deacetylation, the elongated peptidyl-resin was treated with $H_2NNH_2 \cdot H_2O$ and MeOH (4:1, *v*/*v*, 6.0 mL) at room temperature with agitation for 30 min, then drained and washed with MeOH:CH_2Cl_2 (1:1, 3 × 3 mL) and CH_2Cl_2 (6 × 3 mL). The deacetylation procedure was repeated, and the peptide dried under vacuum after the final washing step. Cleavage of the deacetylated peptidyl-resin following **General Procedure 3.2** afforded crude MYC[1–84](GlcNAc-T58) (approx. 45% crude purity by UHPLC, monoisotopic mass calc. 9950.7236, found 9950.7150), **Figure S35**, which was then purified by semi-prep RP-HPLC following **General Procedure 3.5** with a two-step gradient of 20–30%B over 20 min (*ca.* 0.5%B/min), followed by 30–60%B over 120 min (*ca.* 0.25%B/min). Fractions were analyzed by LC-HRMS and UHPLC, combined, and lyophilized to afford MYC[1–84](GlcNAc-T58) (2.6 mg, 95% purity by UHPLC, 0.9% overall yield, monoisotopic mass calc. 9950.7236, found 9950.7327), **Figure S36**.

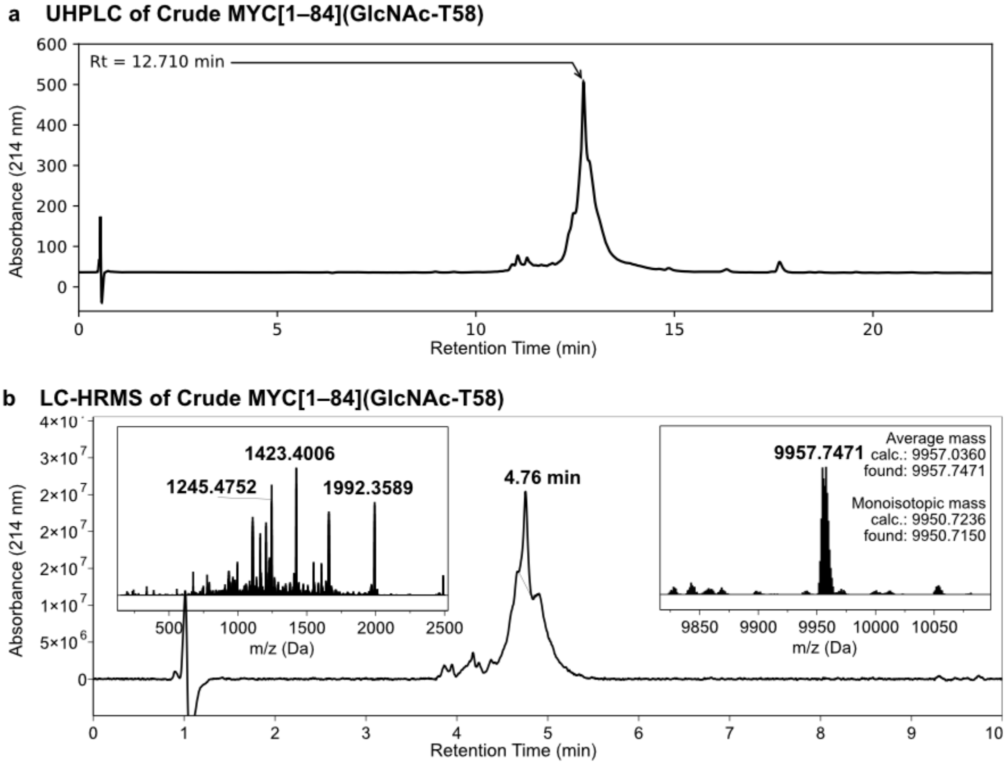

Figure S35. (a) UHPLC profile of crude MYC[1–84](GlcNAc-T58); Rt = 12.710 min, 45% purity. Measured according to **General Procedure 3.3**. (b) LC-HRMS profile of crude MYC[1–84](GlcNAc-T58); Rt = 4.76 min, measured according to **General Procedure**

3.4. Insert (left) = ESI-TOF spectrum. Insert (right) = deconvoluted mass spectra. Monoisotopic mass (ESI+) calc. for $C_{447}H_{662}N_{112}O_{141}S_3$ 9950.7236, found 9950.7150.

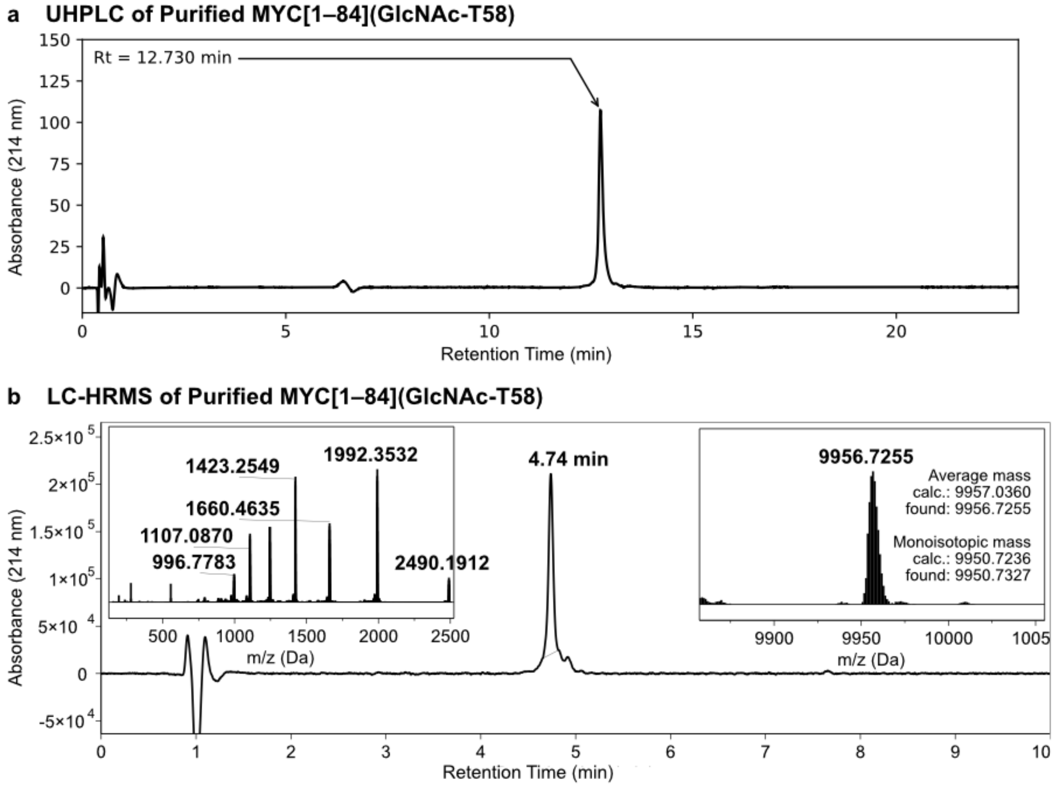

Figure S36. (a) UHPLC profile of purified MYC[1–84](GlcNAc-T58); Rt = 12.730 min, >95% purity. Measured according to **General Procedure 3.3**. (b) LC-HRMS profile of purified MYC[1–84](GlcNAc-T58); Rt = 4.74 min, measured according to **General Procedure 3.4**. Insert (left) = ESI-TOF spectrum. Insert (right) = deconvoluted mass spectra. Monoisotopic mass (ESI+) calc. for $C_{447}H_{662}N_{112}O_{141}S_3$ 9950.7236, found 9950.7327.

4.5.5 MYC[1–84](pT58, pS62)

```
1          11         21         31         41         51
MPLNVSFTNR NYDLDYDSVQ PYFYCDEEEN FYQQQQQSEL QPPAPSEDIW KKFELLPpTPP

61         71         81
LpSPSRRSGLC SPSYVAVTPF SLRG-NH2   (84 AA)
```

The peptide MYC[1–84](pT58,pS62) was prepared via AFPS using Novabiochem® NovaPEG Rink Amide resin (0.20 mmol/g, 0.15 mg, 31 μmol) using methods outlined in **General Procedure 3.1**. Total synthesis time to afford resin-bound MYC[1–84](pT58,pS62) was approximately 4 h. Cleavage of the peptidyl-resin following **General Procedure 3.2** afforded the crude peptide (0.12 g, approx. 20% crude purity by UHPLC, monoisotopic mass calc. 9907.5769, found 9907.3943), **Figure S37**. The crude peptide (38 mg) was then purified by semi-prep RP-HPLC at room temperature following **General Procedure 3.5** with a two-step gradient of 20–30%B over 20 min (*ca.* 0.5%B/min), followed by 30–60%B over 120 min (*ca.* 0.25%B/min). Fractions were analyzed by LC-HRMS and UHPLC, combined, and lyophilized to afford MYC[1–84](pT58,pS62) (0.9 mg, 92% purity by UHPLC, 0.6% overall yield, monoisotopic mass calc. 9907.5769, found 9907.9806), **Figure S38**.

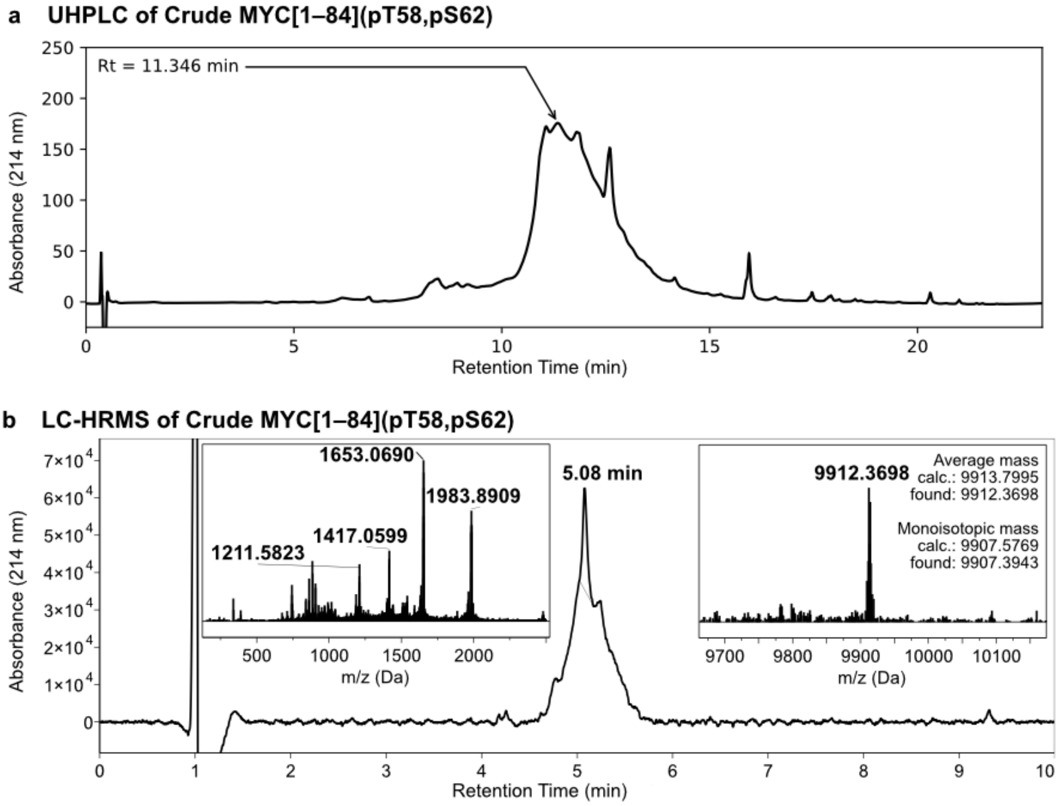

Figure S37. (a) UHPLC profile of crude MYC[1–84](pT58,pS62); Rt = 11.346 min, 20% purity. Measured according to **General Procedure 3.3**. (b) LC-HRMS profile of crude MYC[1–84](pT58,pS62); Rt = 5.08 min, measured according to **General Procedure 3.4**. Insert (left) = ESI-TOF spectrum. Insert (right) = deconvoluted mass spectra. Monoisotopic mass (ESI+) calc. for $C_{439}H_{651}N_{111}O_{142}P_2S_3$ 9907.5769, found 9907.3943.

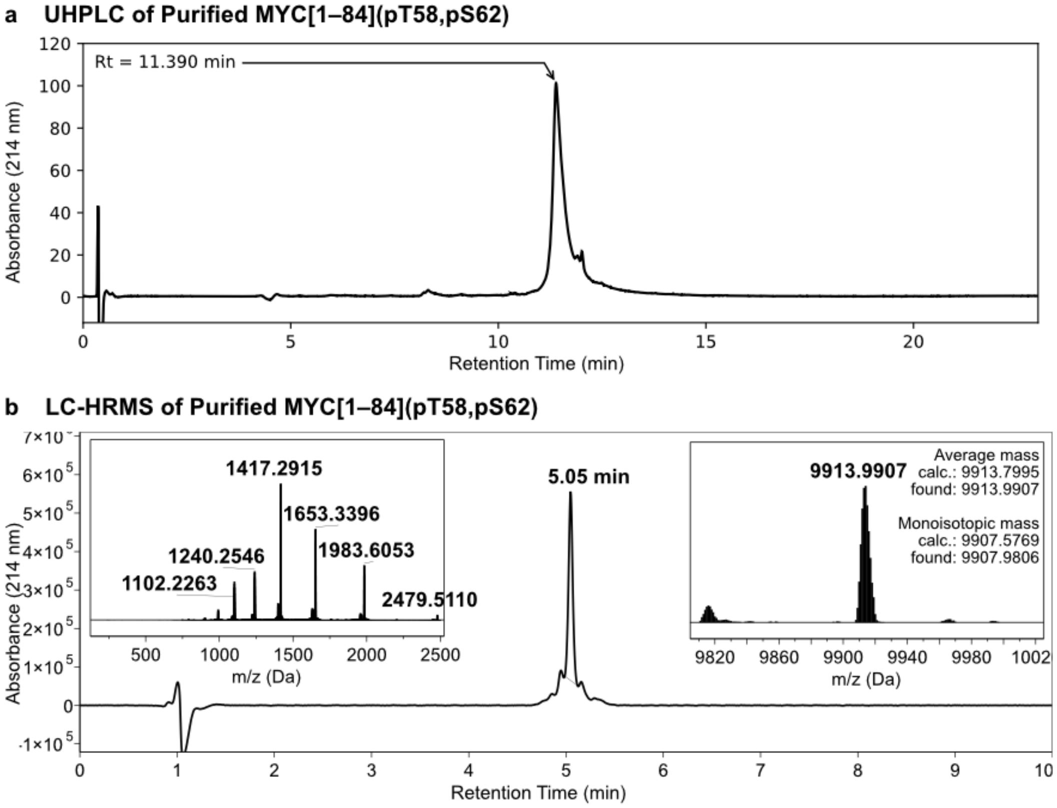

Figure S38. (a) UHPLC profile of purified MYC[1–84](pT58,pS62); Rt = 11.390 min, 92% purity. Measured according to **General Procedure 3.3**. (b) LC-HRMS profile of purified MYC[1–84](pT58,pS62); Rt = 5.05 min, measured according to **General Procedure 3.4**. Insert (left) = ESI-TOF spectrum. Insert (right) = deconvoluted mass spectra. Monoisotopic mass (ESI+) calc. for $C_{439}H_{651}N_{111}O_{142}P_2S_3$ 9907.5769, found 9907.9806.

5 Bin1(SH3) protein expression and purification

His_6-GB1-TEVc-Bin1(SH3)

```
1           11          21          31          41          51
SGSHHHHHHS  SGIEGRGRQY  KLILNGKTLK  GETTTEAVDA  ATAEKVFKQY  ANDNGVDGEW

61          71          81          91          101         111
TYDDATKTFT  VTESSGENLY  FQGRLDLPPG  FMFKVQAQHD  YTATDTDELQ  LKAGDVVLVI

121         131         141         151         161
PFQNPEEQDE  GWLMGVKESD  WNQHKKLEKC  RGVFPENFTE  RVP         (163 AA)
```

Bin1(SH3)

```
1           11          21          31          41          51
GRLDLPPGFM  FKVQAQHDYT  ATDTDELQLK  AGDVVLVIPF  QNPEEQDEGW  LMGVKESDWN

61          71          81
QHKKLEKCRG  VFPENFTERV  P           (81 AA)
```

His_6-GB1-TEVc-Bin1(SH3) (Bin1(SH3) = Bin1 residues 359–439) was ordered as a gBlock (Integrated DNA Technologies©). The construct was digested with SapI and BamHI and cloned into a pEM3BT2 vector. Transformation of *Escherichia coli* BL21-DE3 cells with the vector was achieved by heat shock. The transformed cells were grown in M9 medium (350 mL, KH_2PO_4 [3.0 g/L], $Na_2HPO_4.2H_2O$ [7.5 g/L], NaCl [0.50 g/L], $MgSO_4$ [1.0 mM], trace metals, ampicillin [100 µg/mL], glucose [5.0 g/L], and NH_4Cl [1.2 g/L])* at 37 °C until OD_{600} = 0.6 (approximately 5 h). Protein expression was induced with IPTG (0.5 mM) and the cultures were incubated at 25 °C overnight. The cells were then collected by centrifugation into a pellet and flash-frozen in liquid nitrogen.

The following steps were carried out at 4 °C. The cell pellet (approx. 3 g) was thawed and resuspended with Resuspension Buffer (DNAse [0.05 mg/mL], lysozyme [3 mg/mL], and $MgCl_2$ [5 mM], 15 mL), then treated with Lysis Buffer (phosphate buffer pH 8 [80 mM], NaCl [300 mM], imidazole [40 mM], sodium cholate [2.0 g/L], PMSF [1.0 mM], glycerol [10% *v*/*v*], and Triton X-100 [2% *v*/*v*], 15 mL) for 2 h with gentle agitation. The cell lysate was then centrifuged at 18,000 rpm for 30 min at 4 °C, and the supernatant was collected.

The fusion protein (His_6-GB1-TEVc-Bin1(SH3)) was isolated from the supernatant by gravity-flow Ni-NTA affinity chromatography. Using HisTrap Ni Sepharose FF resin (Cytiva, 2.0 mL) equilibrated with Equilibration Buffer (phosphate buffer pH 8 [20 mM], NaCl [300 mM], and glycerol [10% *v*/*v*]), unbound proteins were washed out with 10 column volumes (CV) of Wash Buffer (phosphate buffer [20 mM] pH 8, NaCl [300 mM], imidazole [30 mM], sodium cholate [20 mM], and glycerol [10% *v*/*v*]). The desired fusion protein was then eluted with 3 × CV of Elution Buffer (phosphate buffer pH 7.5 [20 mM], NaCl [300 mM], and imidazole [300 mM]). To the eluted protein, TEV protease (1.0 mL, 1.0 mg/mL stock solution) was added and the sample was dialyzed in Dialysis Buffer (phosphate buffer pH 7.0 [20 mM], NaCl [600 mM], and glycerol [5% *v*/*v*]) at 4 °C overnight. Reverse Ni-NTA using HisTrap Ni Sepharose FF resin (Cytiva, 2.0 mL) equilibrated with Dialysis Buffer was then performed, affording Bin1(SH3) (10 mg/L culture).

*Uniformly labeled ^{15}N samples were produced using M9 medium containing $^{15}NH_4Cl$ (1.0 g/L). Uniformly 15N- and 13C-labeled samples were produced using M9 medium containing $^{15}NH_4Cl$ (1.0 g/L) and glucose-$^{13}C_6$ (3.0 g/L).

^{15}N-labeled Bin1(SH3): Average mass calc. 9455.5976, found 9457.2912.

6 Native Mass Spectrometry (nMS)

Native mass spectrometry data were acquired on a Waters Synapt G2-Si (Waters Corporation, Wilmslow, UK) instrument with an 8 k quadrupole operated in a "Sensitivity" mode. Proteins were subjected to nESI in positive mode with a nanospray emitter pulled in-house with a Flaming/Brown P-97 micropipette puller from thin-walled glass capillaries (i.d. 0.78 mm, o.d. 1.0 mm, 10 cm length, both from Sutter Instrument Co., Novato, CA, USA). A positive potential of 0.9–1.2 kV was applied to the solution via a thin platinum wire. Other non-default instrument settings include sampling cone voltage 30–60 V, source offset 40–80 V, trap collision energy 5 V, trap gas flow 3.0 mL/min, *m/z* range 750–3000 and source temperature 40 °C. Data were acquired and processed using MassLynx (V4.2, Waters Corporation, Wilmslow, UK).

6.1 nMS: Bin1(SH3) and MYC[55–68] analogues

Bin1(SH3) protein was buffer exchanged into ammonium acetate buffer (10 mM, pH 6.8) (analytical grade, Fisher) using a 96-well microdialysis plate (ThermoScientific) and its concentration was determined using a NanoDrop spectrophotometer (ThermoScientific) using the A280 method. MYC[55–68] peptides were dissolved directly into ammonium acetate buffer (50 mM, pH 6.8), and a Bicinchoninic acid assay (BCA) was utilized for concentration determination. Bin1(SH3) (2.5 µM) was incubated with each MYC[55–68] analogue (50 µM) in ammonium acetate (30 mM, pH 6.8) for 10 min on ice before nMS analysis. All samples and buffer solutions were prepared using ultra-pure water (18.2 MΩ.cm, Millipore).

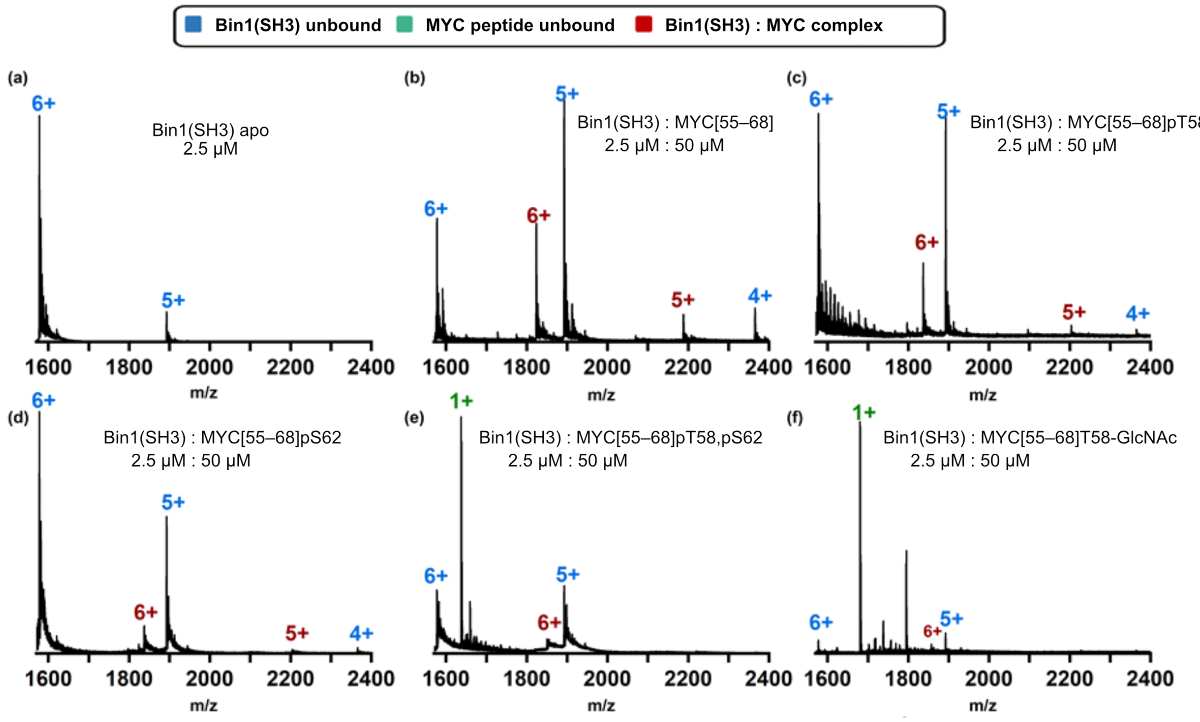

Figure S39. Mass spectra of (a) Bin1(SH3) (2.5 µM). (b-g) Bin1(SH3) (2.5 µM) with MYC[55–68] peptide variants (50 µM each). (b) MYC[55–68], (c) MYC[55–68]pT58, (d) MYC[55–68]pS62, (e) MYC[55–68]pT58,pS62, (f) MYC[55–68]T58-GlcNAc. (b), (c), (d) and (e) are representative spectra of 3 technical repeats. Blue label = unbound Bin1(SH3). Brown label = Bin1(SH3) bound to MYC peptide. Green label = unbound MYC peptide. All samples were analyzed from ammonium acetate buffer (30 mM, pH 6.8).

	Peak intensity (%) of Bin1(SH3):MYC[55–68] complex				
**MYC[55–68] PTMs**	**No PTMs**	**pT58**	**pS62**	**pT58,pS62**	**T58-GlcNAc***
**Repeat Measurements**	27.4	17.0	10.4	9.5	24.8
	25.8	15.4	10.3	12.8	-
	20.7	15.4	7.0	14.1	-
**Average**	24.6	15.9	9.2	12.1	*n/a*
**Standard deviation**	3.5	0.94	1.9	2.4	*n/a*

Table S2. Intensity of MS peaks corresponding to Bin1(SH3):MYC[55–68] complex, as a percentage of total Bin1(SH3) peak intensity (total = [peak intensity of unbound Bin1(SH3)] + [peak intensity of Bin1(SH3):MYC[55–68] complex])

7 Protein NMR

7.1 Peptide and protein concentration determination for NMR experiments

NMR Buffer: Sodium phosphate (20 mM) pH 6.5, NaCl (100 mM), glycerol (5% *v*/*v*), DTT (2 mM). D_2O (7% *v*/*v*) and fresh DTT (1.0 mM) were added to the samples directly before NMR measurements.

Bin1(SH3) and MYC[1–84] protein sample concentrations were measured by NanoDrop at 280 nm. MYC[55–68] peptide concentrations were determined by UHPLC at 214 nm, using MYC[54–68](E54W) as a standard. A dilution series of MYC[54–68](E54W) was measured by NanoDrop at 280 nm, then analyzed by UHPLC (5.0 µL injection). The Area Under the Curve (AUC) of the chromatographic peaks at 214 nm was plotted against the corresponding NanoDrop measurement, with a trendline passing through (0,0). MYC[55–68] analogues were dialyzed in NMR Buffer and analyzed by UHPLC (5 µL injection), and their concentrations calculated using the MYC[54–68](E54W) trendline (y = 18.762x, **Figure S40**).

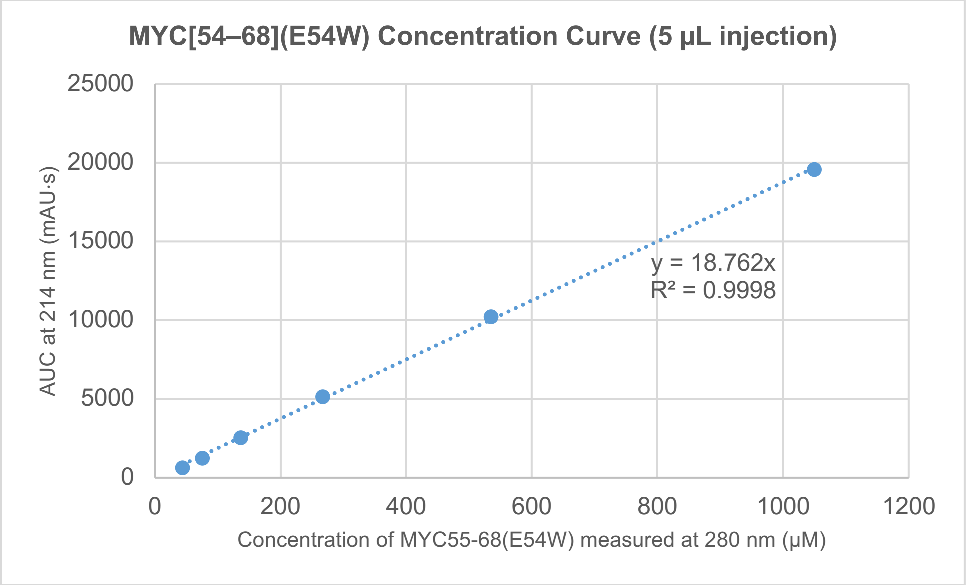

Figure S40. Concentration curve of MYC[54–68](E54W) as measured by NanoDrop (280 nm) and UHPLC (214 nm).

7.2 NMR measurements

All Bin1(SH3) and MYC samples were dialyzed in NMR Buffer overnight prior to NMR experiments. D_2O (7% *v*/*v*) and fresh DTT (1.0 mM) were added to the NMR tubes directly before

measurement. The Bin1(SH3) construct used corresponds to Bin1[359–439] (herein numbered 1–81), which does not include the unstructured N-terminal residues (Bin1 residues 270–358) that are present in the construct used by Pineda-Lucena *et al.* (BMRB 4871, i.e. Bin1[270–439]).[30,54,59] Backbone chemical shifts provided in BMRB entry 4871 were adapted to the truncated construct and the altered pH and temperature using a standard set of 3D triple-resonance experiments ([^{15}N,^{1}H]-HSQC, HNCO, HNCACB, HNCOCACB, ^{15}N-NOESY)[55] using uniformly ^{13}C,^{15}N-labeled protein in the absence of MYC, and compared to the literature.[30,54,59] Gradient-selected HSQC experiments[56] using the Kay-Rance trick for sensitivity enhancement[57] were chosen from the standard Bruker library. In general, 256 increments were recorded in the 2D experiments with spectral widths of 16 ppm (F2) and 35 ppm (F1) at 600 Hz with 32 scans per increment. Samples contained 40 µM ^{15}N-Bin1(SH3) and 80 µM (2.0 equiv.) MYC analogues. Chemical shifts were referenced to the water line at 4.75 ppm at 298 K, from which ^{15}N chemical shifts were referenced indirectly using the Ξ value of 0.1013290.

Chemical shift perturbations ($\Delta\delta_{total}$)[58] were calculated using the equation:

$$\Delta\delta_{total} = \sqrt{(7 \times \Delta\delta_{NH})^2 + (\Delta\delta_N)^2}$$

A weighing factor of 7 was applied to the ^{1}H chemical shift, corresponding to the difference in magnetogyric ratios of ^{15}N and ^{1}H nuclei.

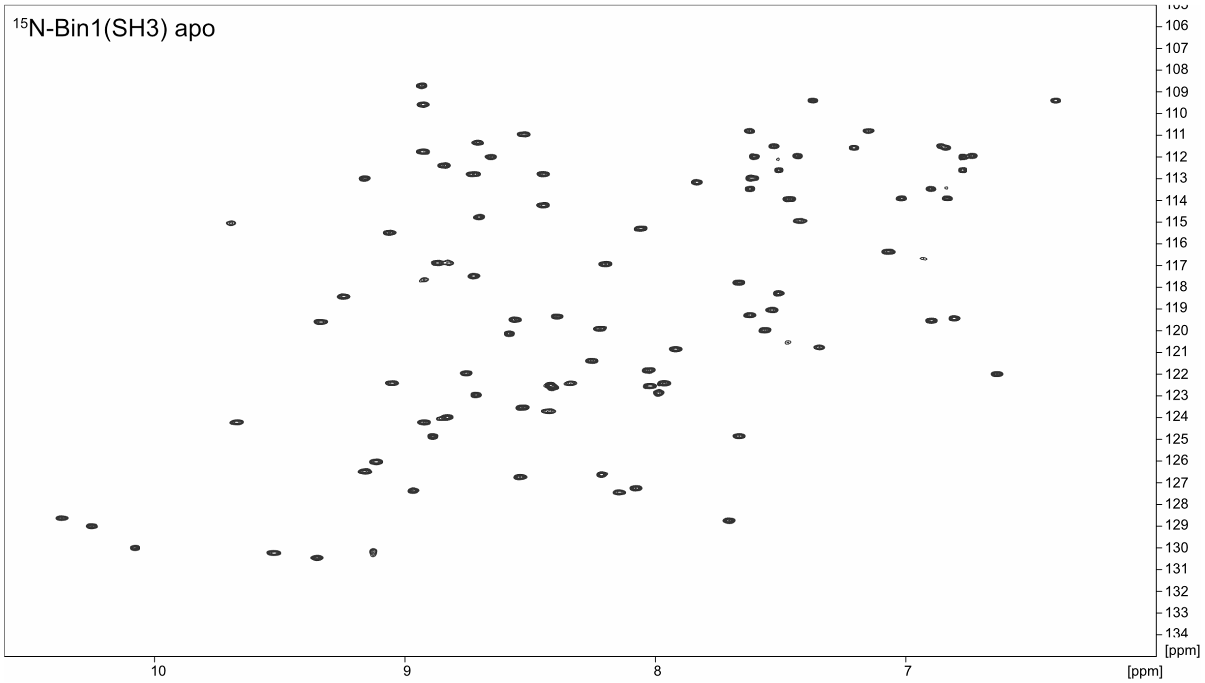

Figure S41. Bin1(SH3) apo measurement, day of MYC[1–84] analogues experiments, after dialysis in NMR buffer. [^{15}N,^{1}H]-HSQC of Bin1(SH3) (40 µM), measured at 600 MHz, 298 K.

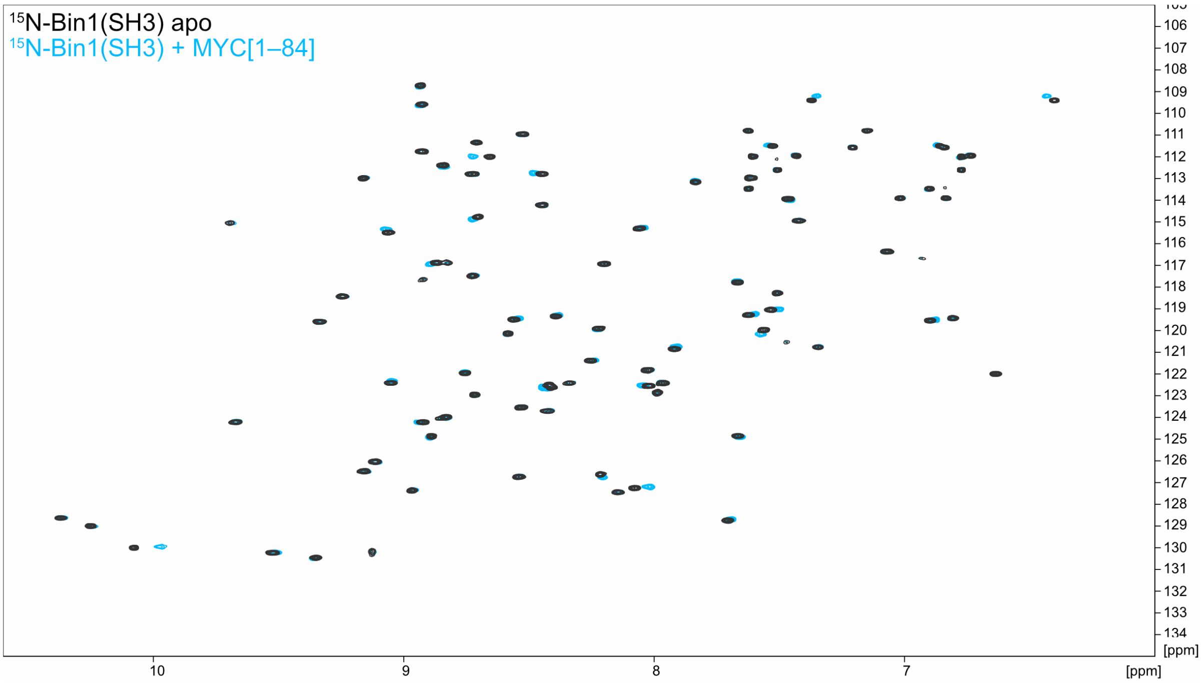

Figure S42. Spectrum overlay of apo Bin1(SH3) (black) with Bin1(SH3) + MYC[1–84] (blue). [^{15}N,^{1}H]-HSQC of apo* Bin1(SH3) (40 µM) (black) and [^{15}N,^{1}H]-HSQC of Bin1(SH3) (40 µM) in the presence of MYC[1–84] (80 µM) (blue). Spectra were measured at 600 MHz, 298 K. *Apo measurement taken on day of experiment, after dialysis in NMR buffer (**Figure S41**).

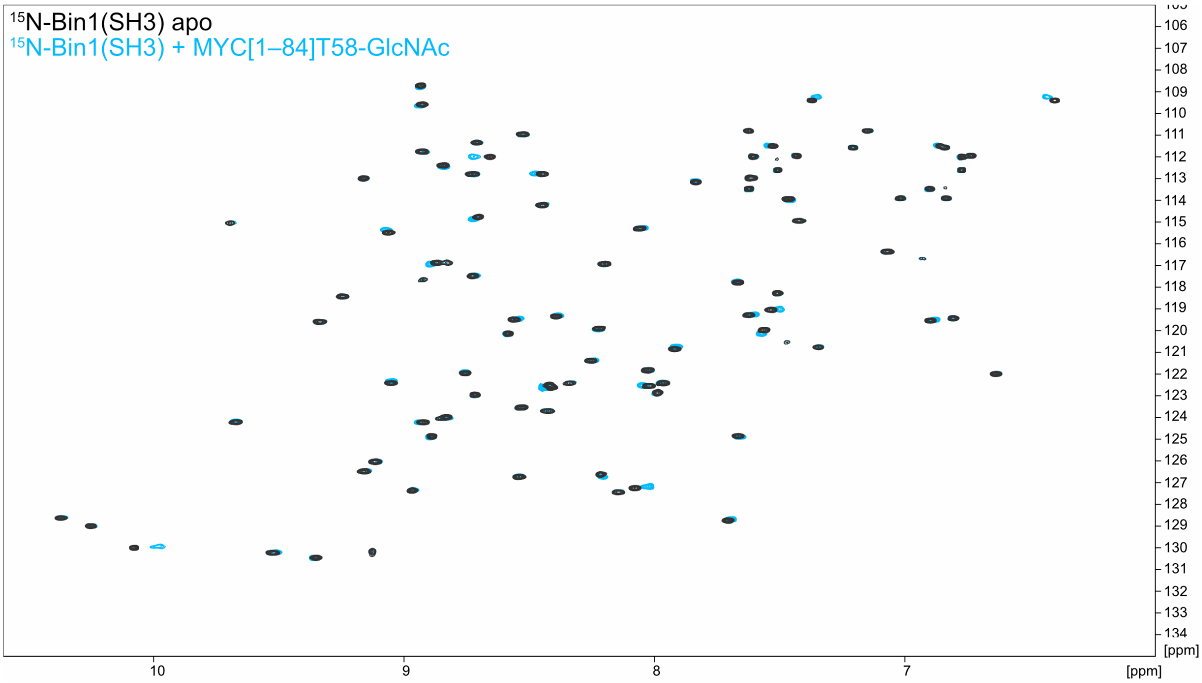

Figure S43. Spectrum overlay of apo Bin1(SH3) (black) with Bin1(SH3) + MYC[1–84]T58-GlcNAc (blue). [^{15}N,^{1}H]-HSQC of apo Bin1(SH3) (40 µM) (black) and [^{15}N,^{1}H]-HSQC of Bin1(SH3) (40 µM) in the presence of MYC[1–84]T58-GlcNAc (80 µM) (blue). Spectra were measured at 600 MHz, 298 K. *Apo measurement taken on day of experiment, after dialysis in NMR buffer (**Figure S41**).

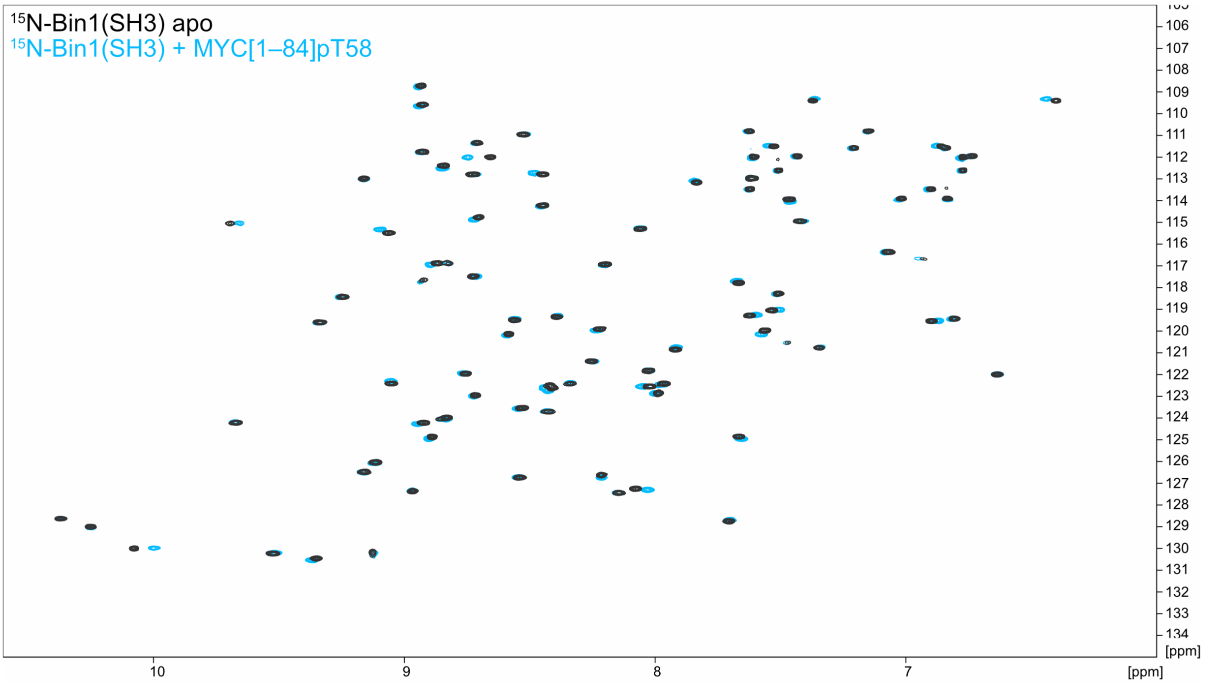

Figure S44. Spectrum overlay of apo Bin1(SH3) (black) with Bin1(SH3) + MYC[1–84]pT58 (blue). [^{15}N,^{1}H]-HSQC of apo* Bin1(SH3) (40 µM) (black) and [^{15}N,^{1}H]-HSQC of Bin1(SH3) (40 µM) in the presence of MYC[1–84]pT58 (80 µM) (blue). Spectra were measured at 600 MHz, 298 K. *Apo measurement taken on day of experiment, after dialysis in NMR buffer (**Figure S41**).

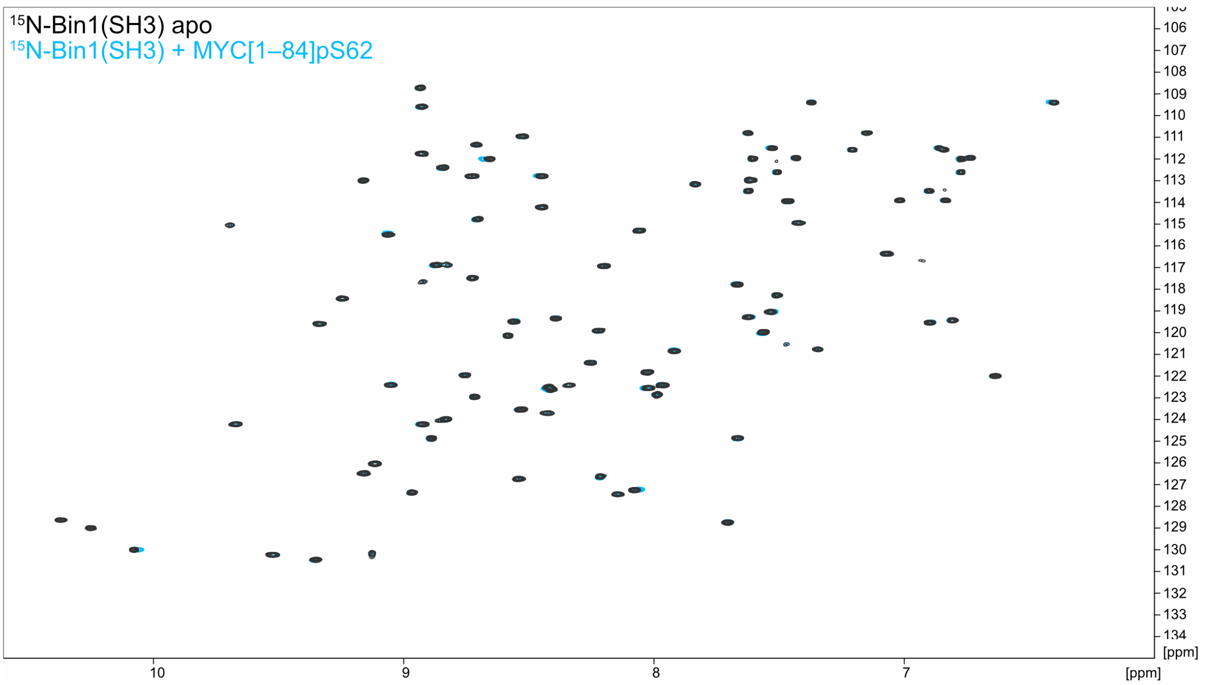

Figure S45. Spectrum overlay of apo Bin1(SH3) (black) with Bin1(SH3) + MYC[1–84]pS62 (blue). [^{15}N,^{1}H]-HSQC of apo* Bin1(SH3) (40 µM) (black) and [^{15}N,^{1}H]-HSQC of Bin1(SH3) (40 µM) in the presence of MYC[1–84]pS62 (80 µM) (blue). Spectra were measured at 600 MHz, 298 K. *Apo measurement taken on day of experiment, after dialysis in NMR buffer (**Figure S41**).

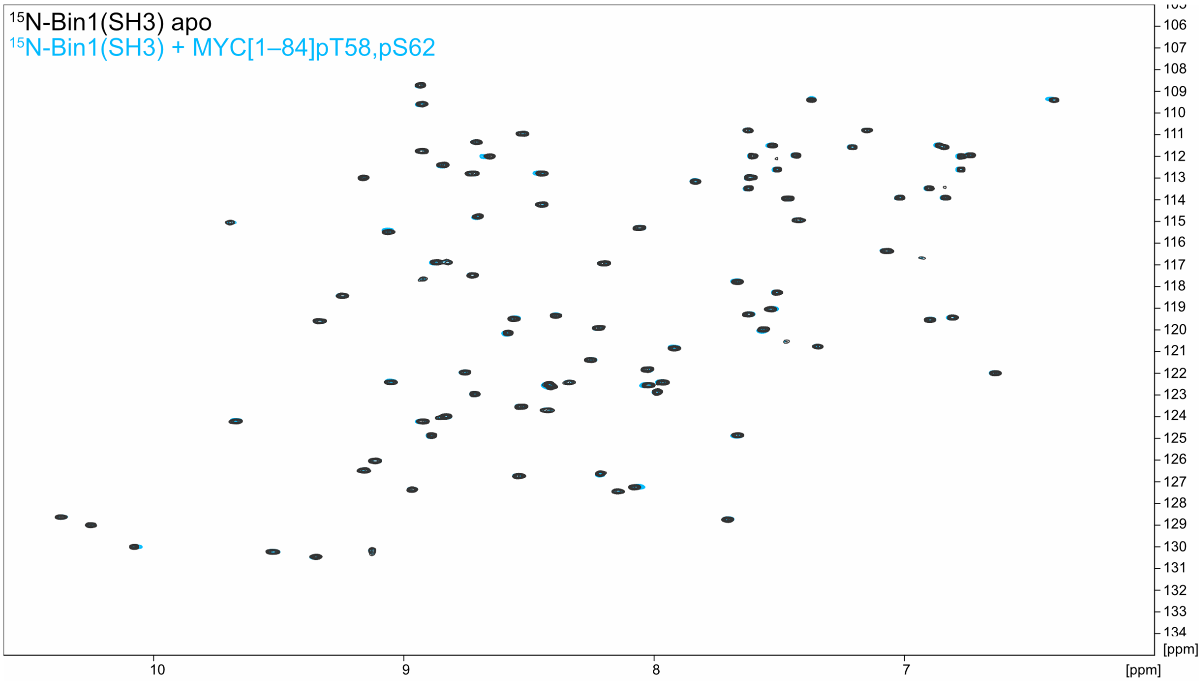

Figure S46. Spectrum overlay of apo Bin1(SH3) (black) with Bin1(SH3) + MYC[1–84]pT58,pS62 (blue). [^{15}N,^{1}H]-HSQC of apo* Bin1(SH3) (40 µM) (black) and [^{15}N,^{1}H]-HSQC of Bin1(SH3) (40 µM) in the presence of MYC[1–84]pT58,pS62 (80 µM) (blue). Spectra were measured at 600 MHz, 298 K. *Apo measurement taken on day of experiment, after dialysis in NMR buffer (**Figure S41**).

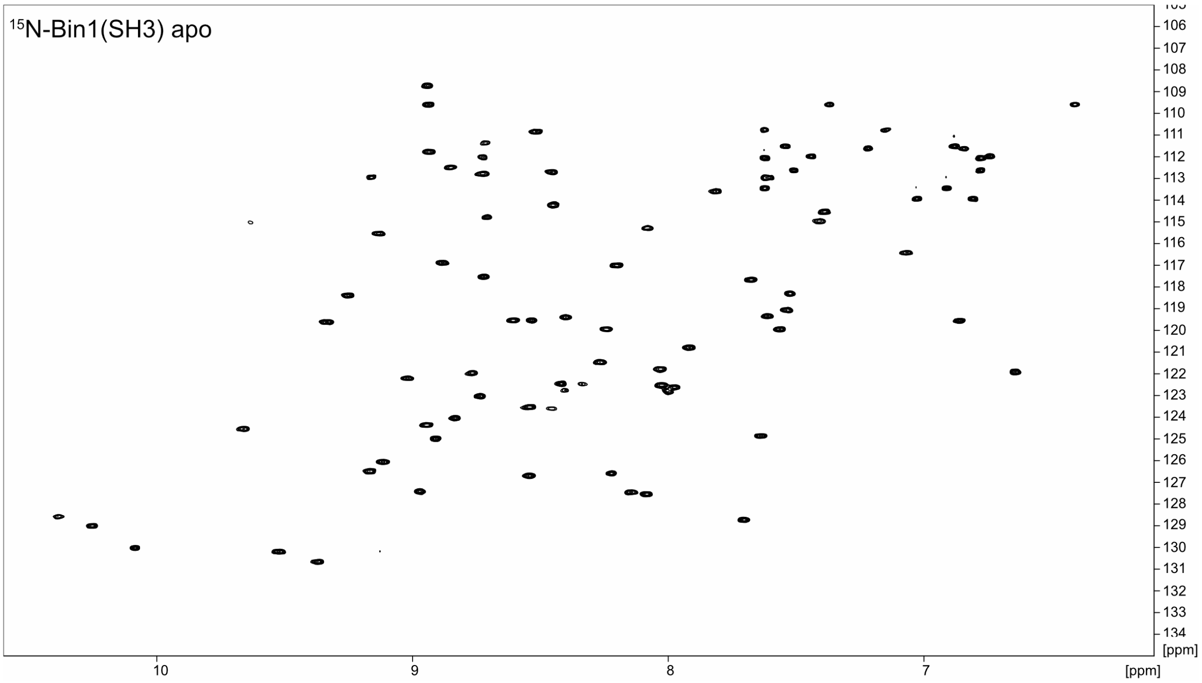

Figure S47. Bin1(SH3) apo measurement, day of MYC[55–68], MYC[55–68]pS62, and MYC[55–68]pT58,pS62 experiments, after dialysis in NMR buffer. [^{15}N,^{1}H]-HSQC of Bin1(SH3) (40 µM), measured at 600 MHz, 298 K.

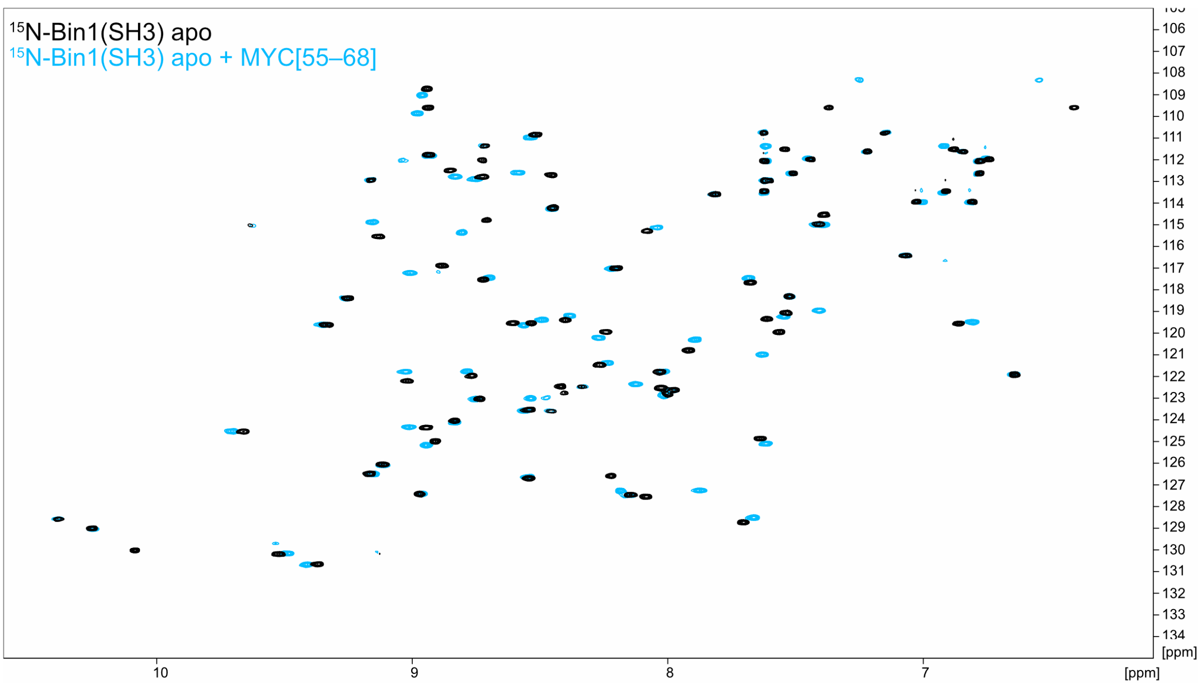

Figure S48. Spectrum overlay of apo Bin1(SH3) (black) with Bin1(SH3) + MYC[55–68] (blue). [^{15}N,^{1}H]-HSQC of apo* Bin1(SH3) (40 µM) (black) and [^{15}N,^{1}H]-HSQC of Bin1(SH3) (40 µM) in the presence of MYC[55–68] (80 µM) (blue). Spectra were measured at 600 MHz, 298 K. *Apo measurement taken on day of experiment, after dialysis in NMR buffer (**Figure S47**).

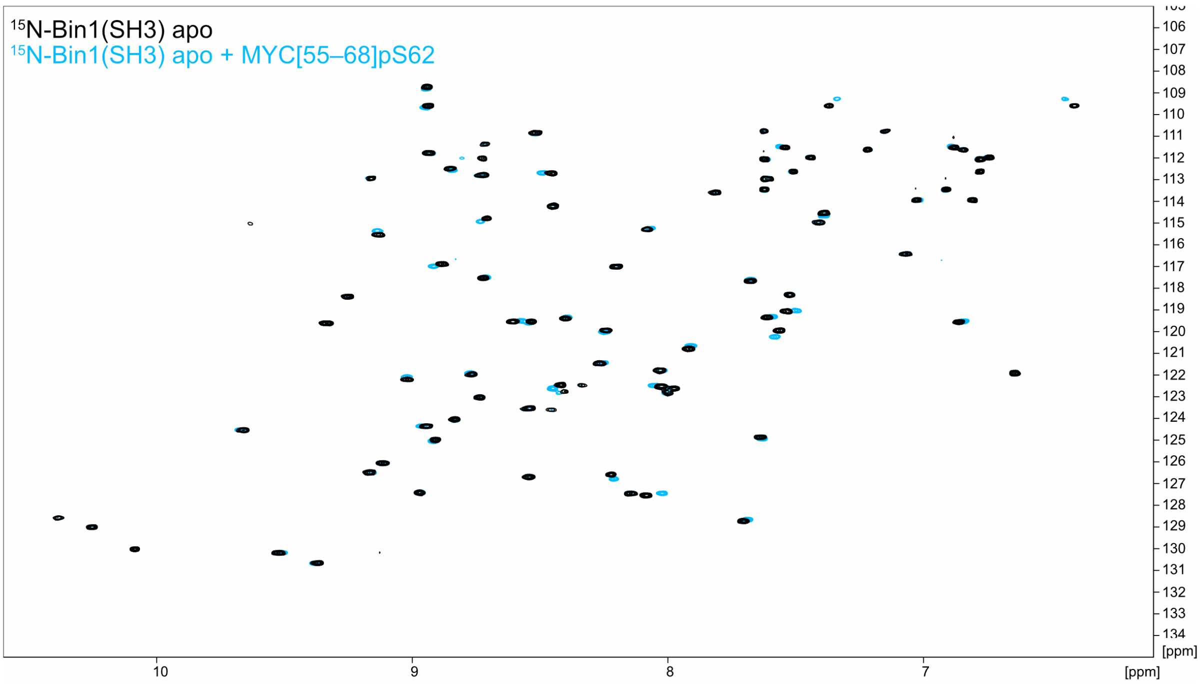

Figure S49. Spectrum overlay of apo Bin1(SH3) (black) with Bin1(SH3) + MYC[55–68]pS62 (blue). [^{15}N,^{1}H]-HSQC of apo* Bin1(SH3) (40 µM) (black) and [^{15}N,^{1}H]-HSQC of Bin1(SH3) (40 µM) in the presence of MYC[55–68]pS62 (80 µM) (blue). Spectra were measured at 600 MHz, 298 K. *Apo measurement taken on day of experiment, after dialysis in NMR buffer (**Figure S47**).

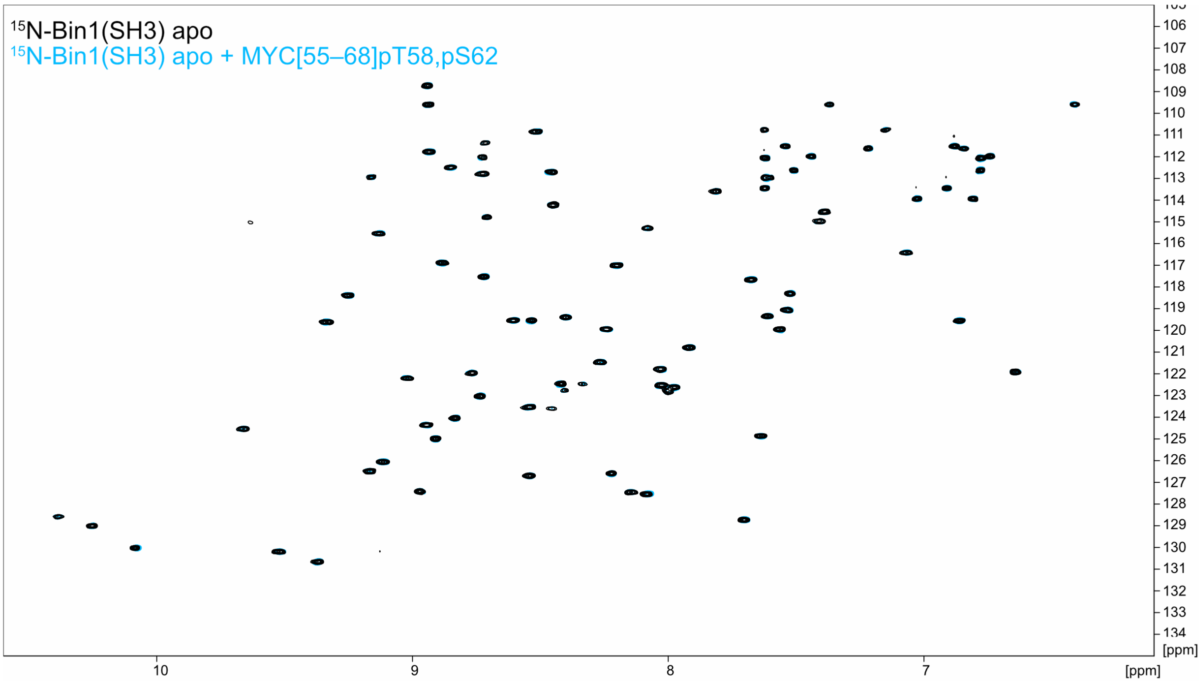

Figure S50. Spectrum overlay of apo Bin1(SH3) (black) with Bin1(SH3) + MYC[55–68]pT58,pS62 (blue). [^{15}N,^{1}H]-HSQC of apo* Bin1(SH3) (40 µM) (black) and [^{15}N,^{1}H]-HSQC of Bin1(SH3) (40 µM) in the presence of MYC[55–68]pT58,pS62 (80 µM) (blue). Spectra were measured at 600 MHz, 298 K. *Apo measurement taken on day of experiment, after dialysis in NMR buffer (**Figure S47**).

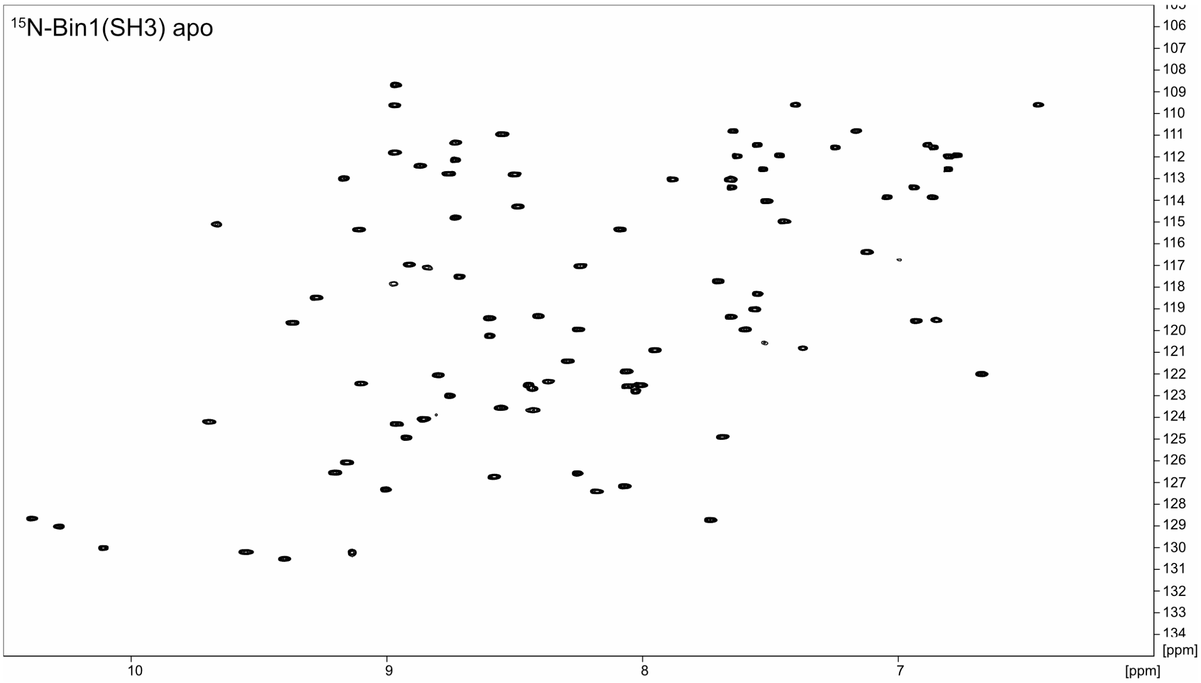

Figure S51. Bin1(SH3) apo measurement, day of MYC[55–68]pT58 and MYC[55–68]T58-GlcNAc experiments, after dialysis in NMR buffer. [^{15}N,^{1}H]-HSQC of Bin1(SH3) (40 µM), measured at 600 MHz, 298 K.

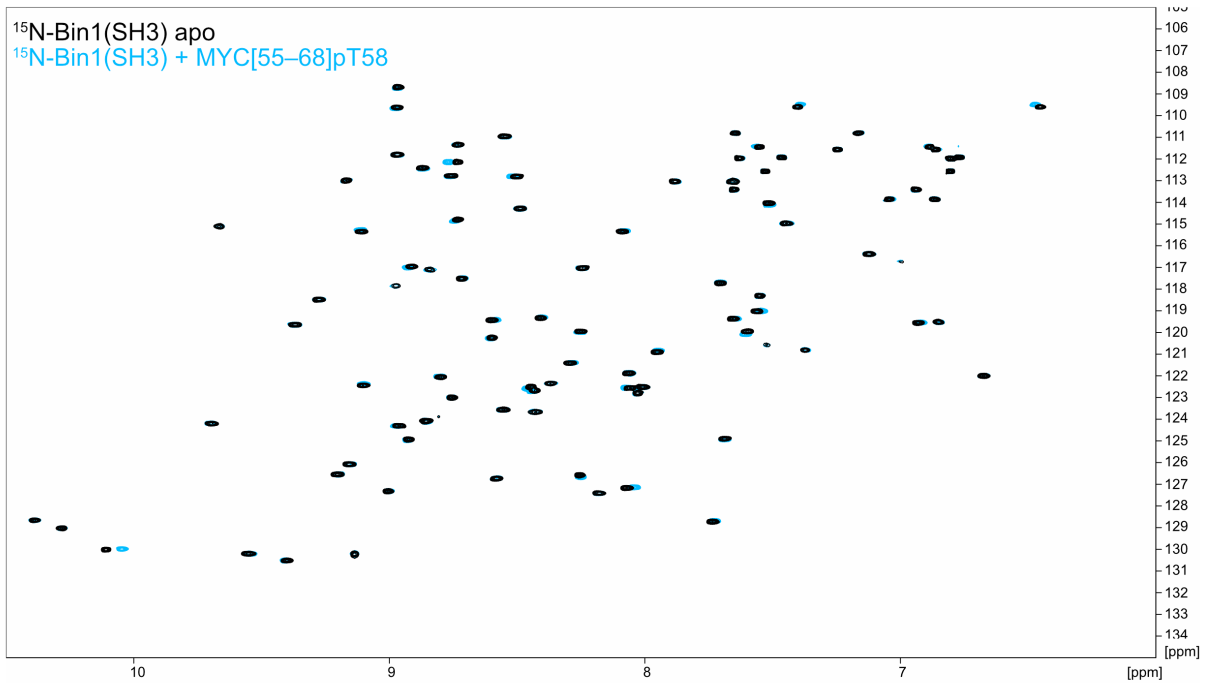

Figure S52. Spectrum overlay of apo Bin1(SH3) (black) with Bin1(SH3) + MYC[55–68]pT58 (blue). [^{15}N,^{1}H]-HSQC of apo* Bin1(SH3) (40 µM) (black) and [^{15}N,^{1}H]-HSQC of Bin1(SH3) (40 µM) in the presence of MYC[55–68]pT58 (80 µM) (blue). Spectra were measured at 600 MHz, 298 K. *Apo measurement taken on day of experiment, after dialysis in NMR buffer (**Figure S51**). MYC[55–68]pT58 was unable to be dialyzed, therefore the peptide was directly dissolved in the NMR buffer (used for Bin1(SH3) dialysis) and the pH was manually adjusted to pH 6.5.

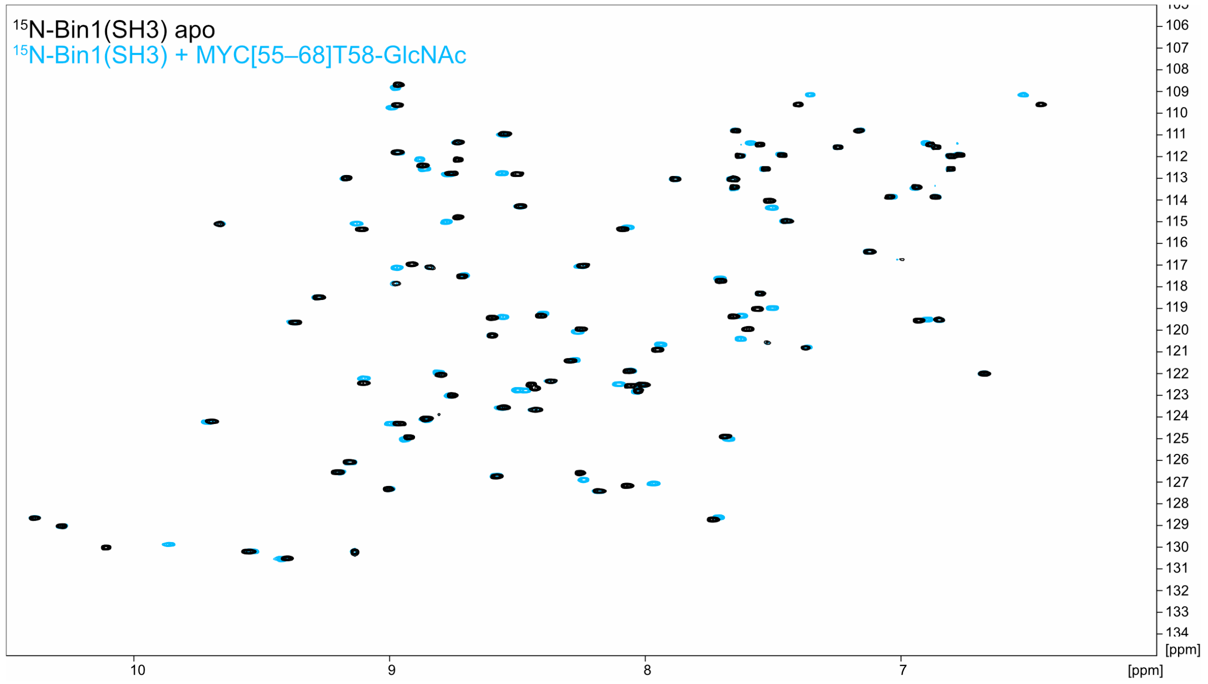

Figure S53. Spectrum overlay of apo Bin1(SH3) (black) with Bin1(SH3) + MYC[55–68]T58-GlcNAc (blue). [^{15}N,^{1}H]-HSQC of apo* Bin1(SH3) (40 µM) (black) and [^{15}N,^{1}H]-HSQC of Bin1(SH3) (40 µM) in the presence of MYC[55–68]T58-GlcNAc (80 µM) (blue). Spectra were measured at 600 MHz, 298 K. *Apo measurement taken on day of experiment, after dialysis in NMR buffer (**Figure S51**). MYC[55–68]T58-GlcNAc was unable to be dialyzed, therefore the peptide was directly dissolved in the NMR buffer (used for Bin1(SH3) dialysis) and the pH was manually adjusted to pH 6.5.

8 CSPs of Bin1(SH3) with MYC[1–84] analogues (scaled)

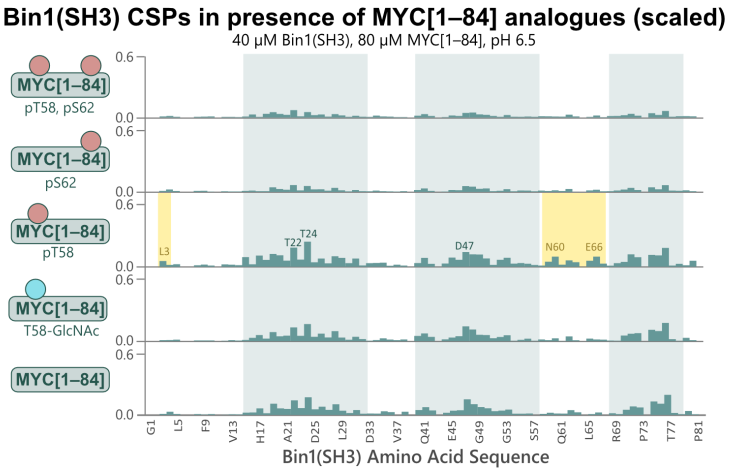

Figure S54. CSPs (ppm) of ^{15}N-Bin1(SH3) backbone NH peaks in the presence of each MYC[1–84] analogue, measured by [^{15}N,^{1}H]-HSQC, scaled to match Fig. 3B.

9 Comparison of AFPS to Batch-SPPS: Reagents, solvent, waste, and synthesis time

		AFPS		Batch-SPPS	
		"Single Couplings"	"Double Couplings"	Single Couplings	Double Couplings
Synthesis scale (μmol)		30		30	
Resin Swelling	DMF (mL)	3		3	
	Time (min)	0.5		10	
Coupling	Fmoc-AA-OH (eq.)	43	70	5	10
	Activating agent (eq.)	40	66	4.5	9
	DIPEA (mL)	0.32	0.52	0.05	0.1
	DMF (mL)	6.4	10.4	3	6
	Time (min)	0.42	0.67	30	60
Deprotection	Piperidine solution (mL)	6.4		6	
	Time (min)	0.42		10	
Washing	DMF (total, mL)	32		27	36
	Time (total, min)	1		1	2
Per Coupling Cycle	**Liquid Waste (mL)**	45.1	49.3	36.1	48.1
	**Time (min)**	1.8	2.1	41	72

Table S3. Comparison of reagent and solvent usage, synthesis time, and waste generated by AFPS compared to a typical batch-SPPS procedure. AFPS methods used to calculate these values are found in **Section 3.1**. Batch-SPPS values in this table are calculated based on the following method: Scale: 30 μmol. Couplings: Fmoc-AA-OH (5 eq.), activator (4.5 eq.), DIPEA (50 μL), DMF (3 mL), 1 × 30 min (single coupling) or 2 × 30 min (double coupling). Deprotection: 20% piperidine in DMF (3 mL), 2 × 5 min. Washing: 3 × 3 mL DMF after each coupling and deprotection. Waste generated and time required per coupling cycle (bottom rows) are calculated without inclusion of resin swelling steps. "Single Couplings" for AFPS indicate short couplings, i.e. 8 strokes, 6.4 mL (amino acids D, E, F, G, I, K, L, M, P, S, W, and Y). "Double couplings" for AFPS indicate longer couplings, i.e. 13 strokes, 10.4 mL (amino acids A, C, H, N, Q, R, S, T, and V).

	Synthesis of a 20 AA peptide, single couplings only	
	**AFPS**	**Batch-SPPS**
**Liquid Waste (mL)**	905	724
**Time (h)**	0.8	13.8

Table S4. Example of waste generated (mL) and synthesis time (h) for a 20 AA peptide prepared by AFPS or batch-SPPS, applying single couplings only. Calculated using **Table S3**.

10 References

[25] N. Hartrampf, A. Saebi, M. Poskus, Z. P. Gates, A. J. Callahan, A. E. Cowfer, S. Hanna, S. Antilla, C. K. Schissel, A. J. Quartararo, X. Ye, A. J. Mijalis, M. D. Simon, A. Loas, S. Liu, C. Jessen, T. E. Nielsen, B. L. Pentelute, *Science* **2020**, *368*, 980–987.

[30] A. Pineda-Lucena, C. S. W. Ho, D. Y. L. Mao, Y. Sheng, R. C. Laister, R. Muhandiram, Y. Lu, B. T. Seet, S. Katz, T. Szyperski, L. Z. Penn, C. H. Arrowsmith, *J. Mol. Biol.* **2005**, *351*, 182–194.

[51] D. Stoll, *LCGC Eur.* **2021**, *34*, 96–100.

[53] A. Wakamatsu, K. Morimoto, M. Shimizu, S. Kudoh, *J. Sep. Sci.* **2005**, *28*, 1823–1830.

[54] H. Tossavainen, O. Aitio, M. Hellman, K. Saksela, P. Permi, *J. Biol. Chem.* **2016**, *291*, 16307–16317.

[55] M. Sattler, J. Schleucher, C. Griesinger, *Prog. Nucl. Magn. Reson. Spectrosc.* **1999**, *34*, 93–158.

[56] J. Keeler, R. T. Clowes, A. L. Davis, E. D. Laue, *Methods Enzymol.* **1994**, *239*, 145–207.

[57] L. Kay, P. Keifer, T. Saarinen, *J. Am. Chem. Soc.* **1992**, *114*, 10663–10665.

[58] M. P. Williamson, *Prog. Nucl. Magn. Reson. Spectrosc.* **2013**, *73*, 1–16.

[59] A. Pineda-Lucena, C. H. Arrowsmith, *J. Biomol. NMR* **2001**, *19*, 191–192.
